# Supplementary figures and images for: Absence of CEP78 causes photoreceptor and sperm flagella impairments in mice and a human individual
Source: eLife. 2023 Feb 9;12:e76157. doi: 10.7554/eLife.76157 (PMC9984195; doi:10.7554/eLife.76157)

## Slide 1
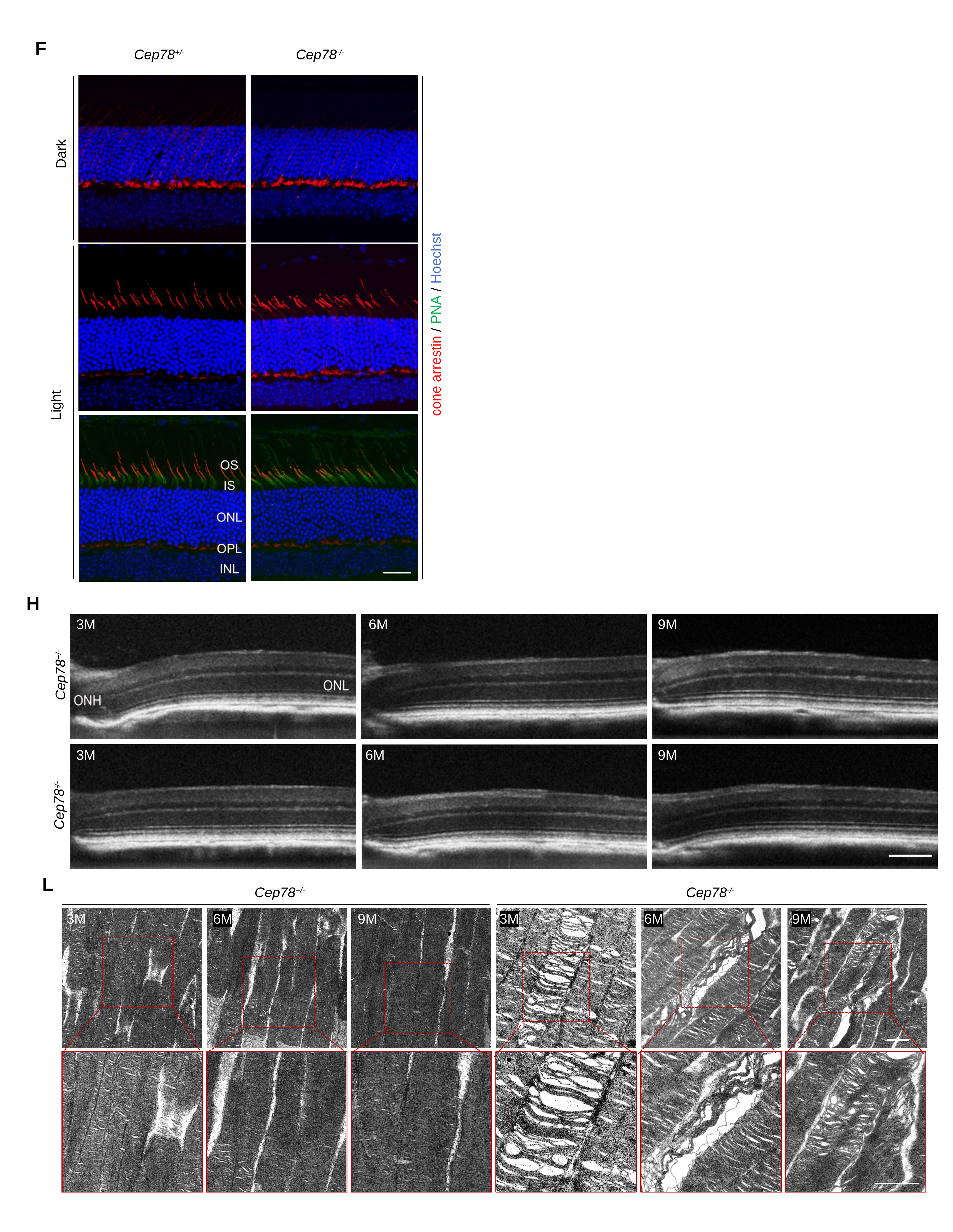

F
cone arrestin / PNA / Hoechst
Cep78-/-
Cep78+/-
Dark
Light
H
3M
6M
9M
Cep78+/-
Cep78-/-
3M
6M
9M
L
Cep78+/-
Cep78-/-
3M
6M
9M
3M
6M
9M

Supplement: Figure 1—source data 2. [file elife-76157-fig1-data2.zip › Figure 1-source data 2.pptx]

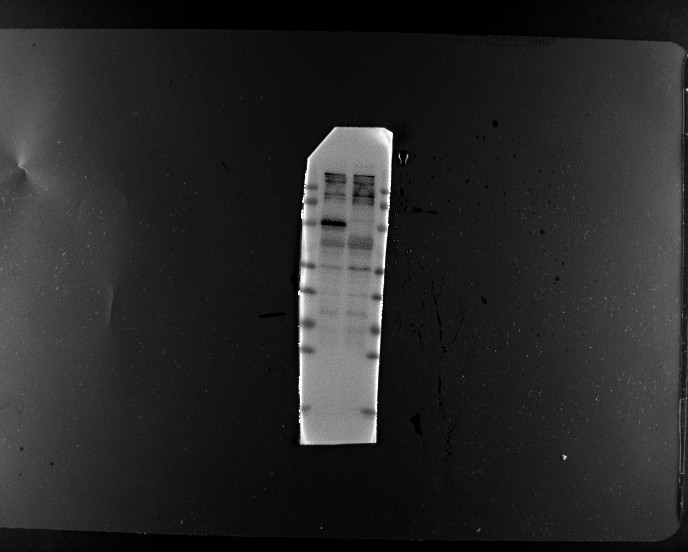

Supplement: Figure 1—figure supplement 1—source data 1. [file elife-76157-fig1-figsupp1-data1.zip › Figure 1-figure supplement 1-source data 1/Figure 1-figure supplement 1D-row 1.tif]

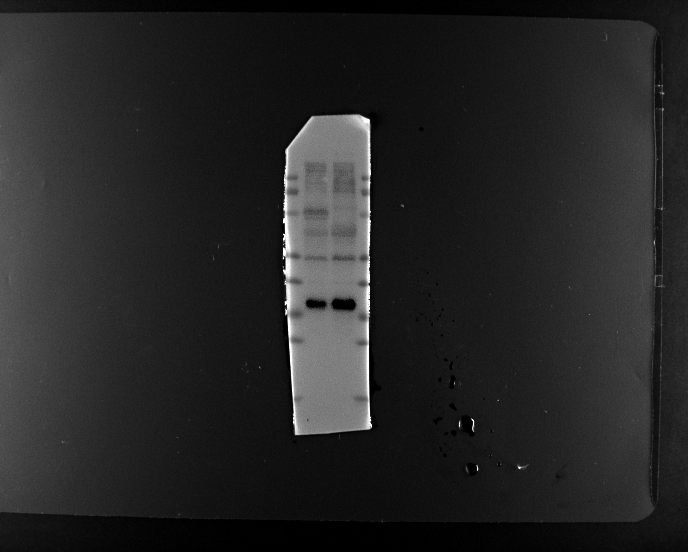

Supplement: Figure 1—figure supplement 1—source data 1. [file elife-76157-fig1-figsupp1-data1.zip › Figure 1-figure supplement 1-source data 1/Figure 1-figure supplement 1D-row 2.tif]

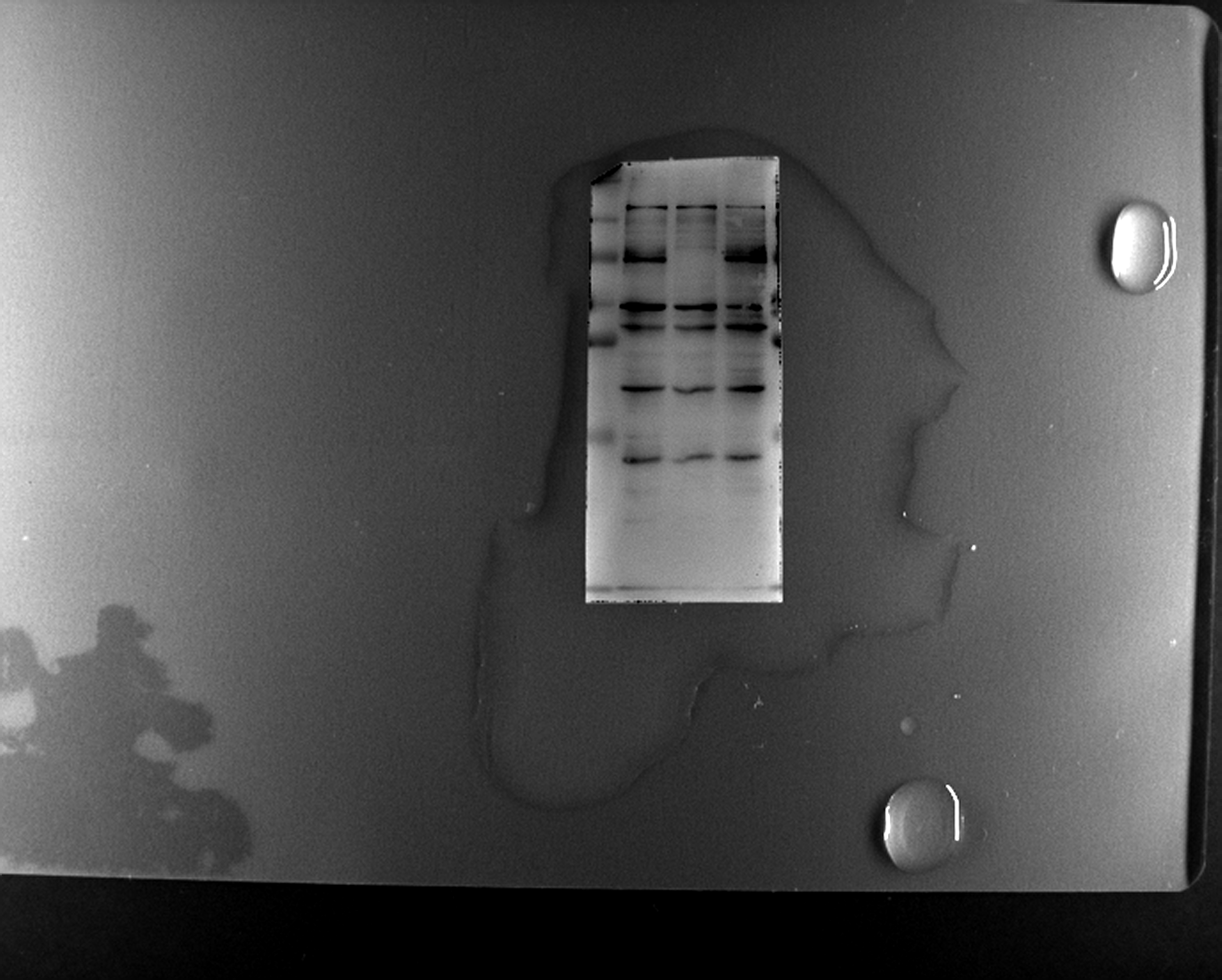

Supplement: Figure 1—figure supplement 1—source data 1. [file elife-76157-fig1-figsupp1-data1.zip › Figure 1-figure supplement 1-source data 1/Figure 1-figure supplement 1C-row 1.tif]

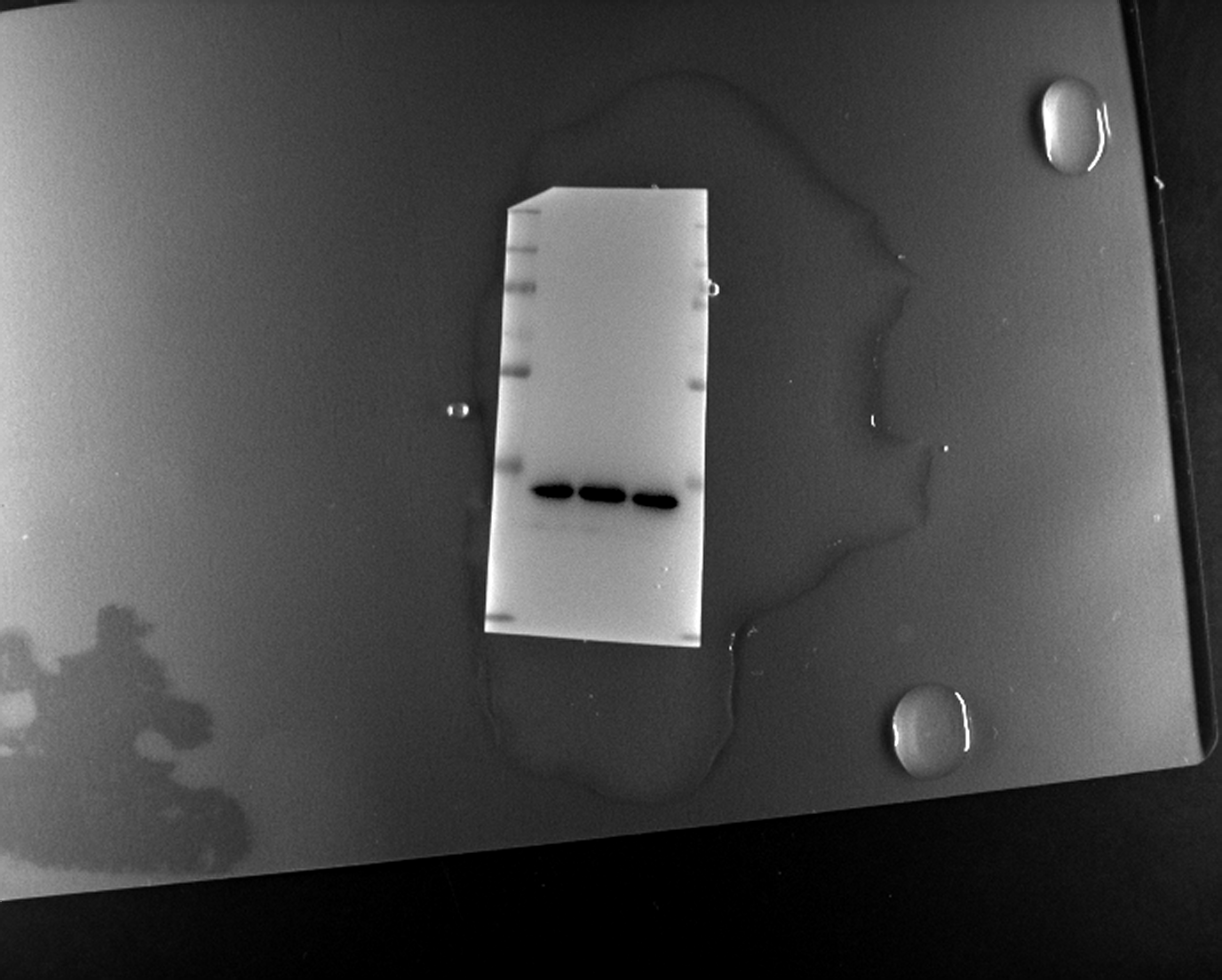

Supplement: Figure 1—figure supplement 1—source data 1. [file elife-76157-fig1-figsupp1-data1.zip › Figure 1-figure supplement 1-source data 1/Figure 1-figure supplement 1C-row 2.tif]

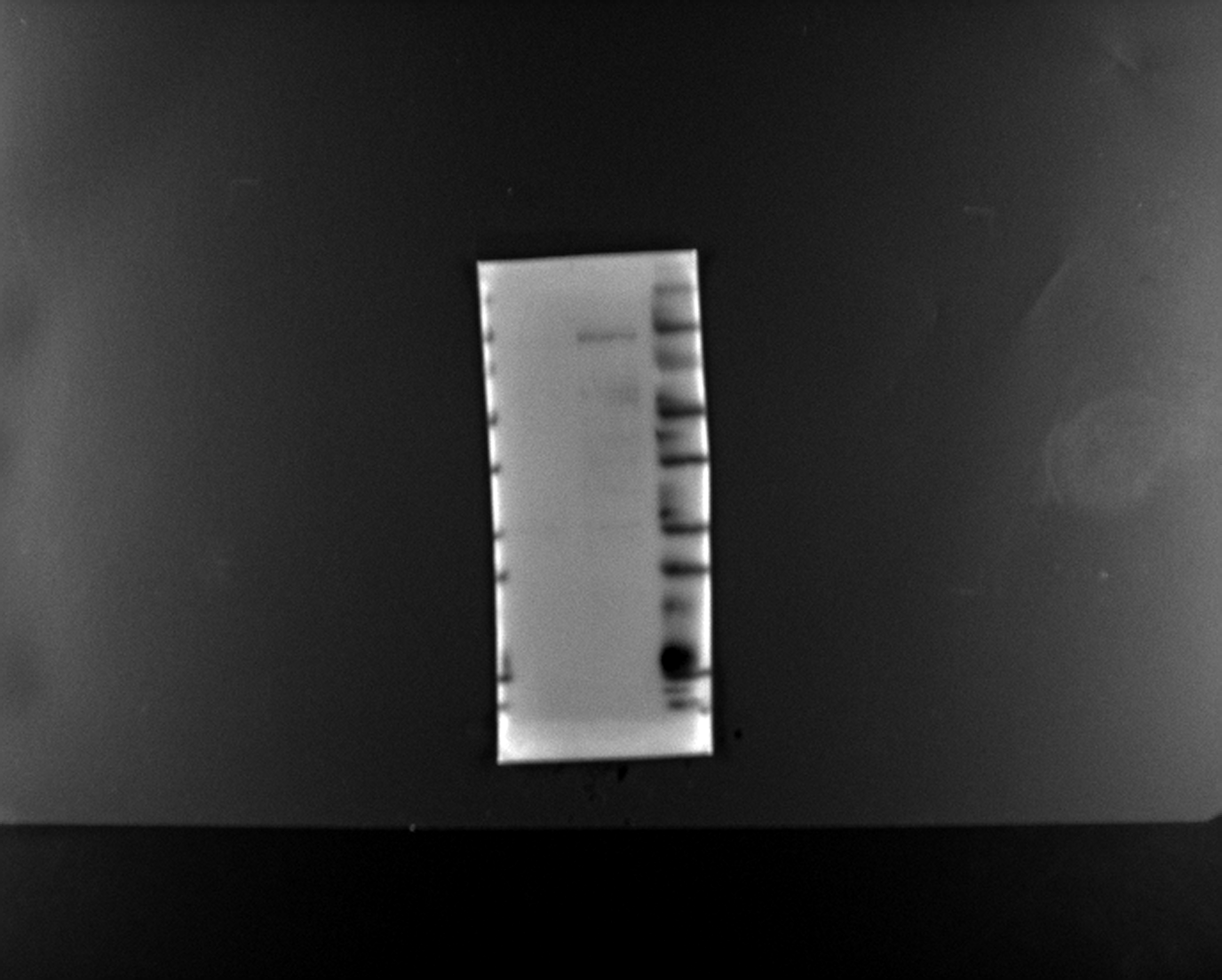

Supplement: Figure 6—figure supplement 1—source data 1. [file elife-76157-fig6-figsupp1-data1.zip › Figure 6-figure supplement 1-source data 1/Figure 6-figure supplement 1-Row 1.tif]

Figure 6-figure supplement 1-Source data

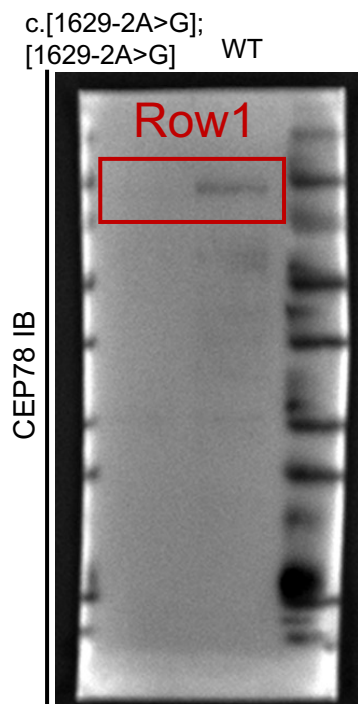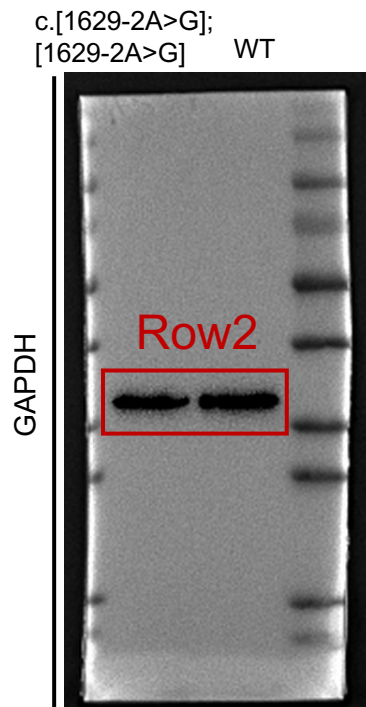

Supplement: Figure 6—figure supplement 1—source data 1. [file elife-76157-fig6-figsupp1-data1.zip › Figure 6-figure supplement 1-source data 1/Figure 6-figure supplement 1-labeled.pdf]

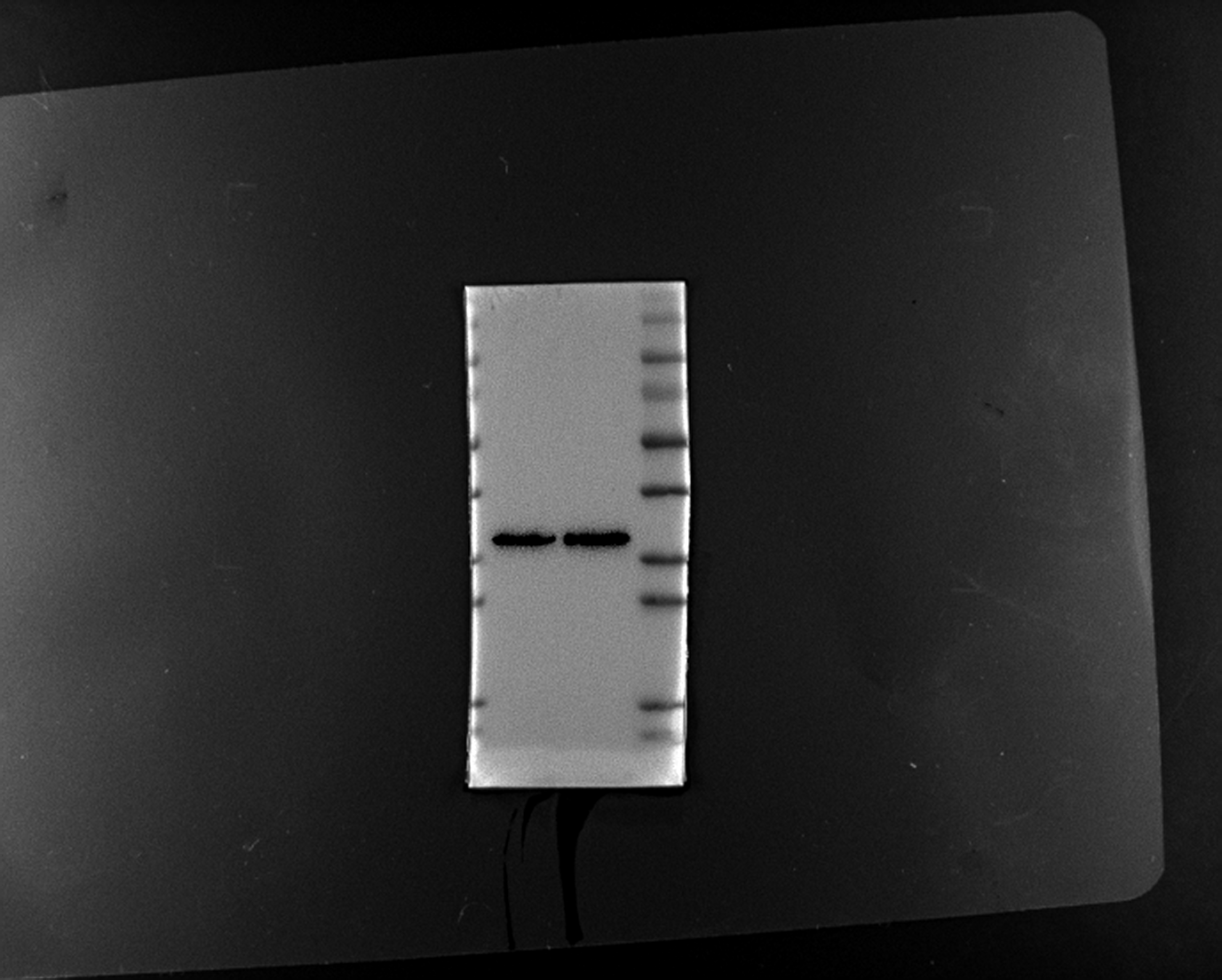

Supplement: Figure 6—figure supplement 1—source data 1. [file elife-76157-fig6-figsupp1-data1.zip › Figure 6-figure supplement 1-source data 1/Figure 6-figure supplement 1-Row2.tif]

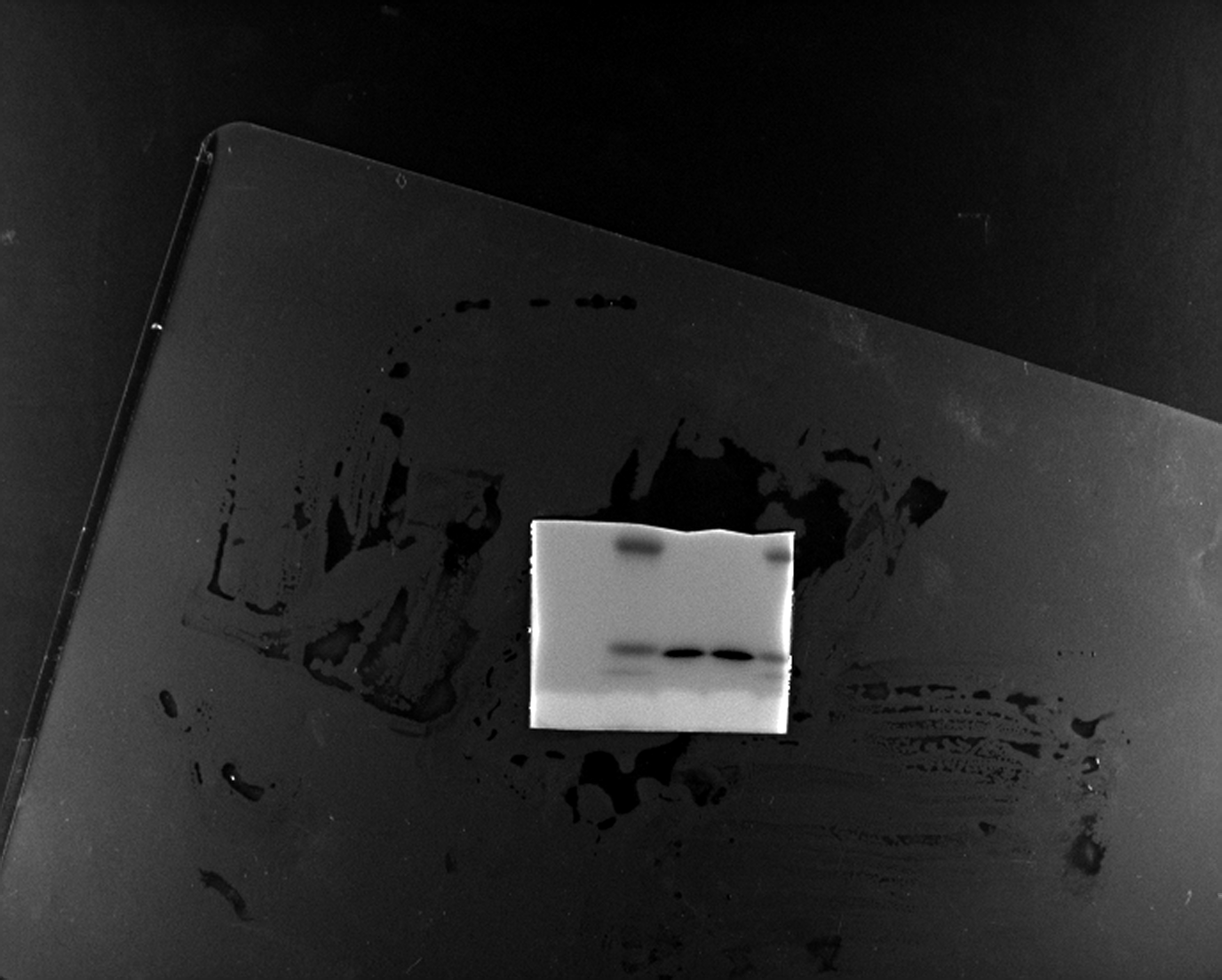

Supplement: Figure 7—source data 1. [file elife-76157-fig7-data1.zip › Figure 7-source data 1/Figure 7D-row 7.tif]

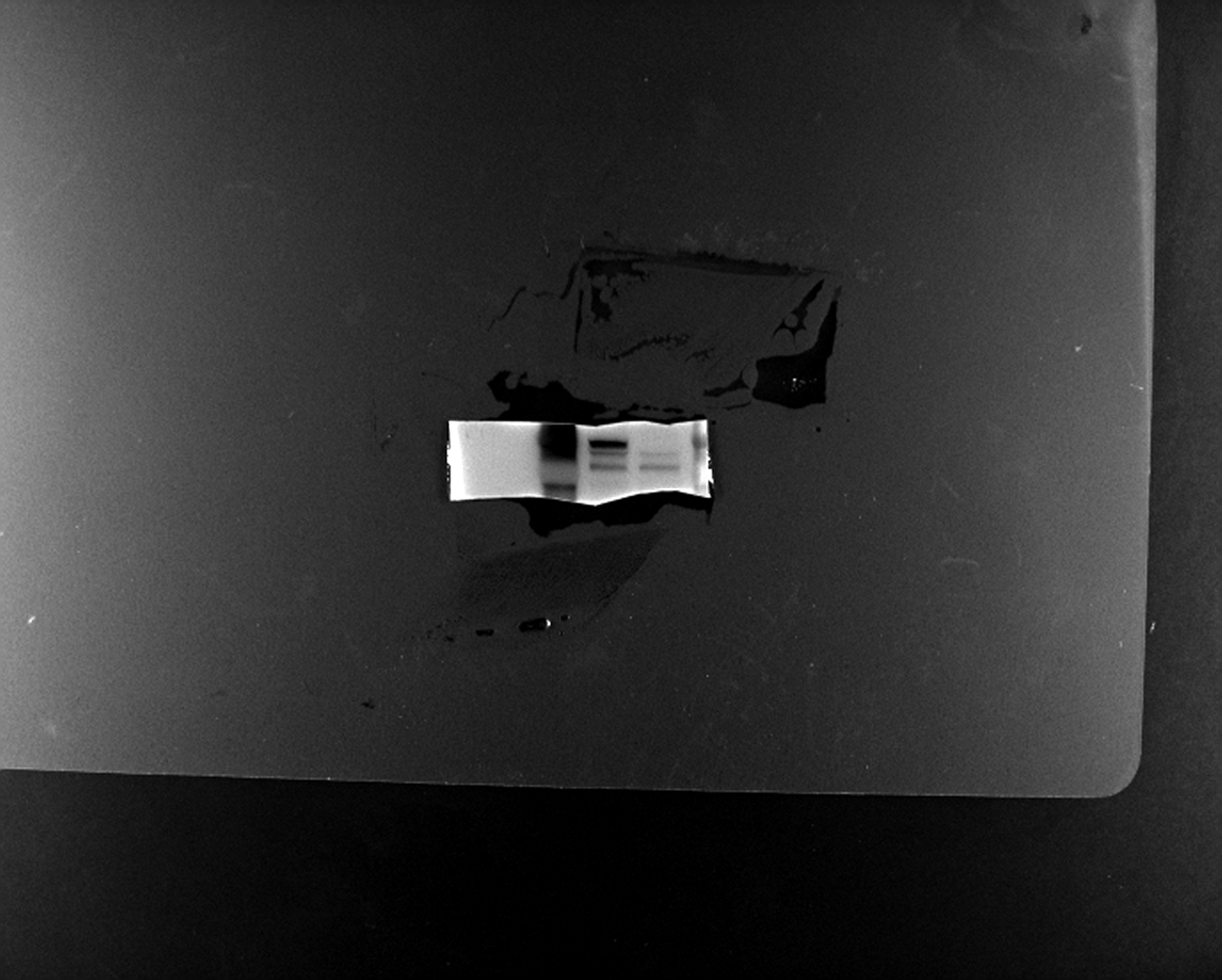

Supplement: Figure 7—source data 1. [file elife-76157-fig7-data1.zip › Figure 7-source data 1/Figure 7D-row 6.tif]

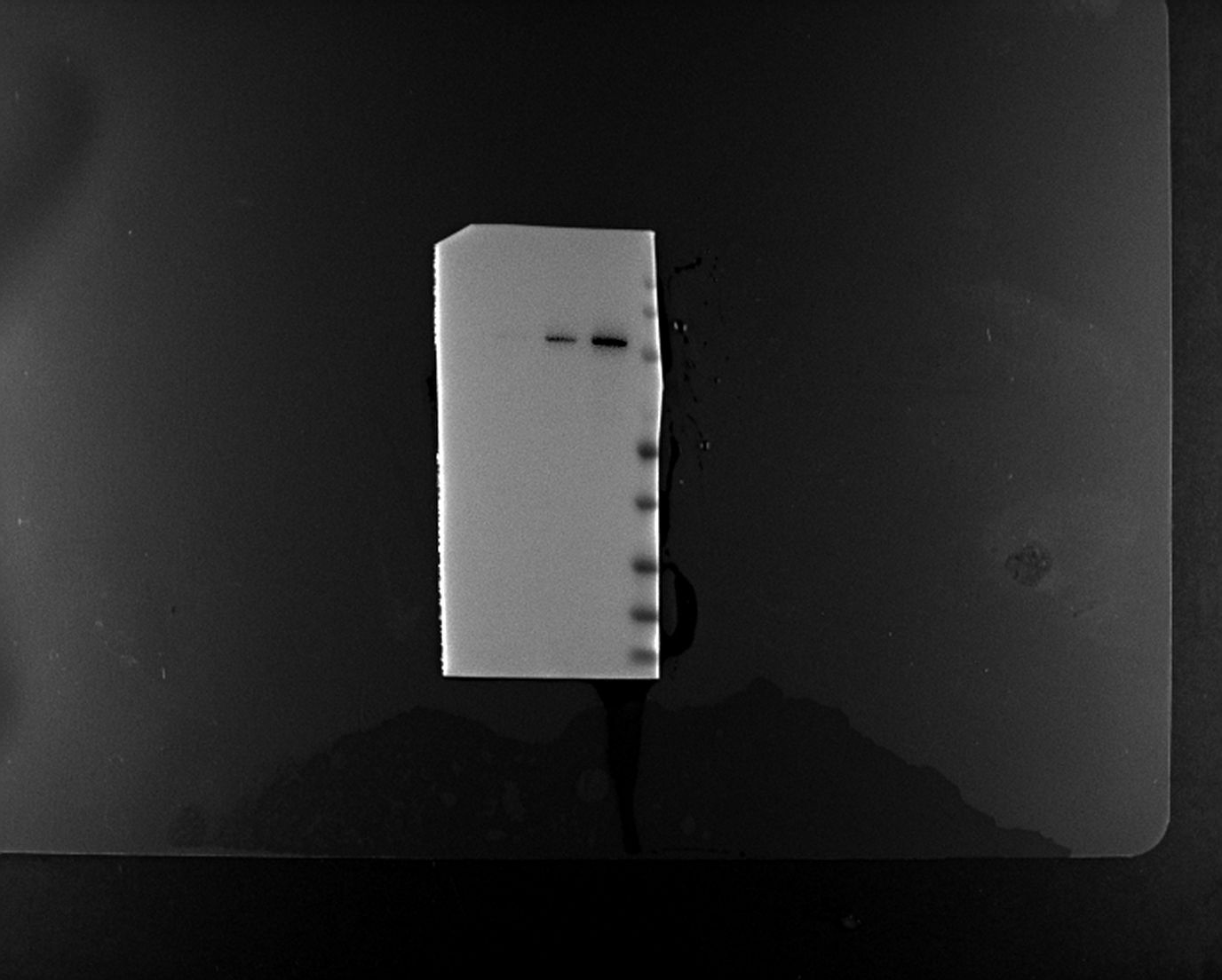

Supplement: Figure 7—source data 1. [file elife-76157-fig7-data1.zip › Figure 7-source data 1/Figure 7B-row 1.tif]

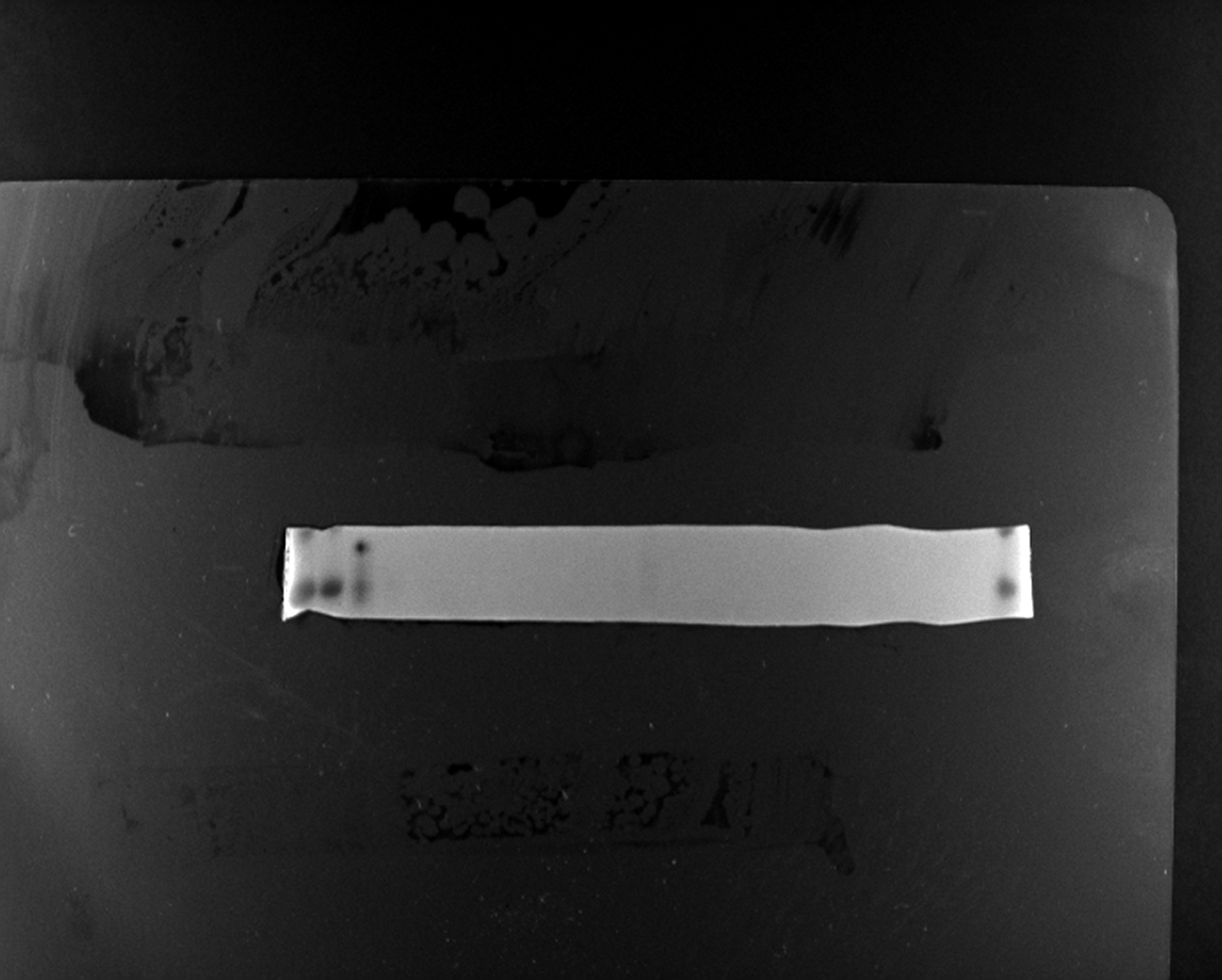

Supplement: Figure 7—source data 1. [file elife-76157-fig7-data1.zip › Figure 7-source data 1/Figure 7E-E-row unshown.tif]

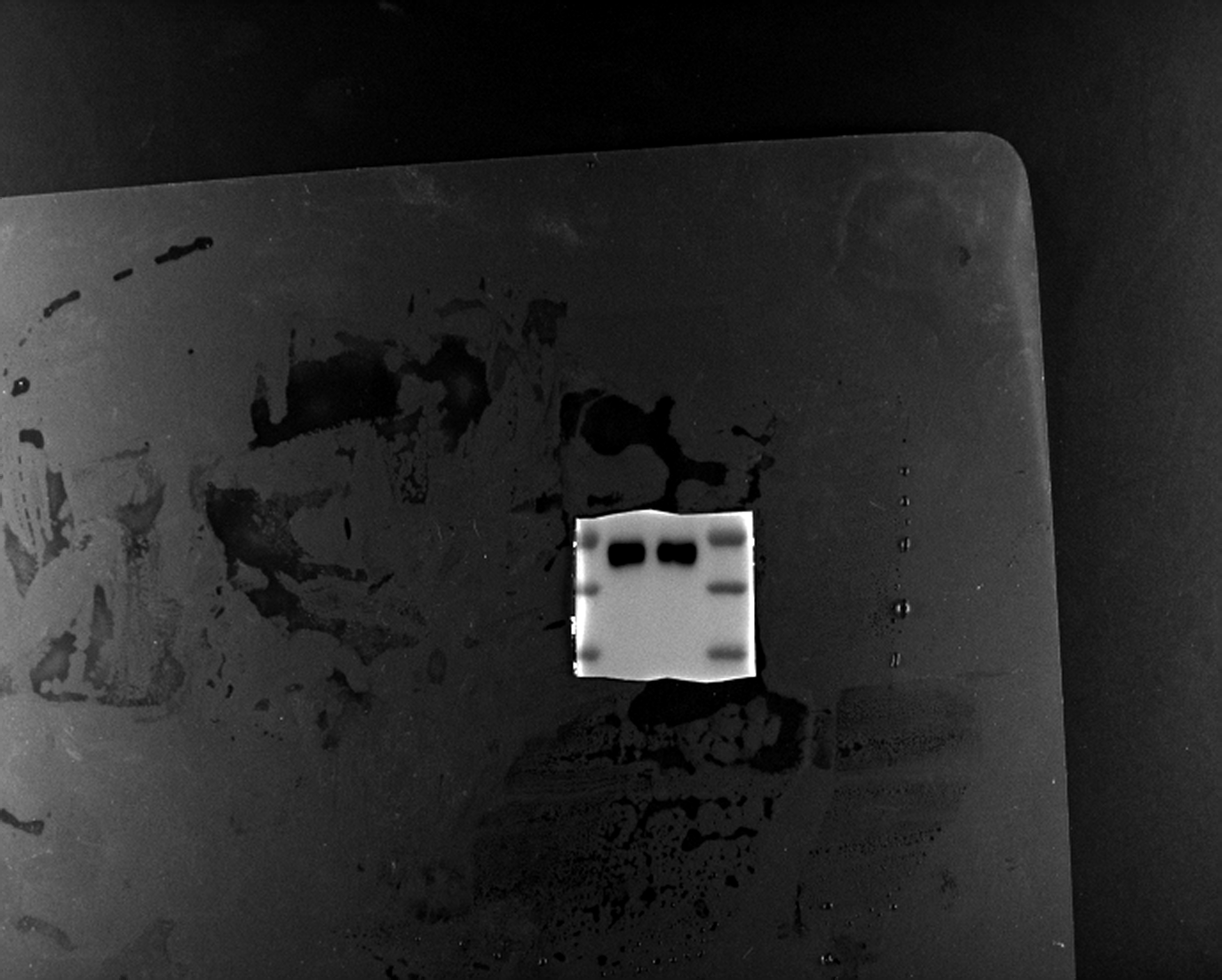

Supplement: Figure 7—source data 1. [file elife-76157-fig7-data1.zip › Figure 7-source data 1/Figure 7D-row 4.tif]

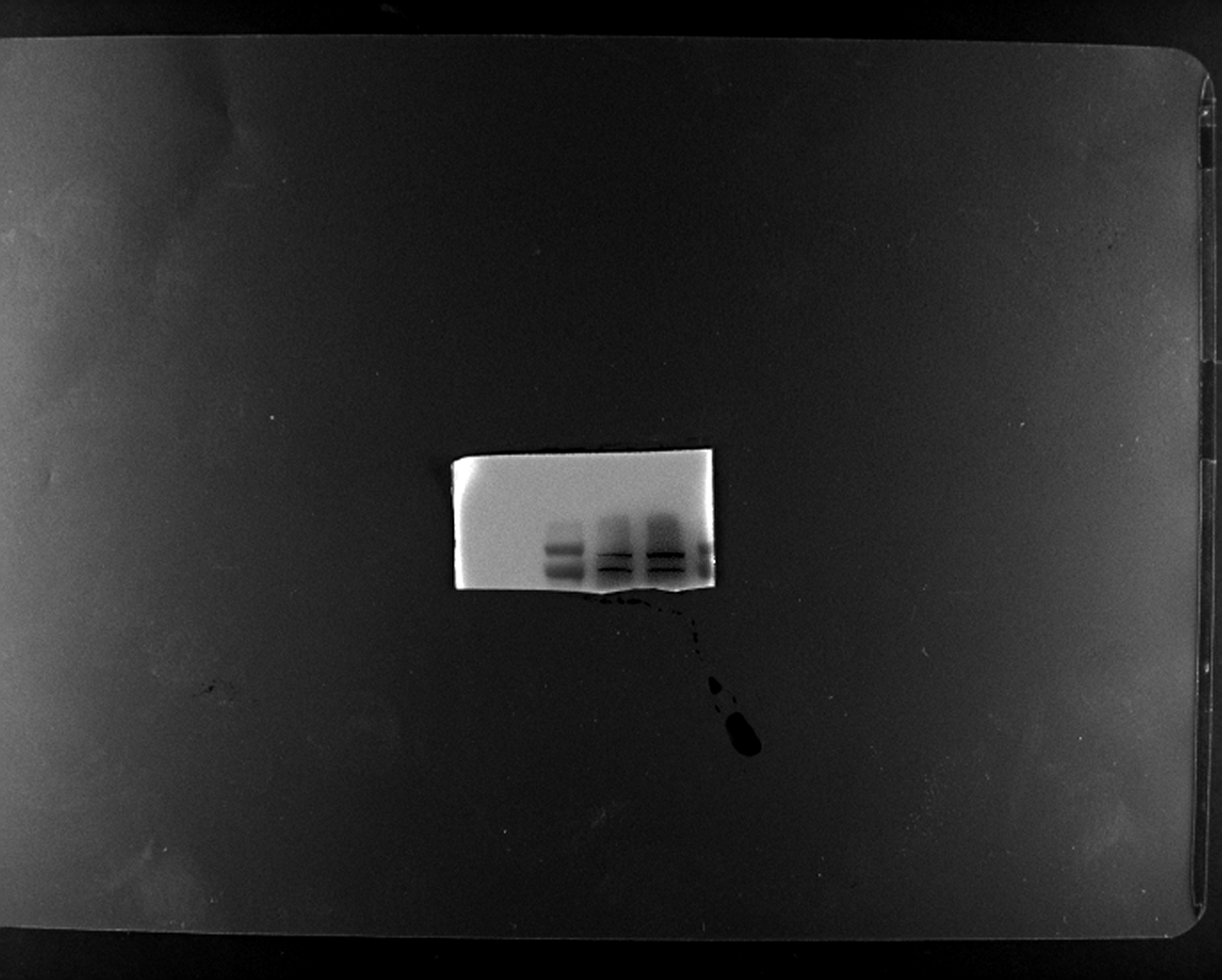

Supplement: Figure 7—source data 1. [file elife-76157-fig7-data1.zip › Figure 7-source data 1/Figure 7D-row 5.tif]

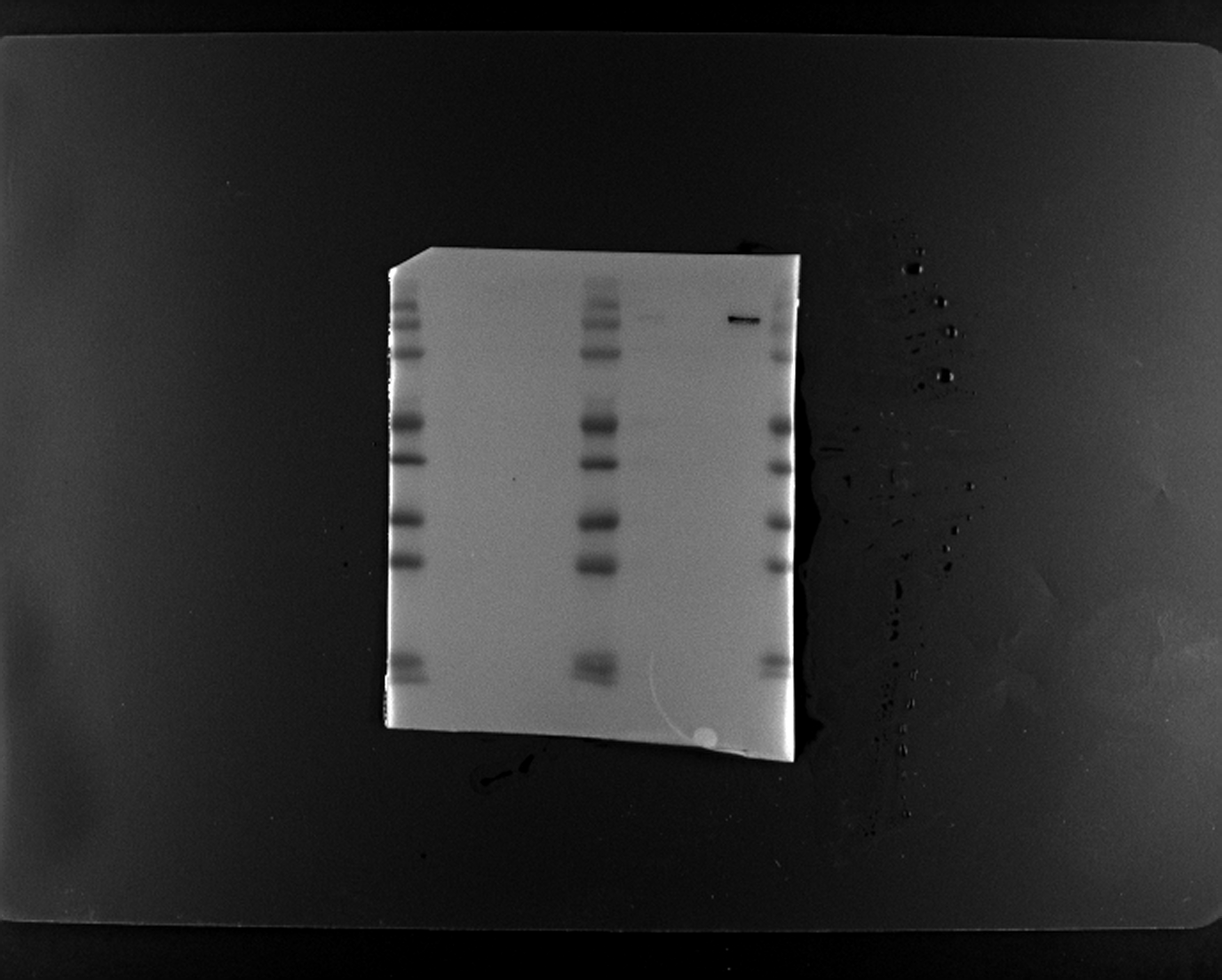

Supplement: Figure 7—source data 1. [file elife-76157-fig7-data1.zip › Figure 7-source data 1/Figure 7B-row 2.tif]

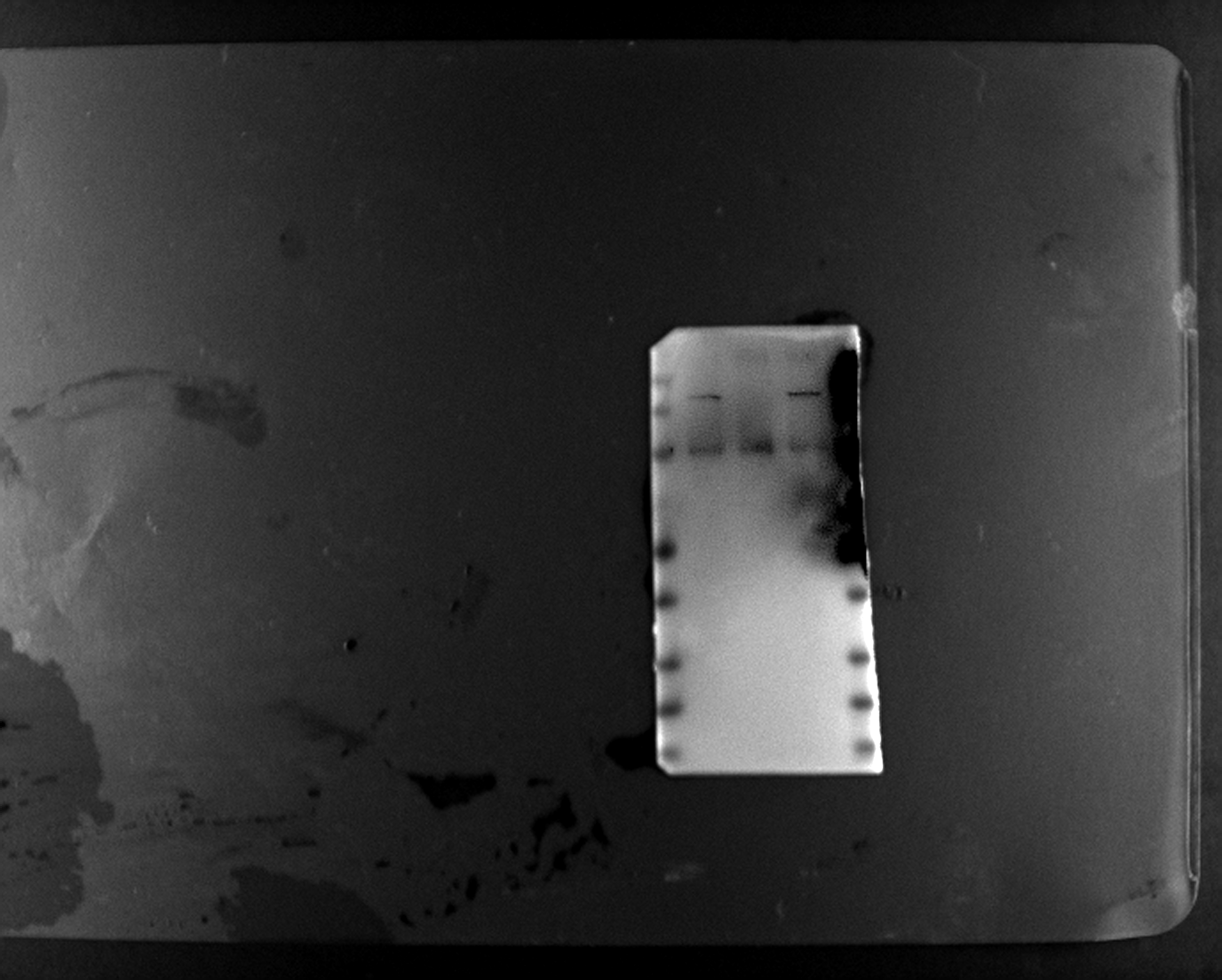

Supplement: Figure 7—source data 1. [file elife-76157-fig7-data1.zip › Figure 7-source data 1/Figure 7B-row 6.tif]

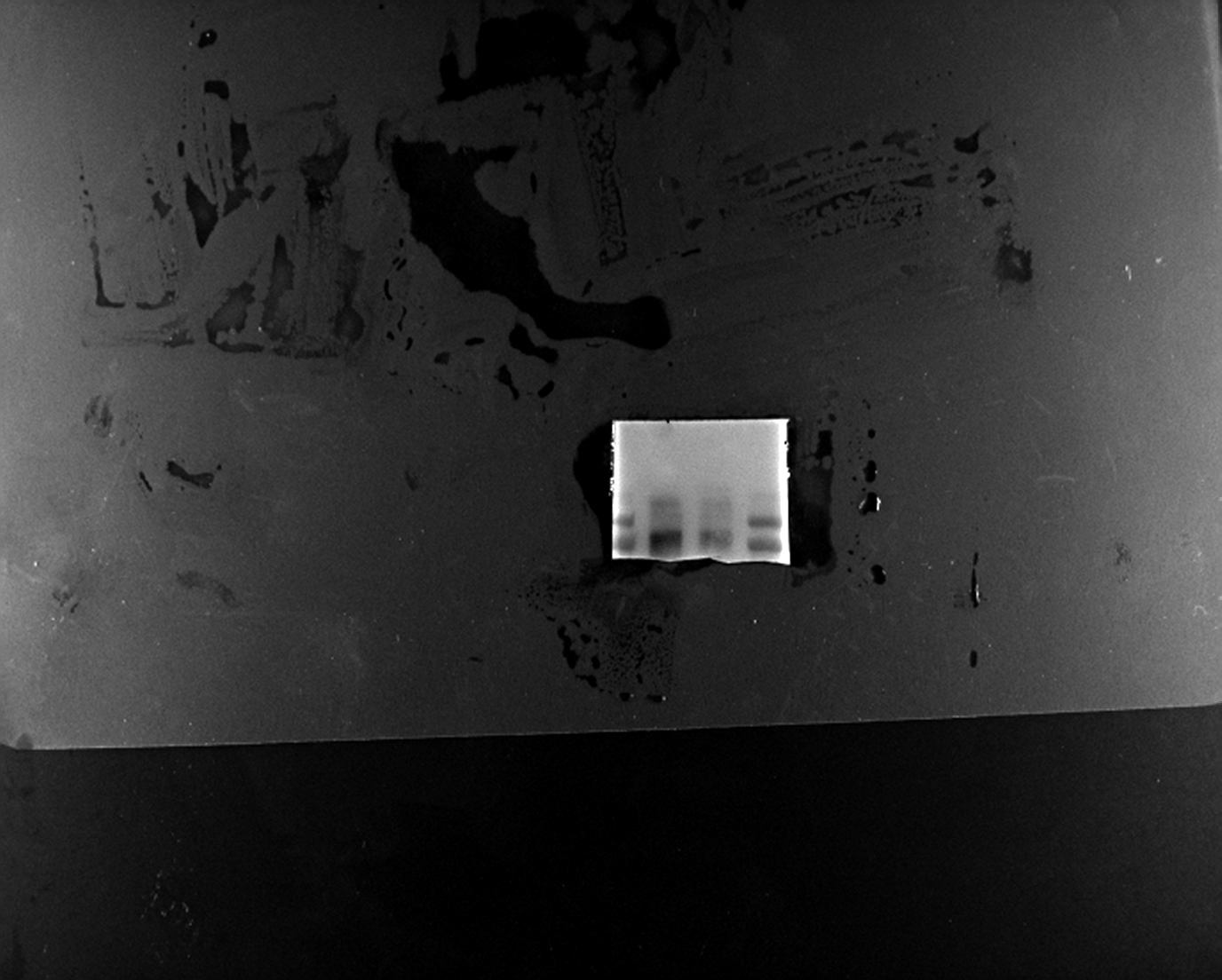

Supplement: Figure 7—source data 1. [file elife-76157-fig7-data1.zip › Figure 7-source data 1/Figure 7D-row 1.tif]

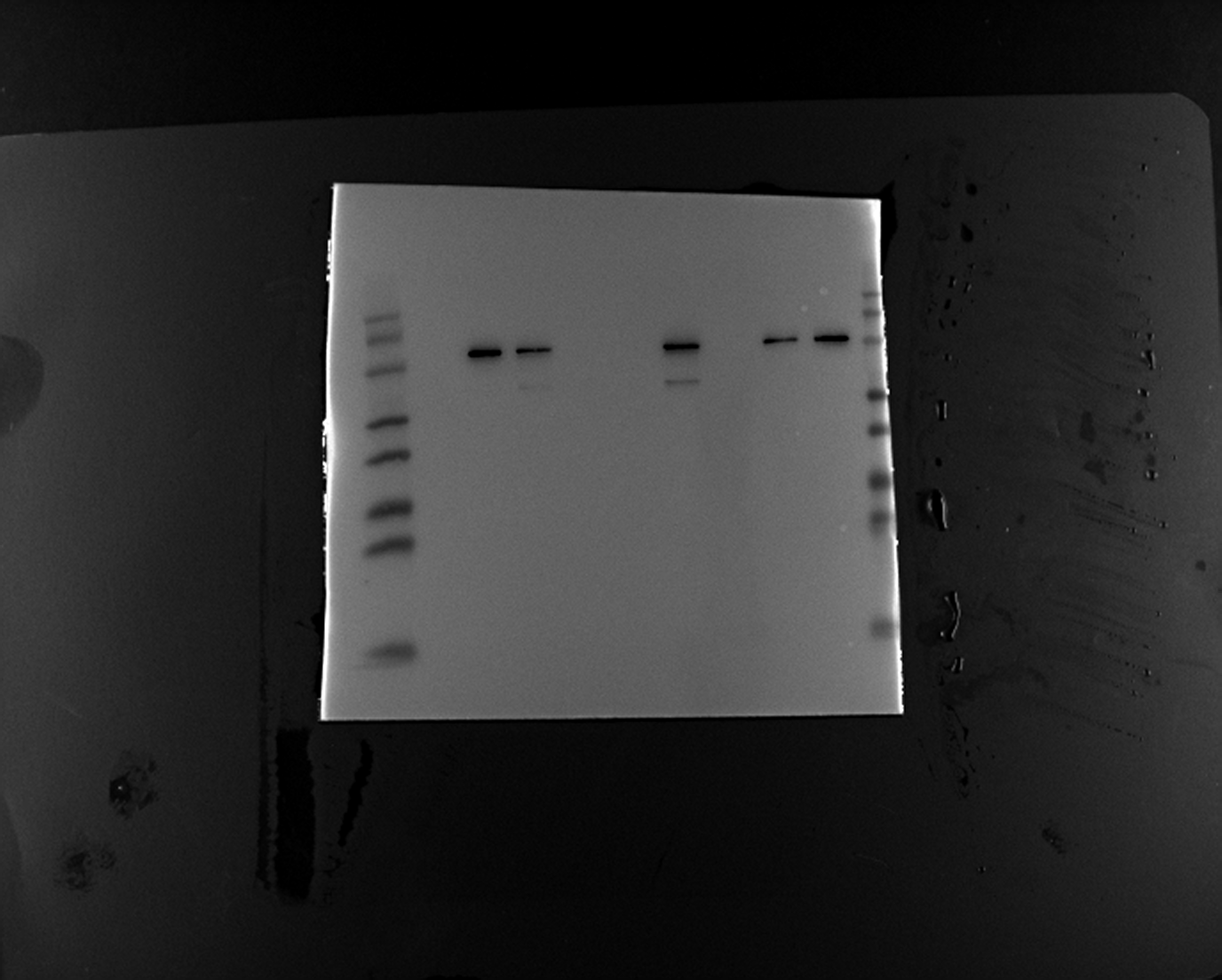

Supplement: Figure 7—source data 1. [file elife-76157-fig7-data1.zip › Figure 7-source data 1/Figure 7B-row 3 & row 5.tif]

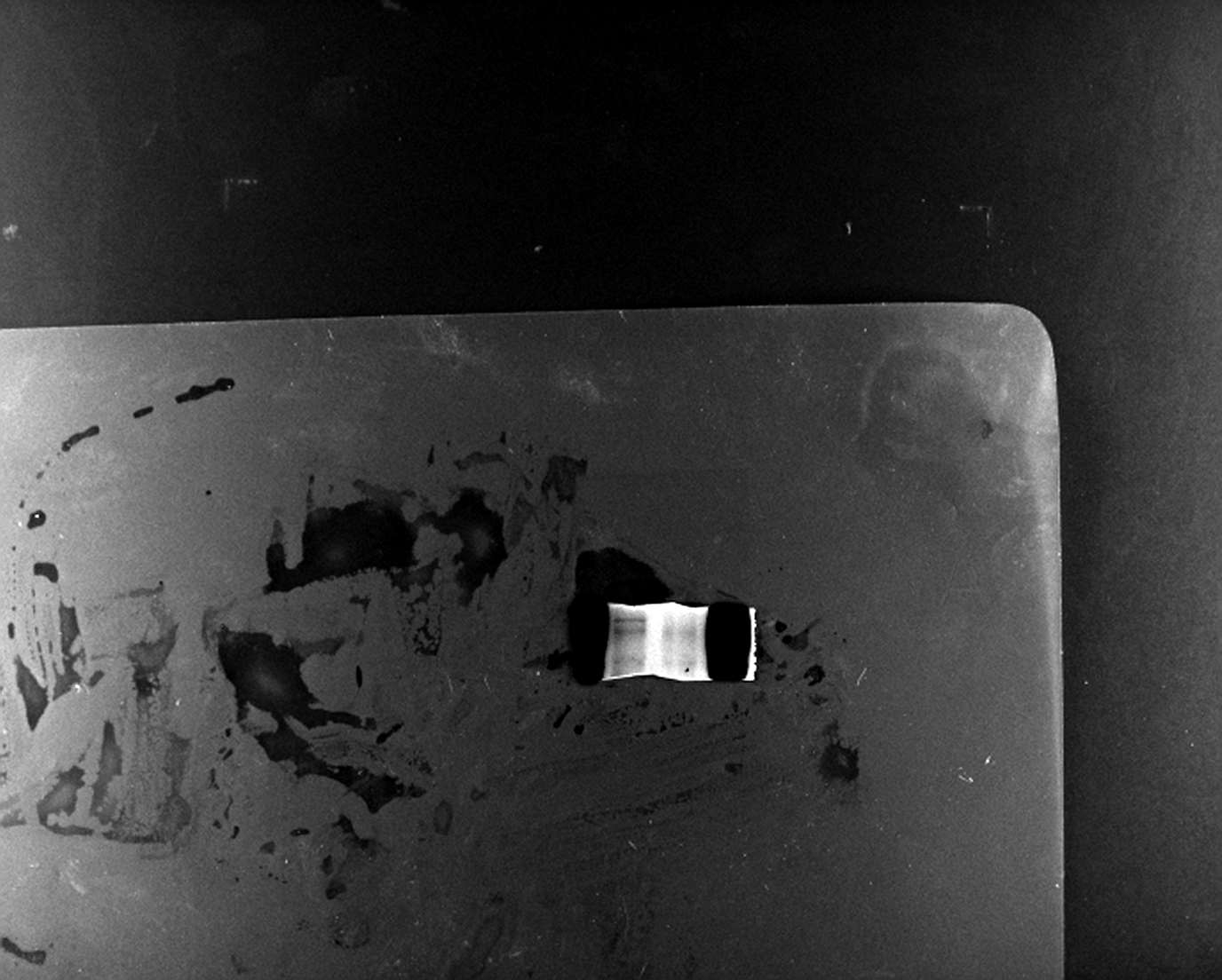

Supplement: Figure 7—source data 1. [file elife-76157-fig7-data1.zip › Figure 7-source data 1/Figure 7D-row 2.tif]

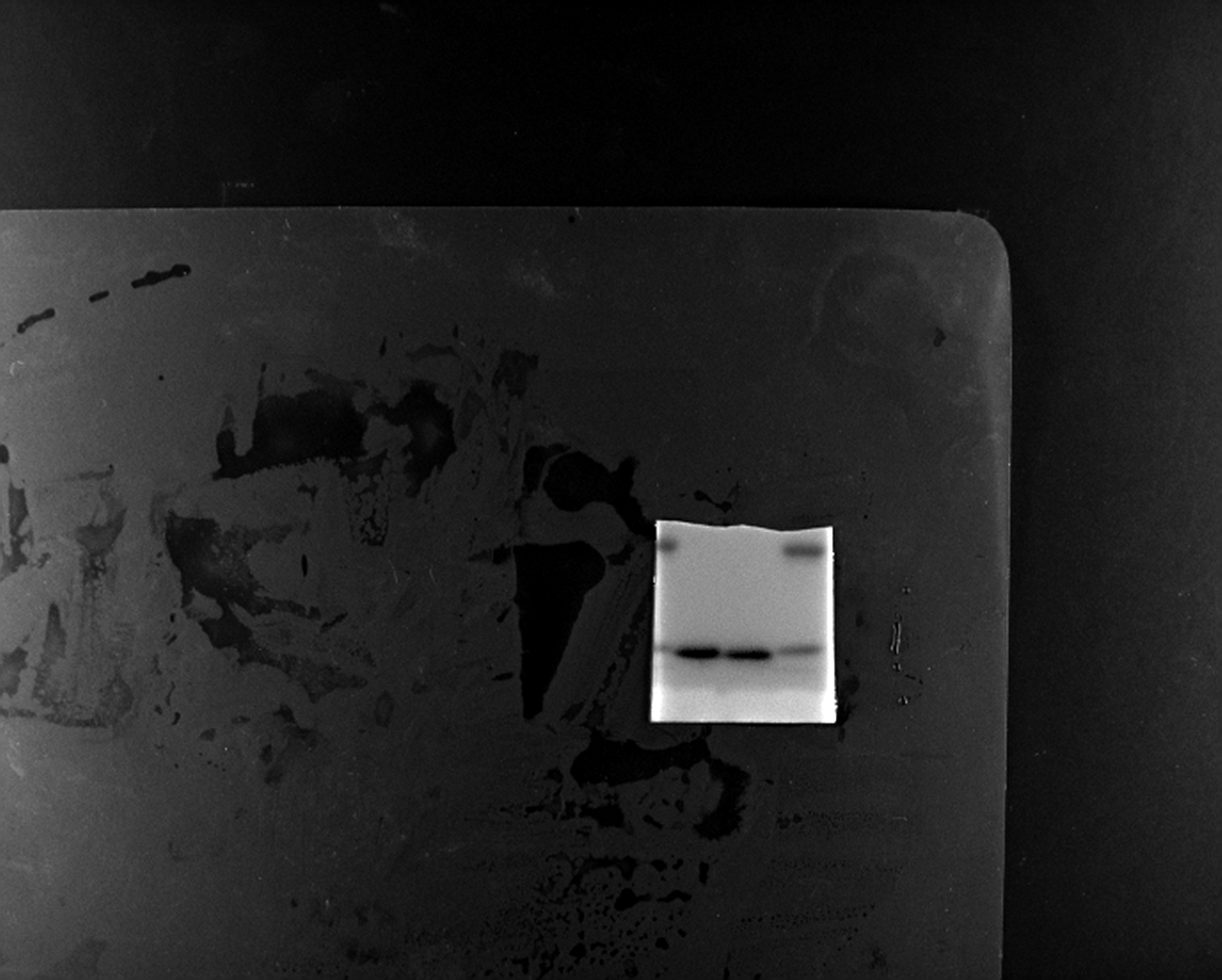

Supplement: Figure 7—source data 1. [file elife-76157-fig7-data1.zip › Figure 7-source data 1/Figure 7D-row 3.tif]

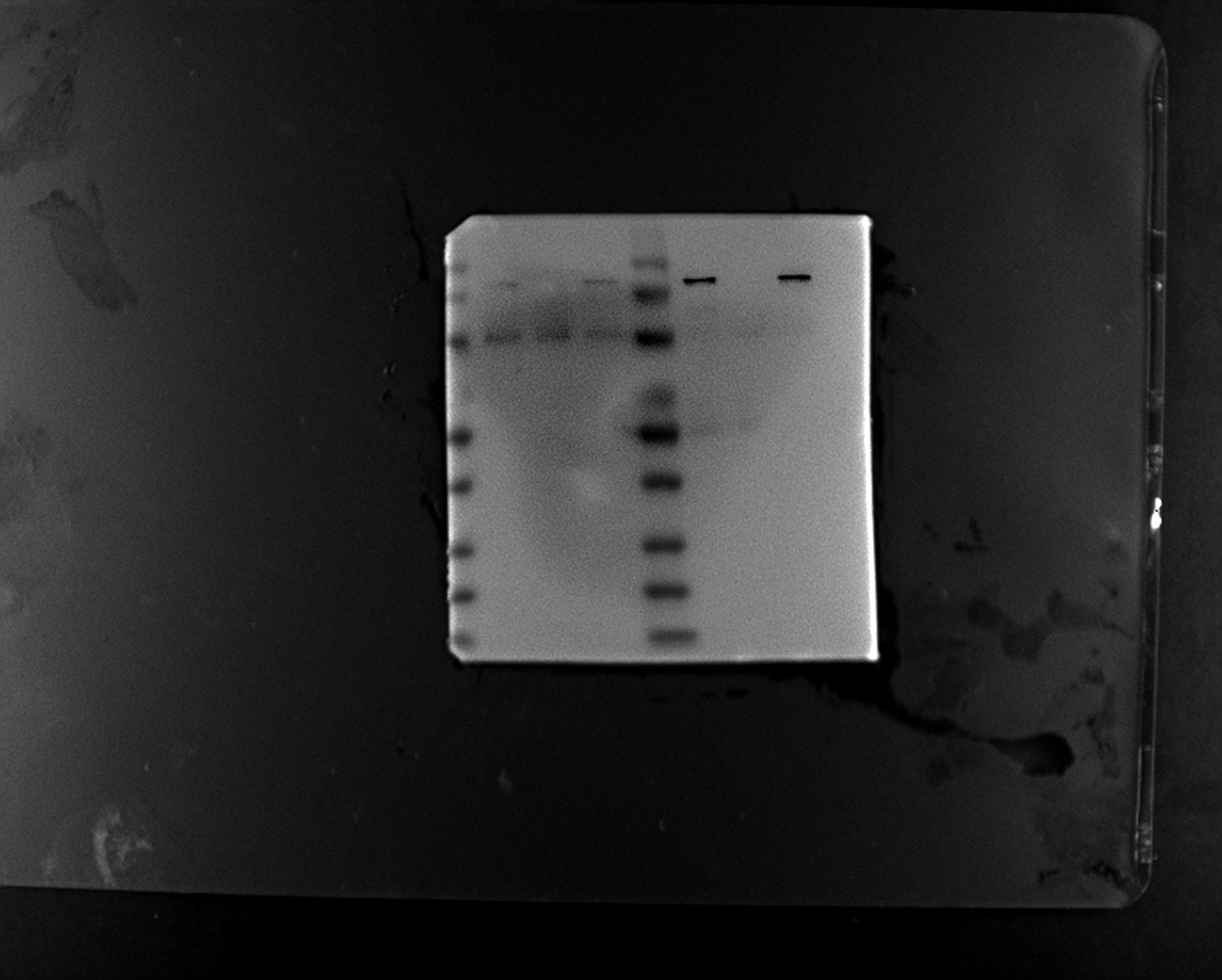

Supplement: Figure 7—source data 1. [file elife-76157-fig7-data1.zip › Figure 7-source data 1/Figure 7B-row 4.tif]

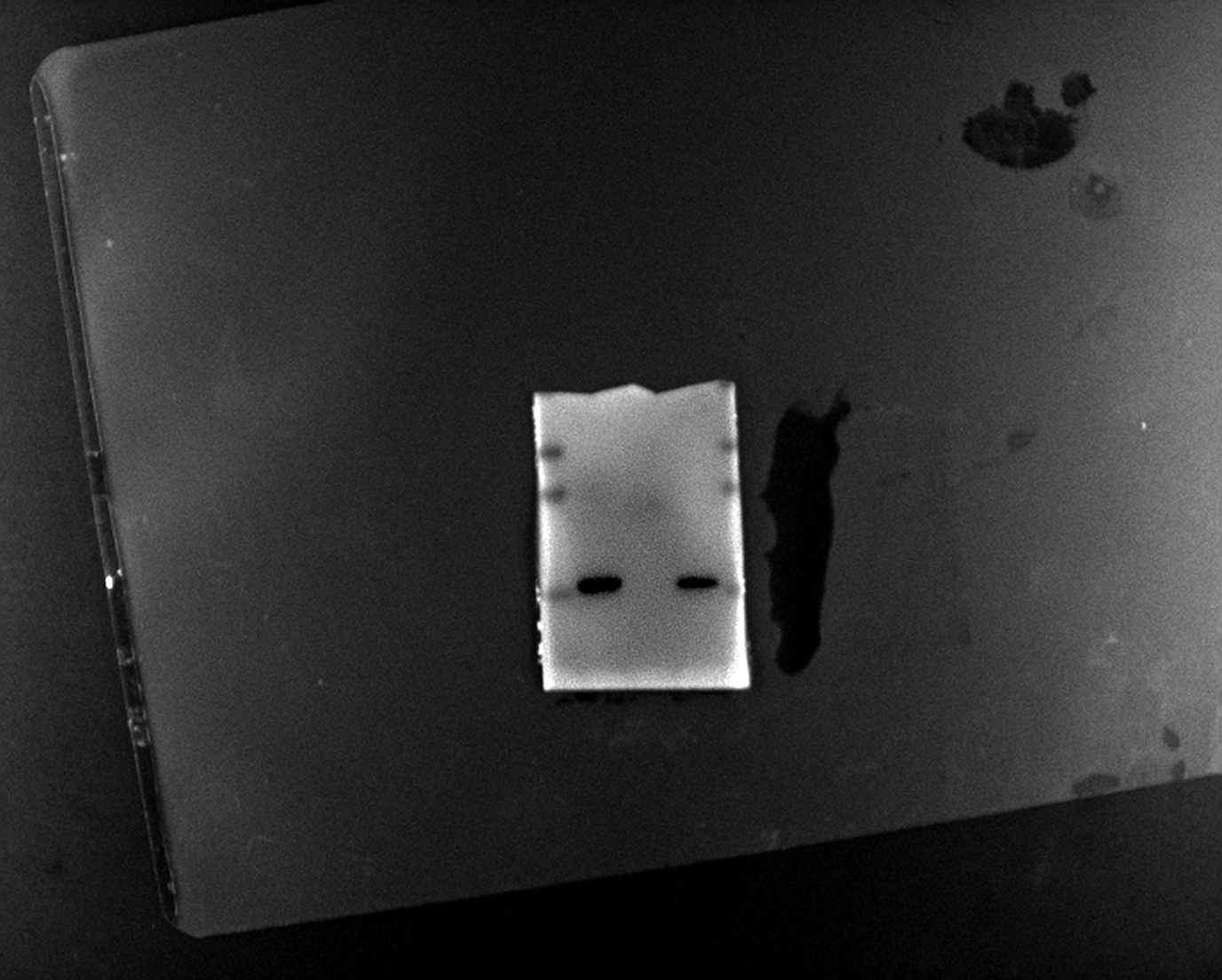

Supplement: Figure 7—source data 1. [file elife-76157-fig7-data1.zip › Figure 7-source data 1/Figure 7C-row 1.tif]

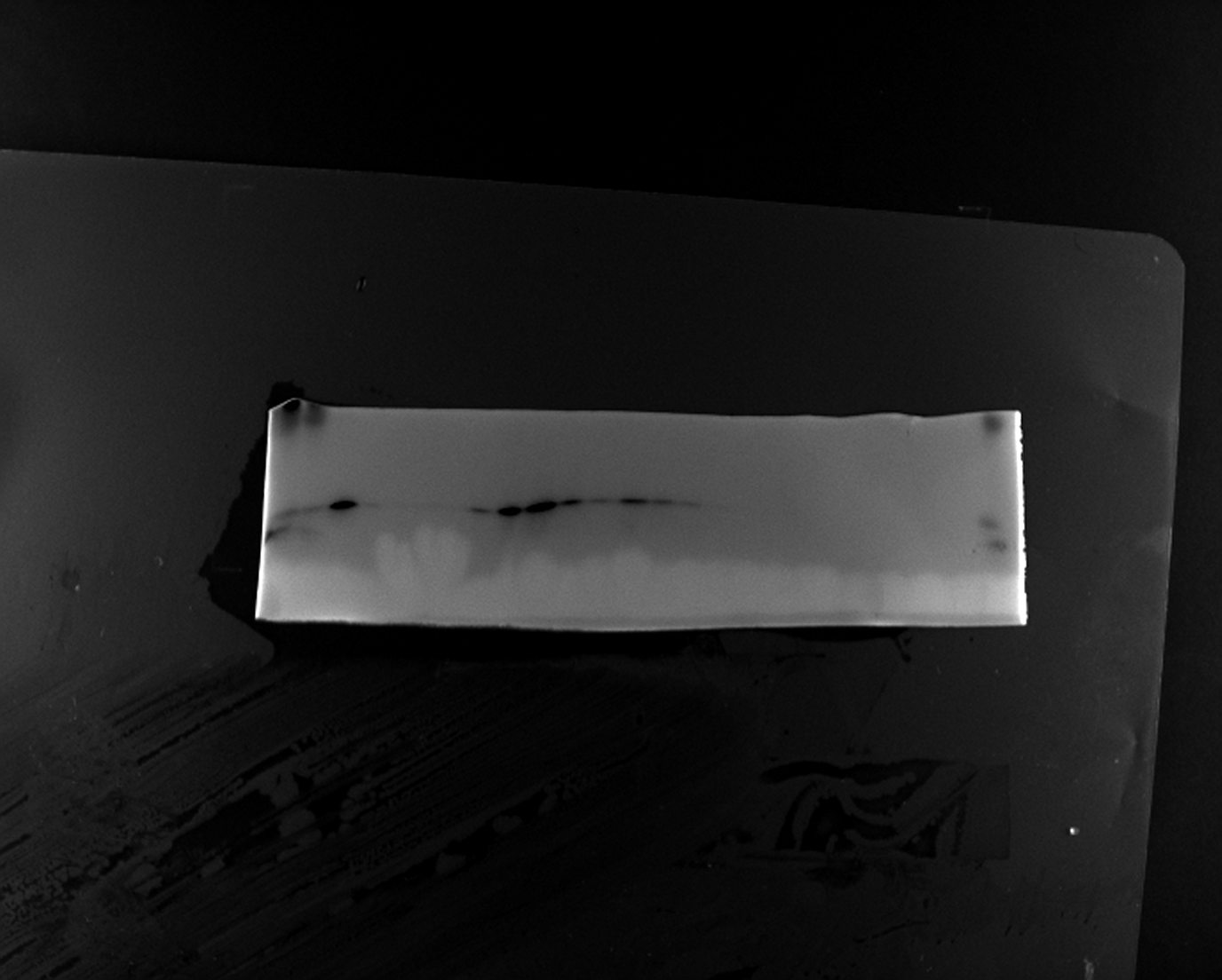

Supplement: Figure 7—source data 1. [file elife-76157-fig7-data1.zip › Figure 7-source data 1/Figure 7E-row 4.tif]

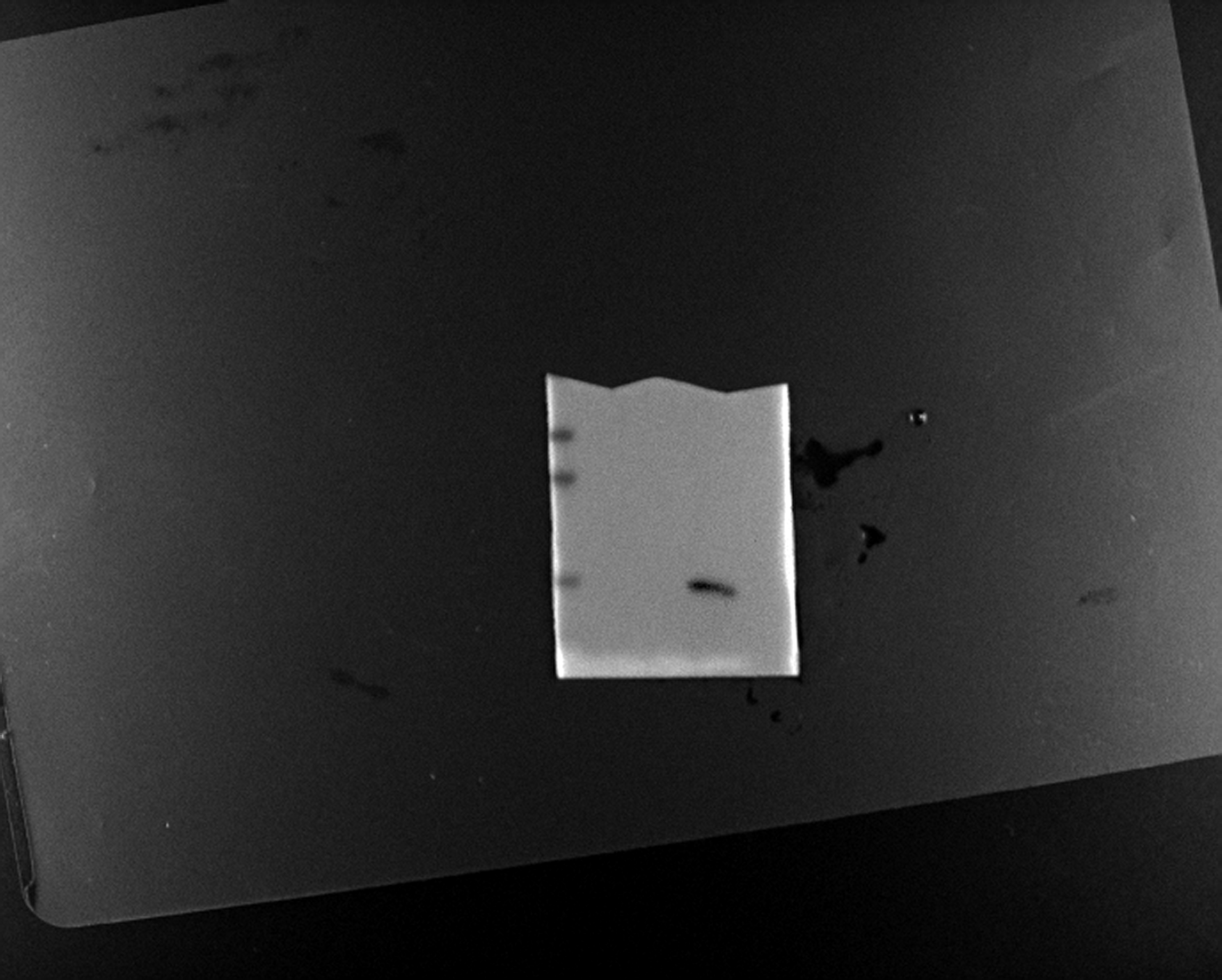

Supplement: Figure 7—source data 1. [file elife-76157-fig7-data1.zip › Figure 7-source data 1/Figure 7C-row 3.tif]

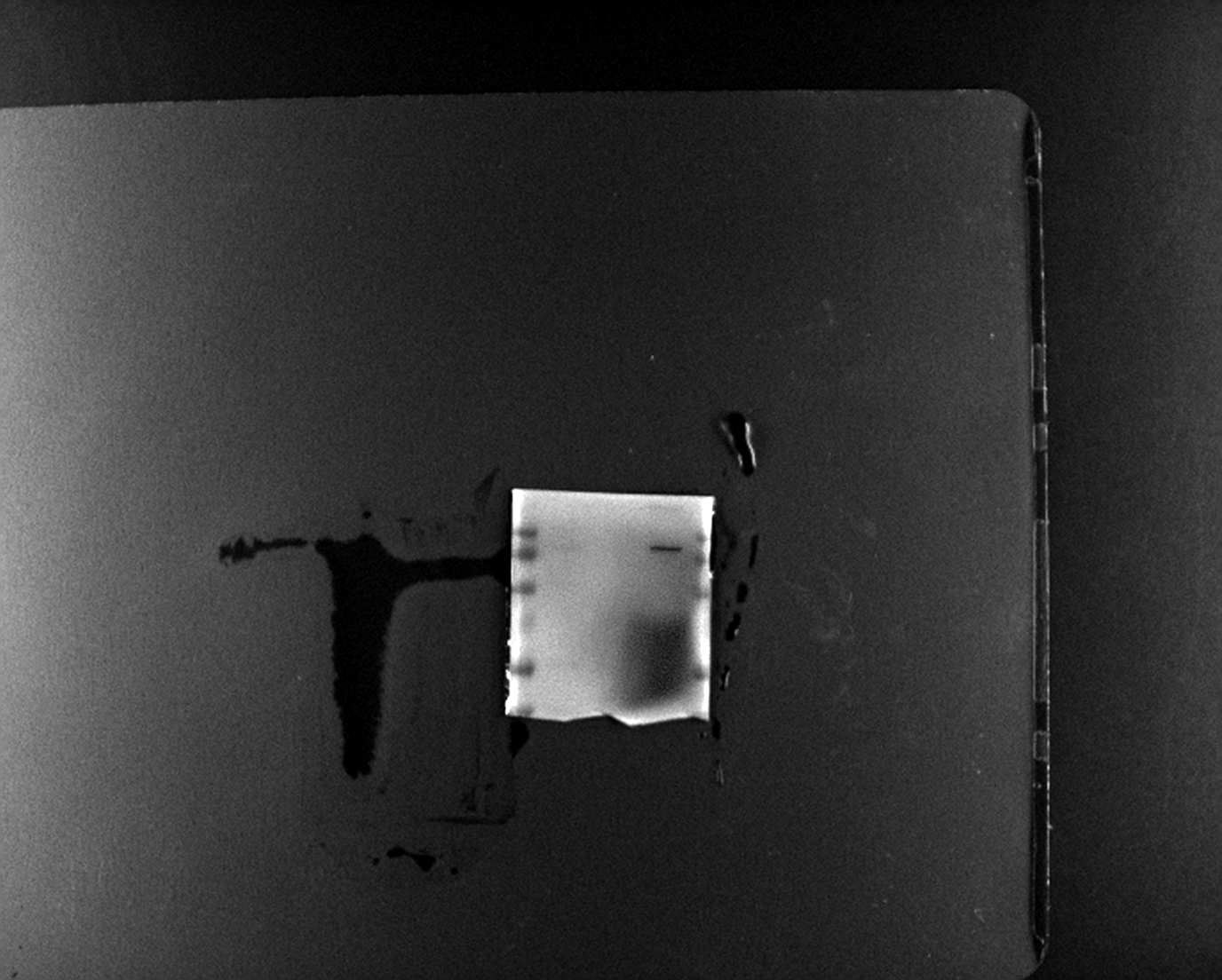

Supplement: Figure 7—source data 1. [file elife-76157-fig7-data1.zip › Figure 7-source data 1/Figure 7C-row 2.tif]

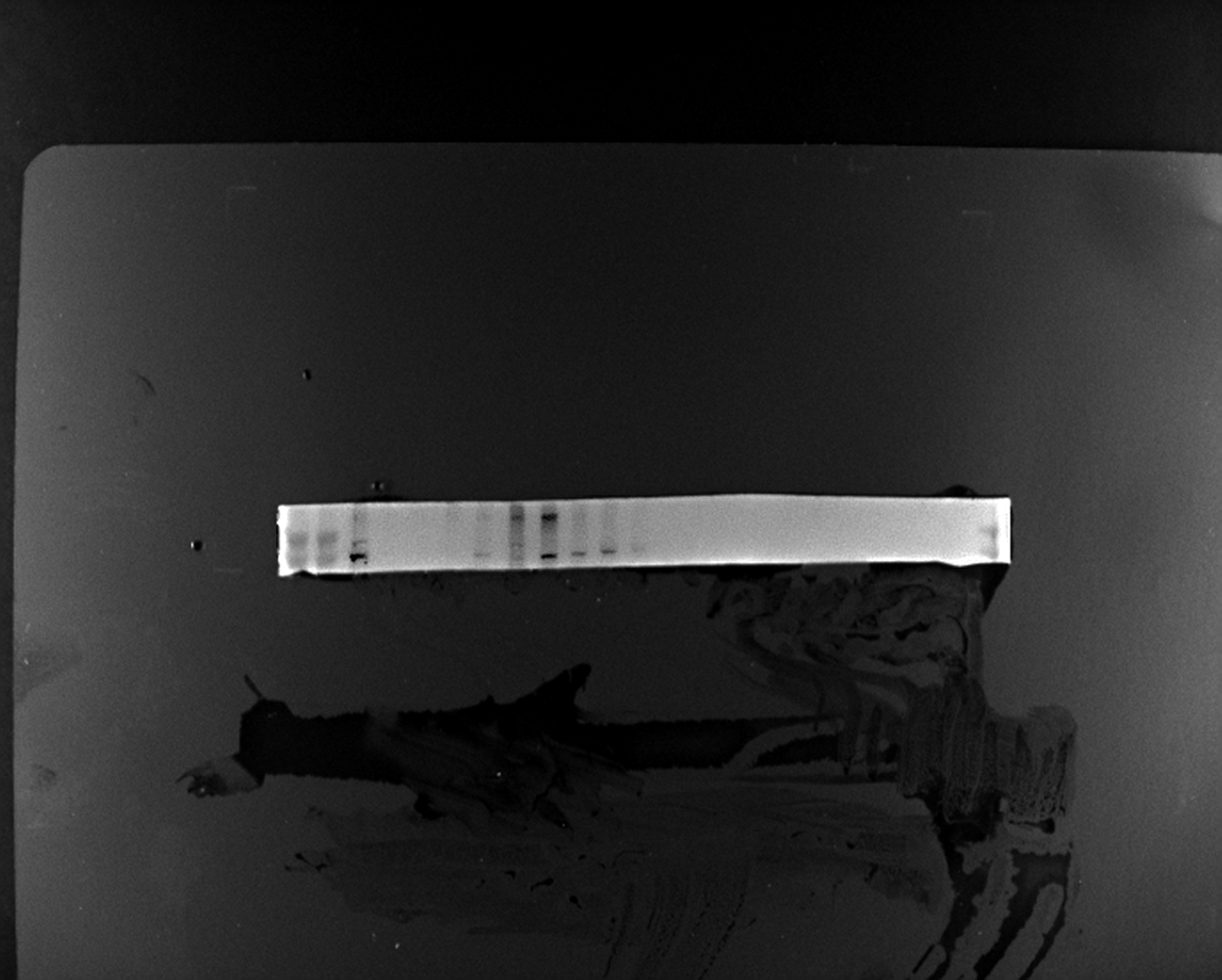

Supplement: Figure 7—source data 1. [file elife-76157-fig7-data1.zip › Figure 7-source data 1/Figure 7E-row 1.tif]

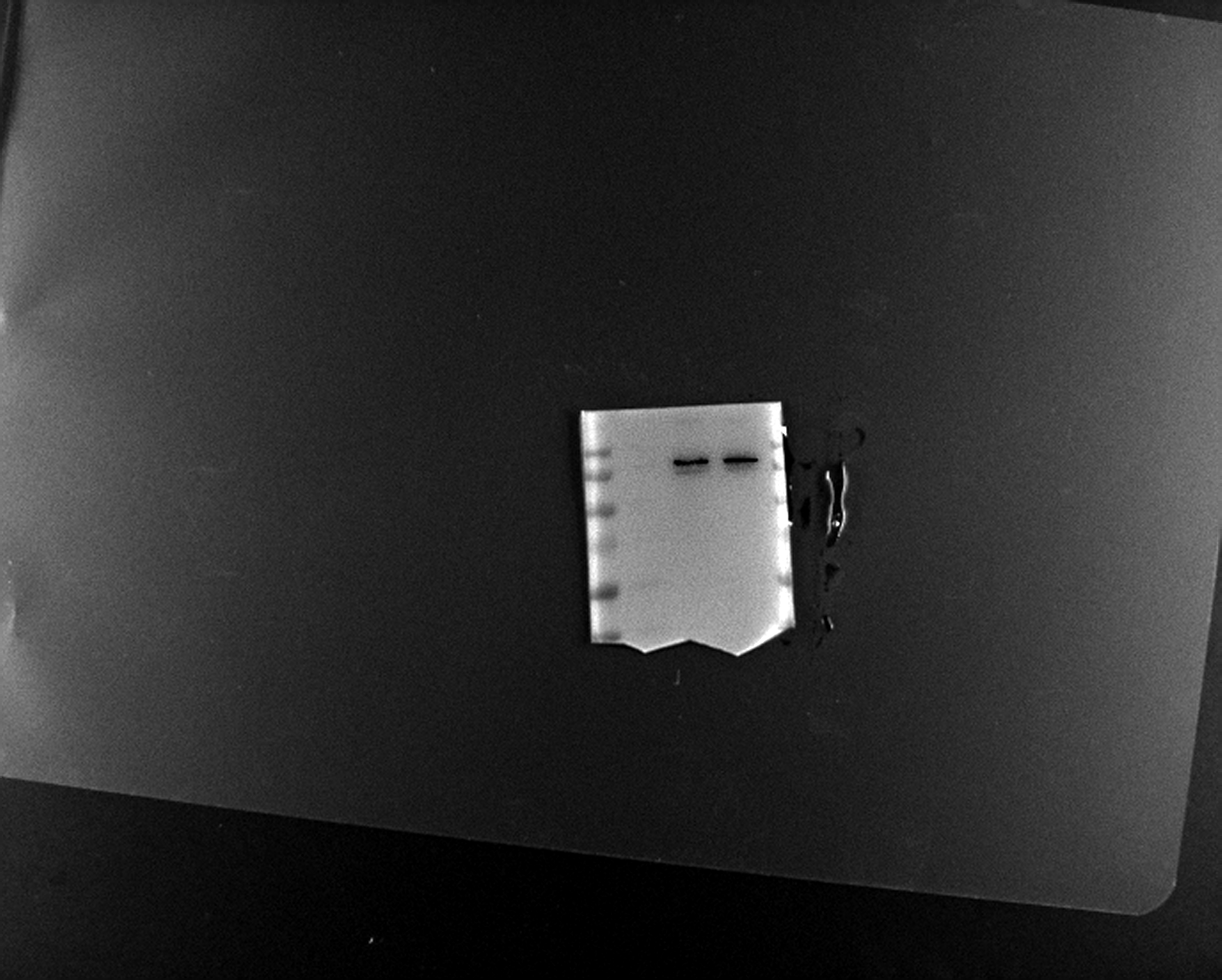

Supplement: Figure 7—source data 1. [file elife-76157-fig7-data1.zip › Figure 7-source data 1/Figure 7C-row 6.tif]

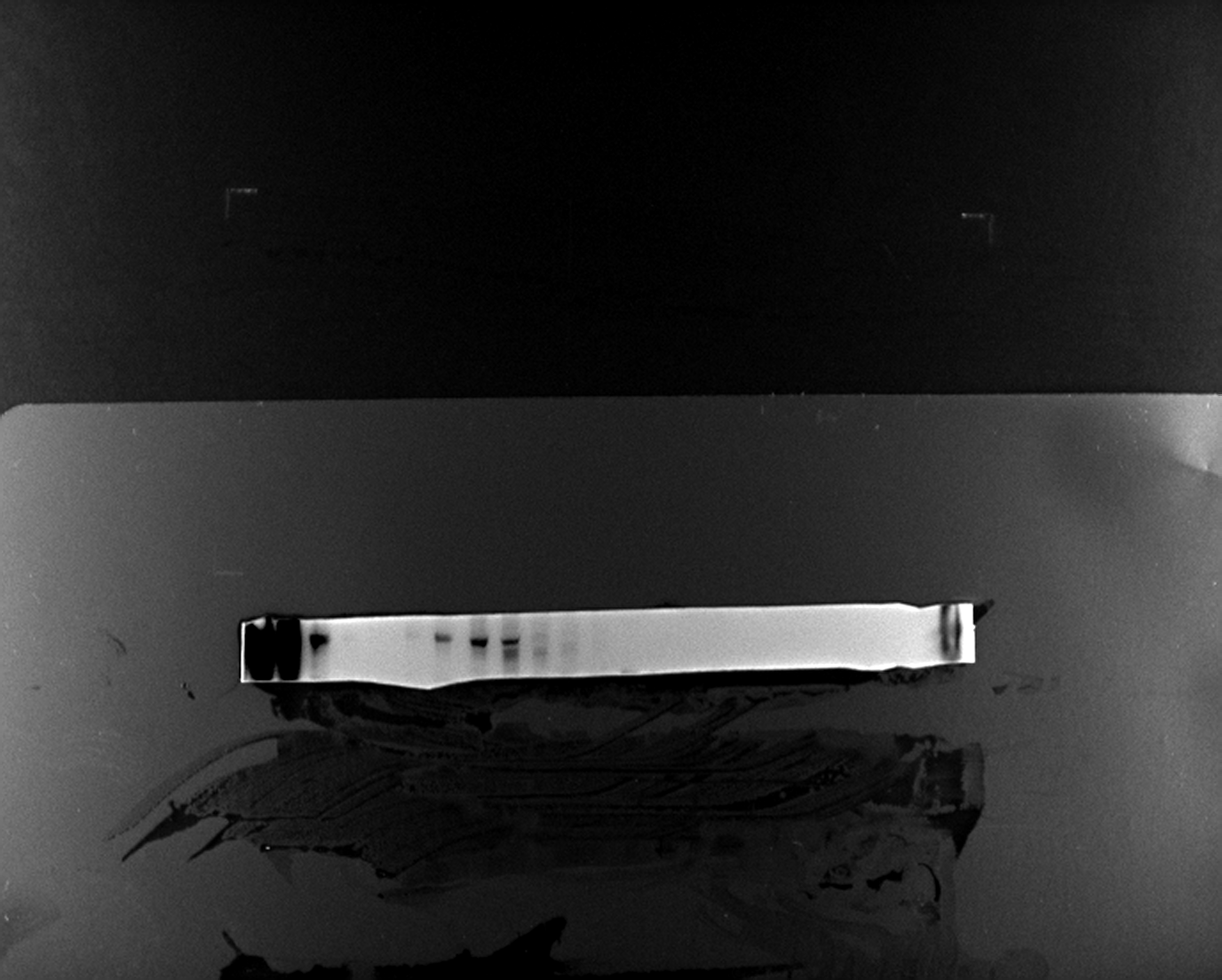

Supplement: Figure 7—source data 1. [file elife-76157-fig7-data1.zip › Figure 7-source data 1/Figure 7E-row 2.tif]

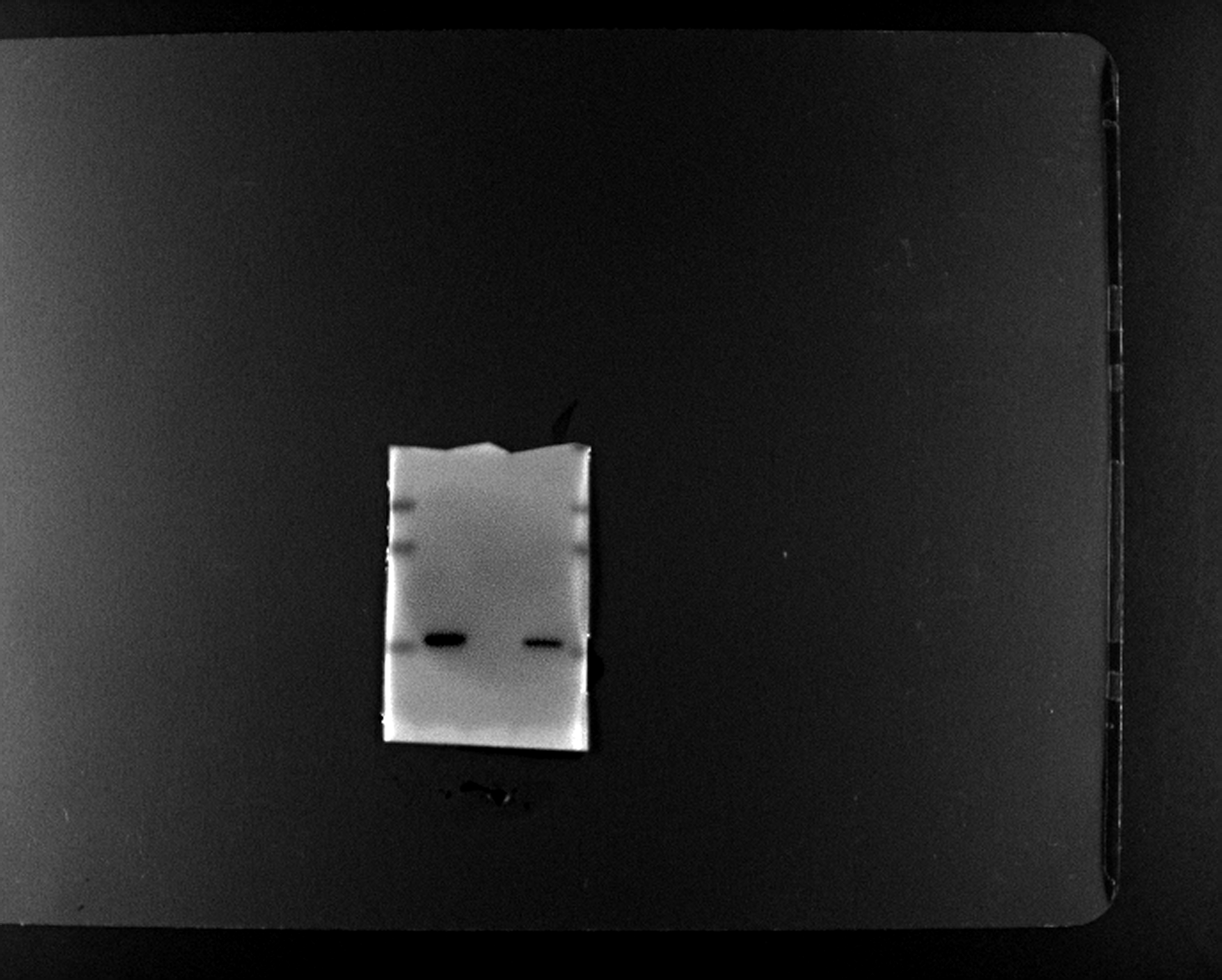

Supplement: Figure 7—source data 1. [file elife-76157-fig7-data1.zip › Figure 7-source data 1/Figure 7C-row 5.tif]

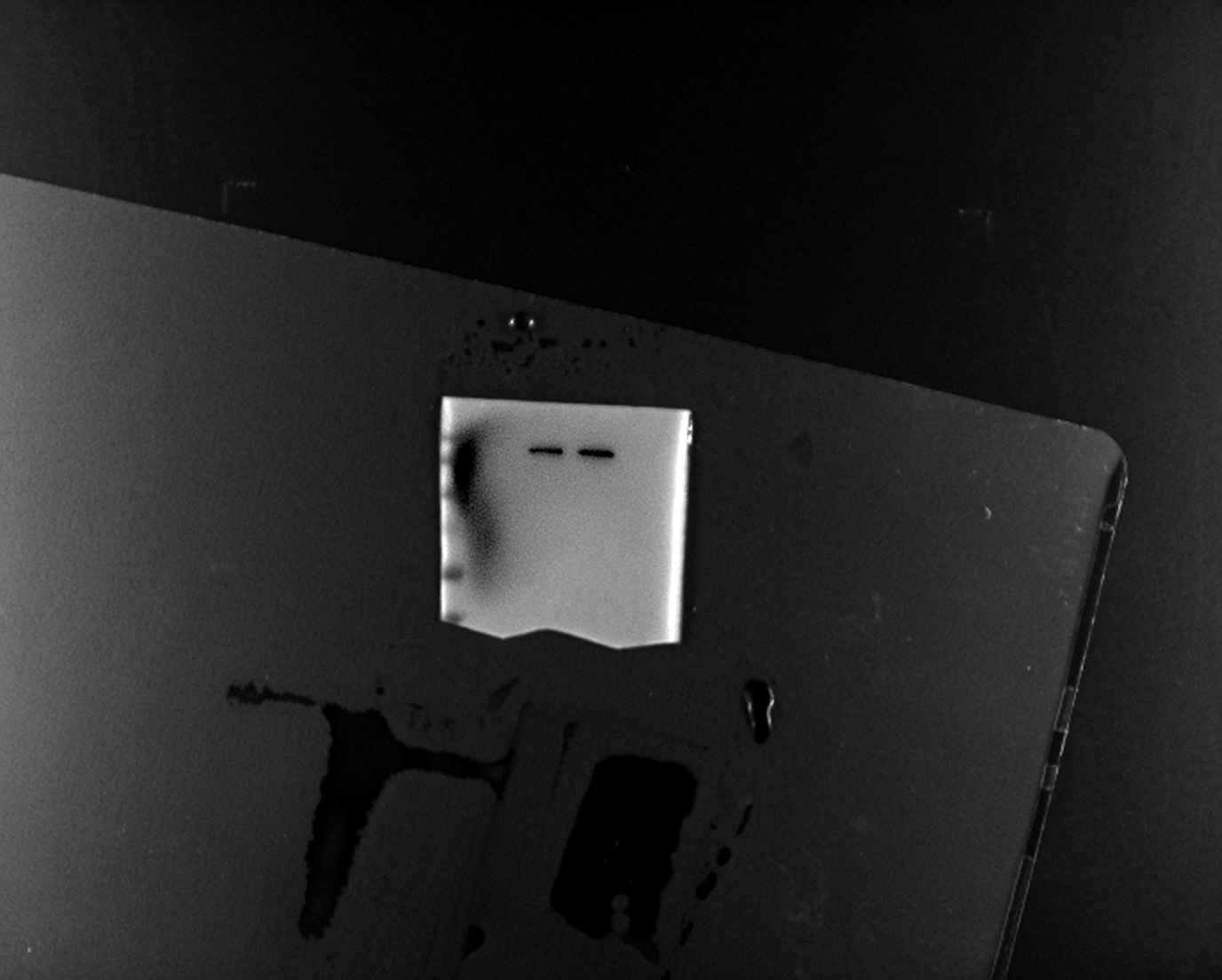

Supplement: Figure 7—source data 1. [file elife-76157-fig7-data1.zip › Figure 7-source data 1/Figure 7C-row 4.tif]

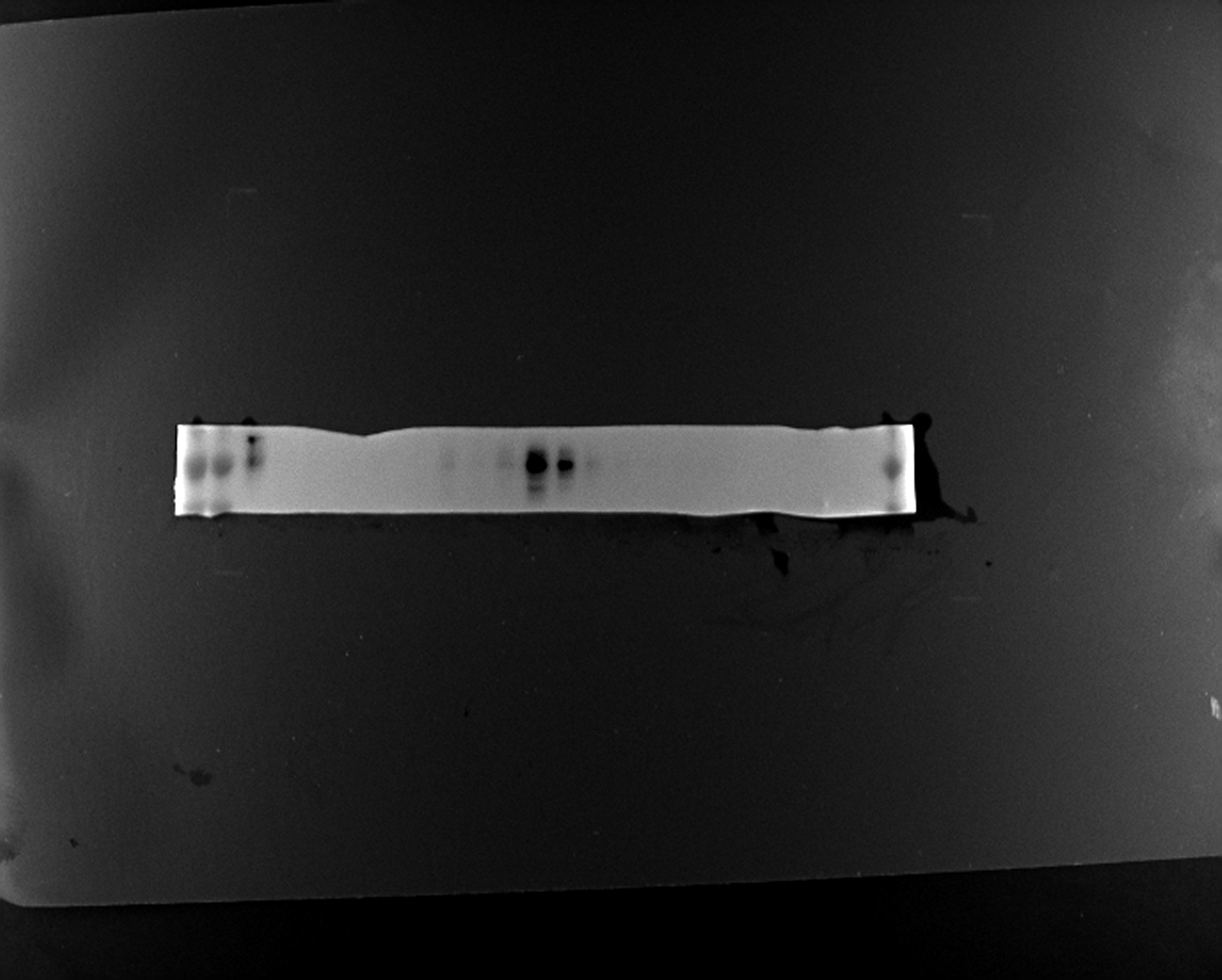

Supplement: Figure 7—source data 1. [file elife-76157-fig7-data1.zip › Figure 7-source data 1/Figure 7E-row 3.tif]

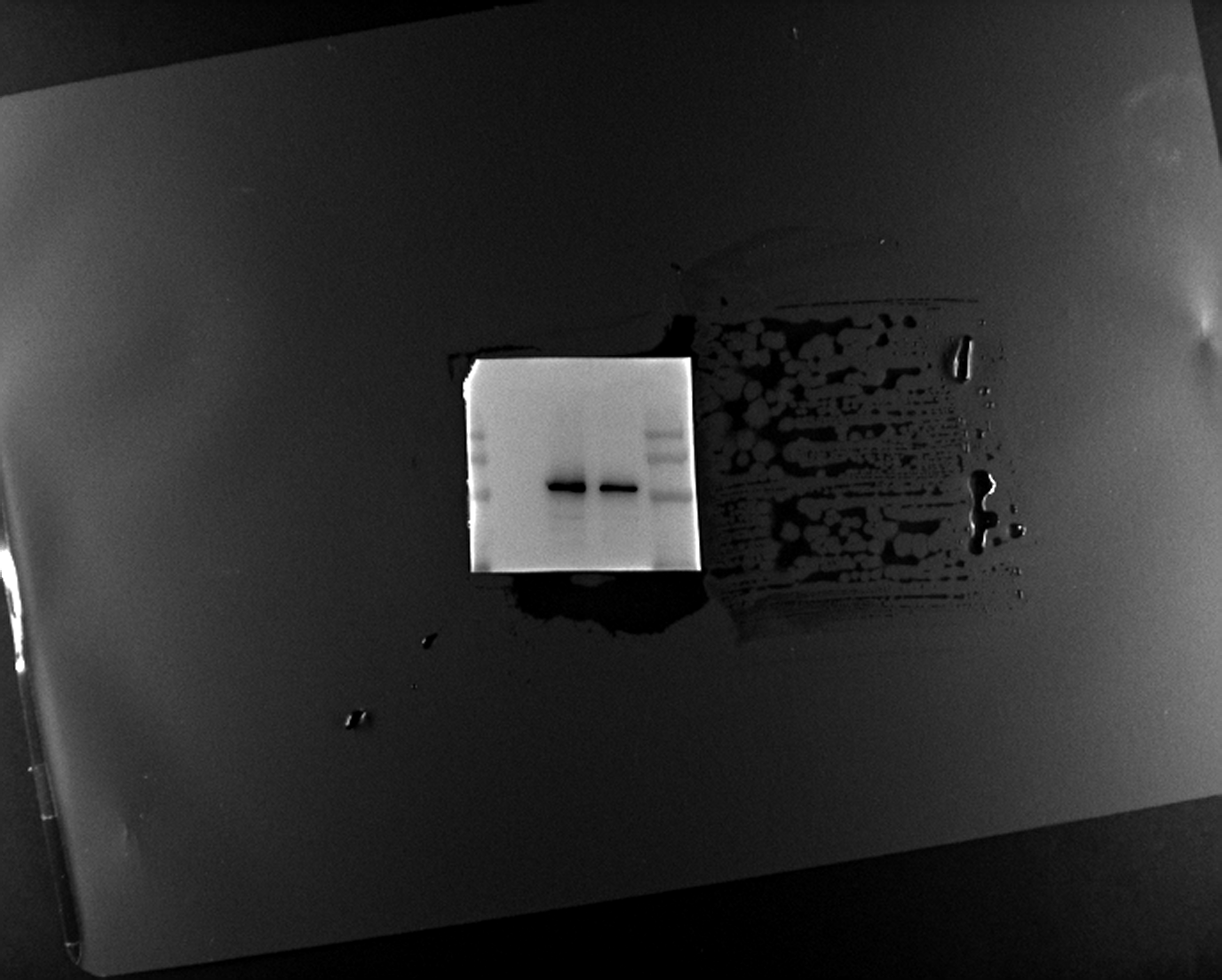

Supplement: Figure 7—source data 1. [file elife-76157-fig7-data1.zip › Figure 7-source data 1/Figure 7A-row 1.tif]

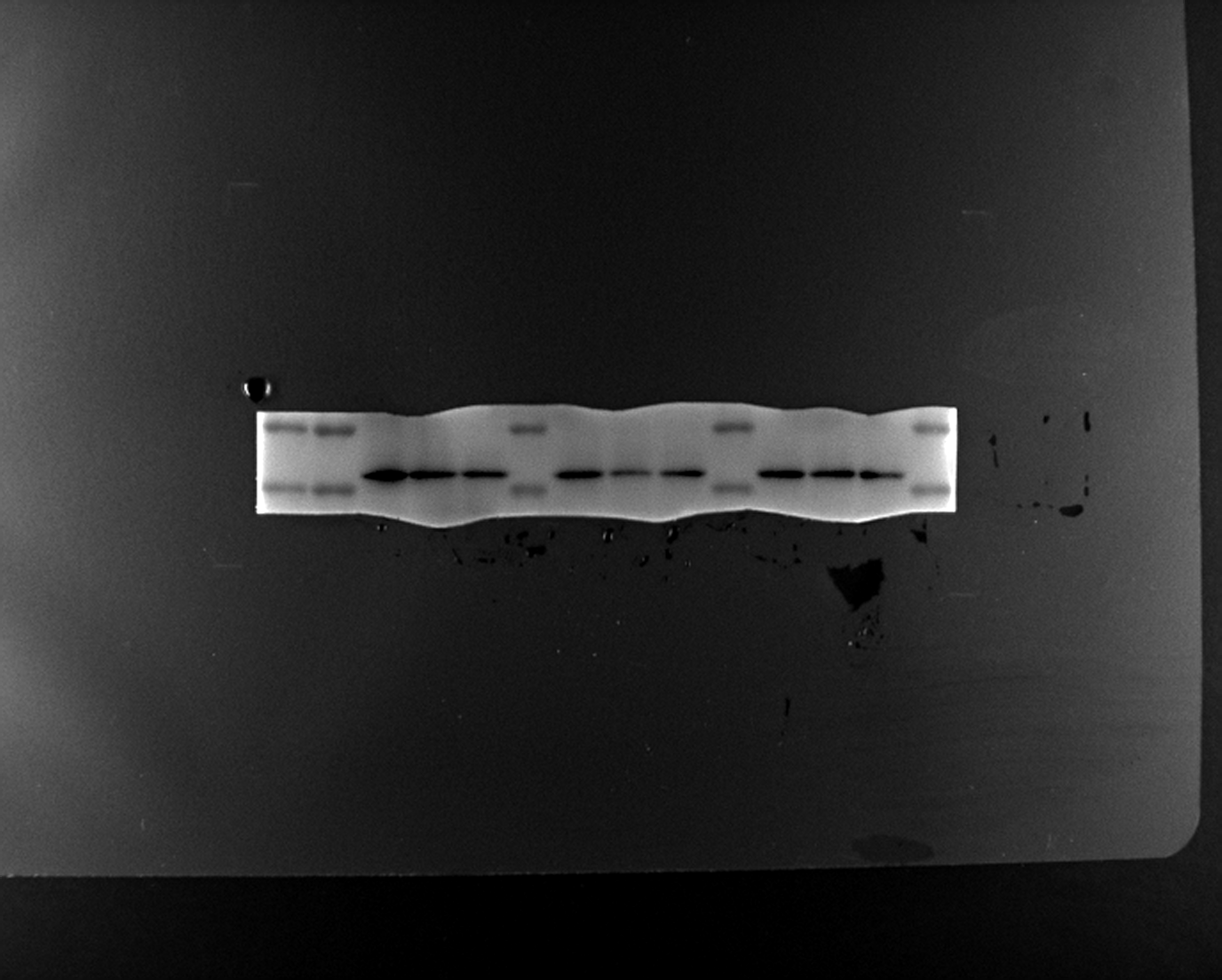

Supplement: Figure 7—source data 1. [file elife-76157-fig7-data1.zip › Figure 7-source data 1/Figure 7J-row 5.tif]

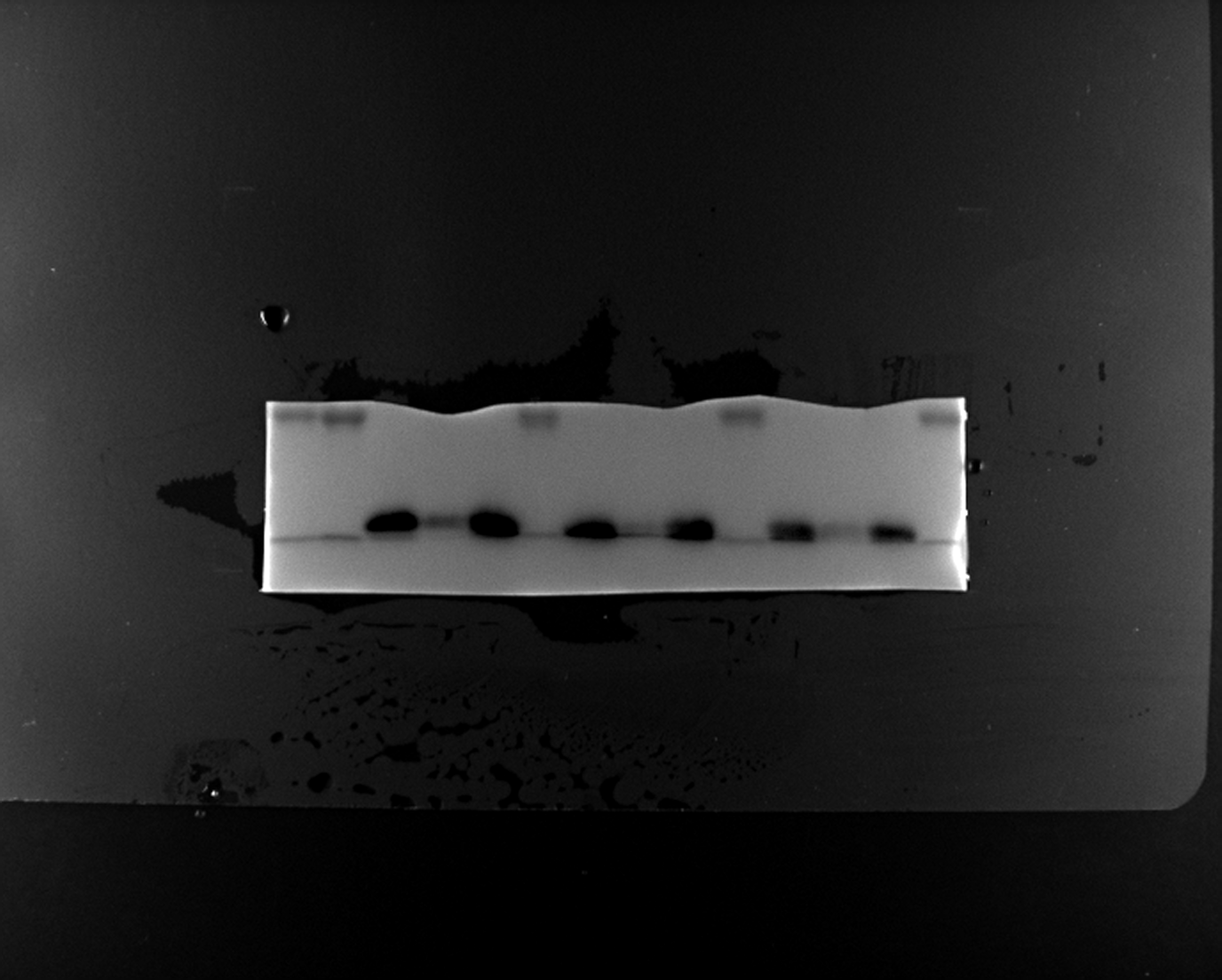

Supplement: Figure 7—source data 1. [file elife-76157-fig7-data1.zip › Figure 7-source data 1/Figure 7J-row 4.tif]

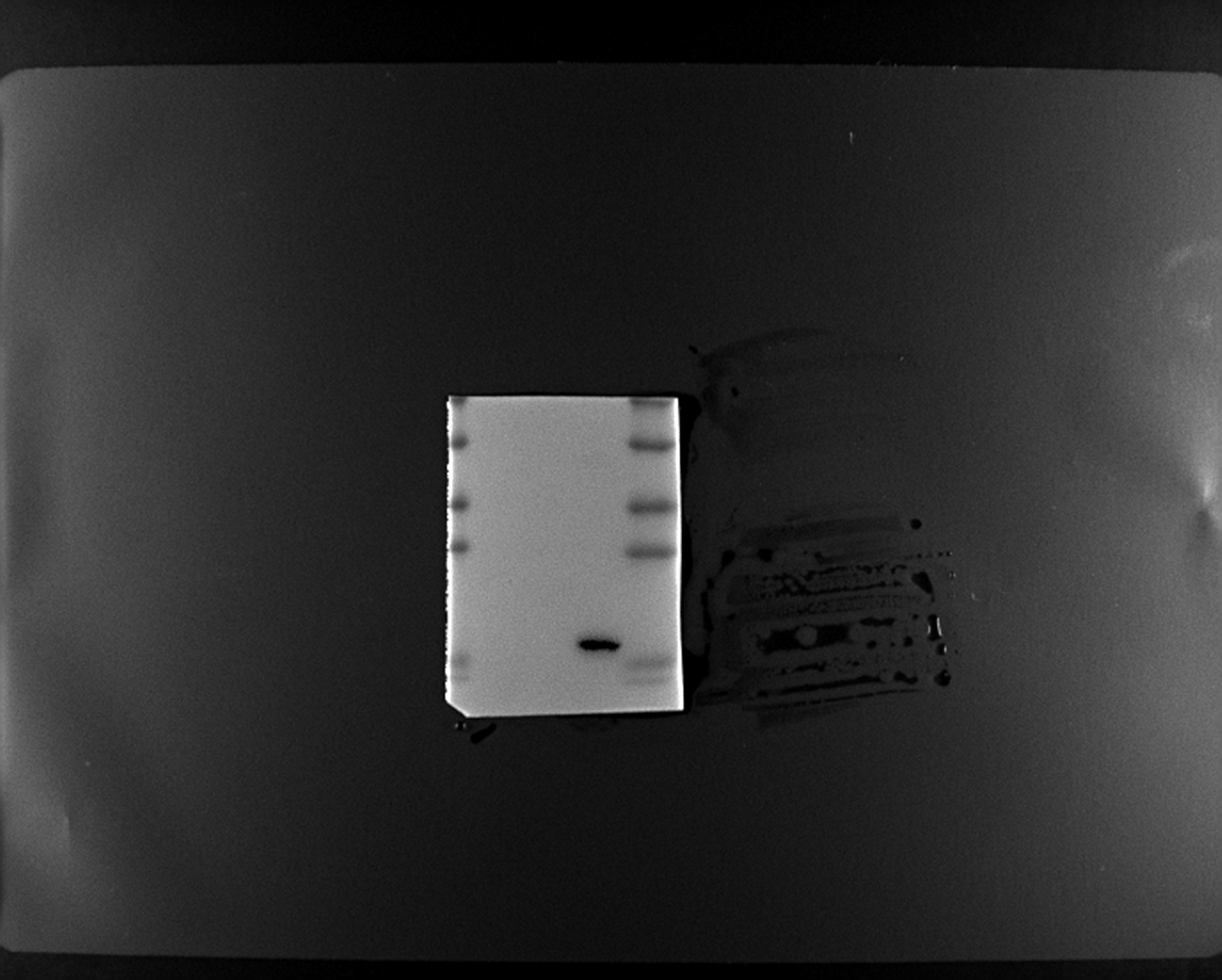

Supplement: Figure 7—source data 1. [file elife-76157-fig7-data1.zip › Figure 7-source data 1/Figure 7A-row 2.tif]

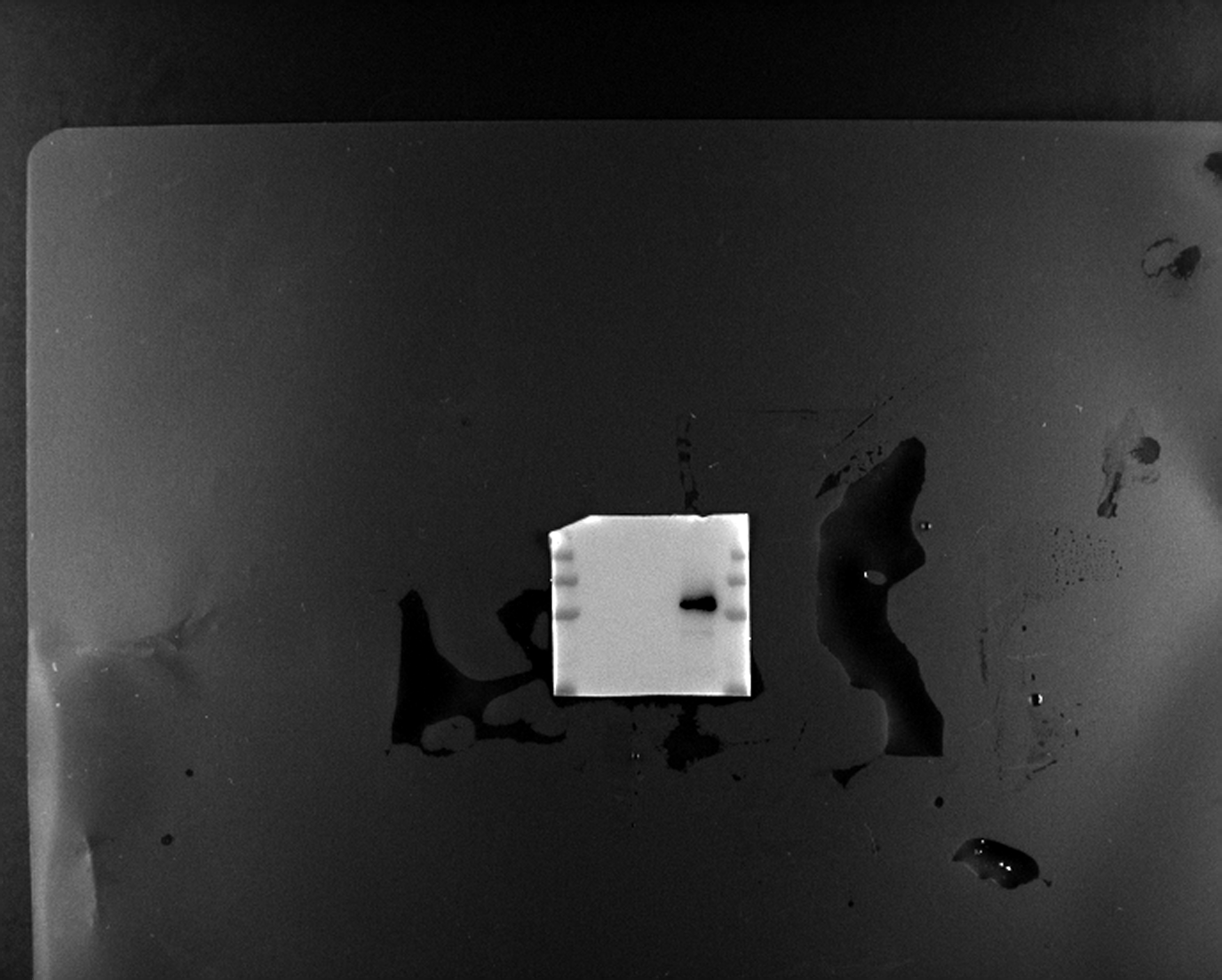

Supplement: Figure 7—source data 1. [file elife-76157-fig7-data1.zip › Figure 7-source data 1/Figure 7A-row 3.tif]

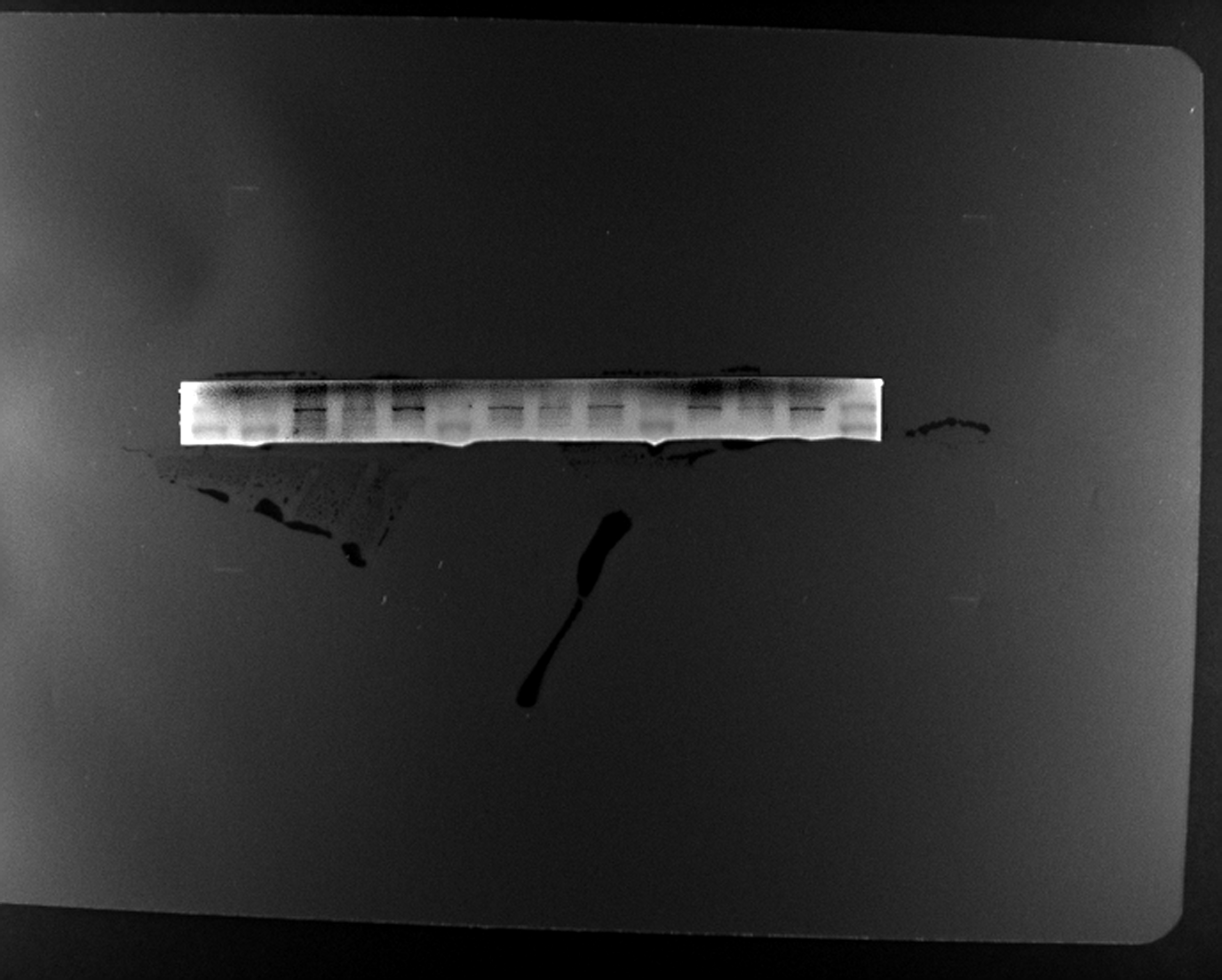

Supplement: Figure 7—source data 1. [file elife-76157-fig7-data1.zip › Figure 7-source data 1/Figure 7J-row 3.tif]

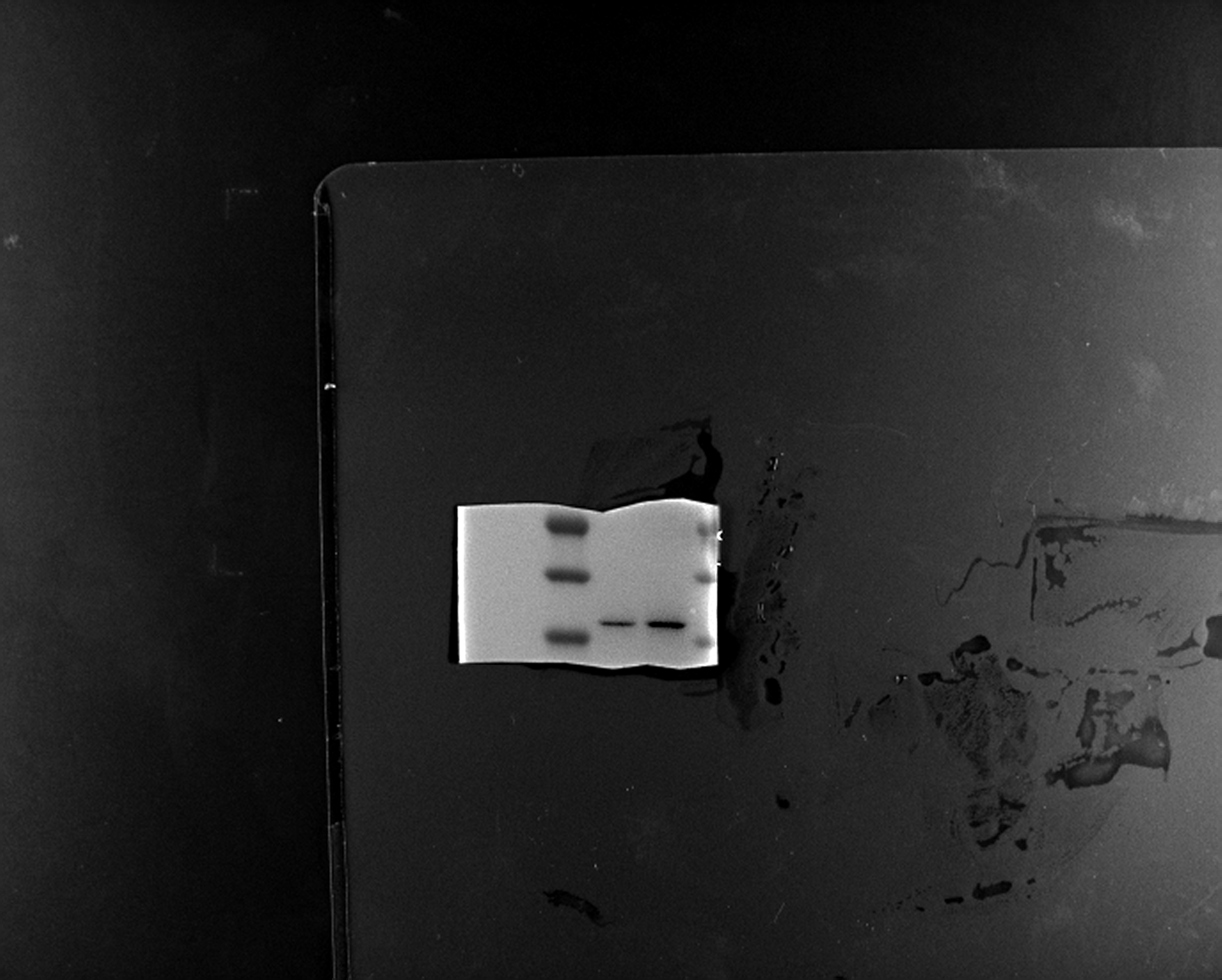

Supplement: Figure 7—source data 1. [file elife-76157-fig7-data1.zip › Figure 7-source data 1/Figure 7D-row 8.tif]

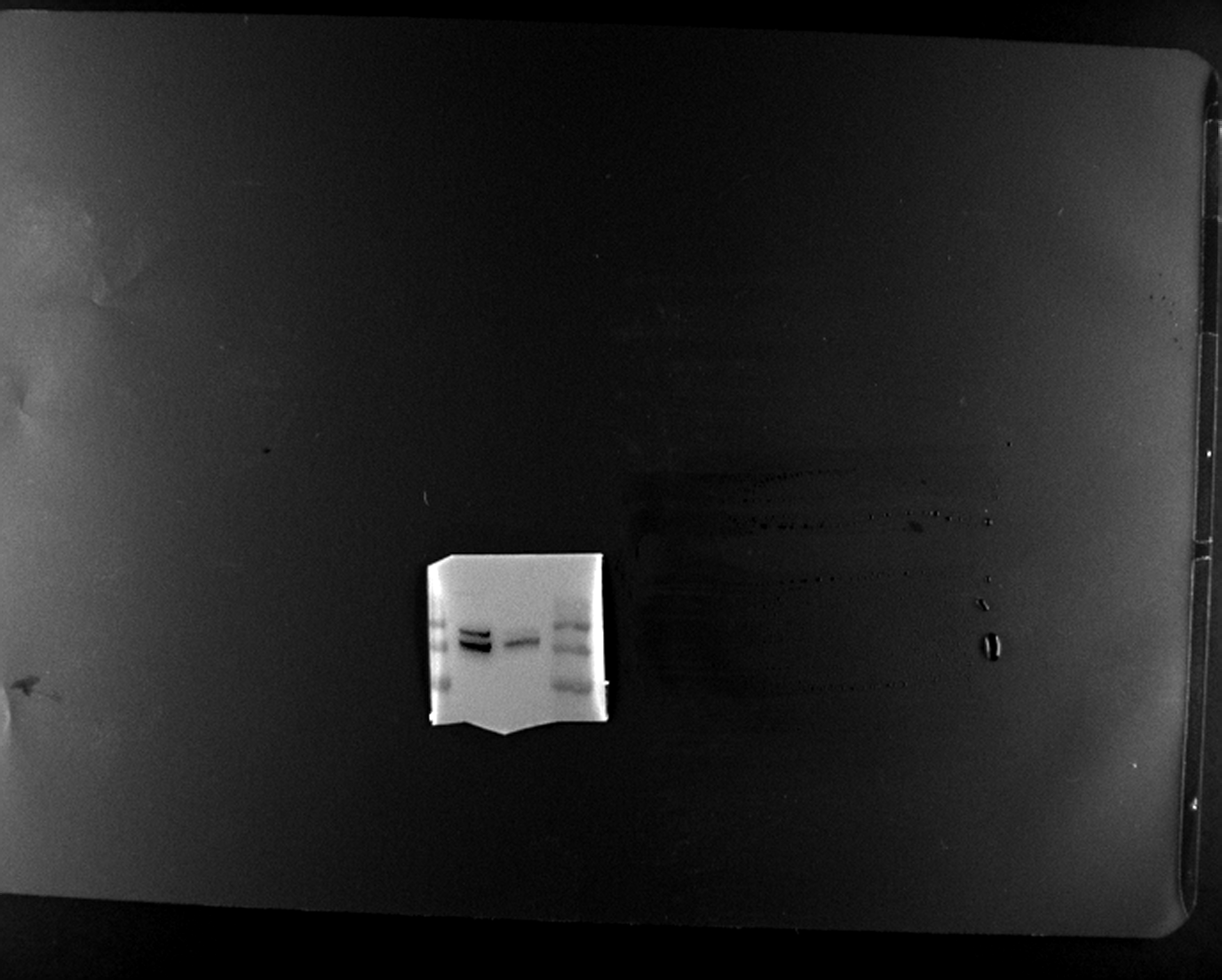

Supplement: Figure 7—source data 1. [file elife-76157-fig7-data1.zip › Figure 7-source data 1/Figure 7G-row 1.tif]

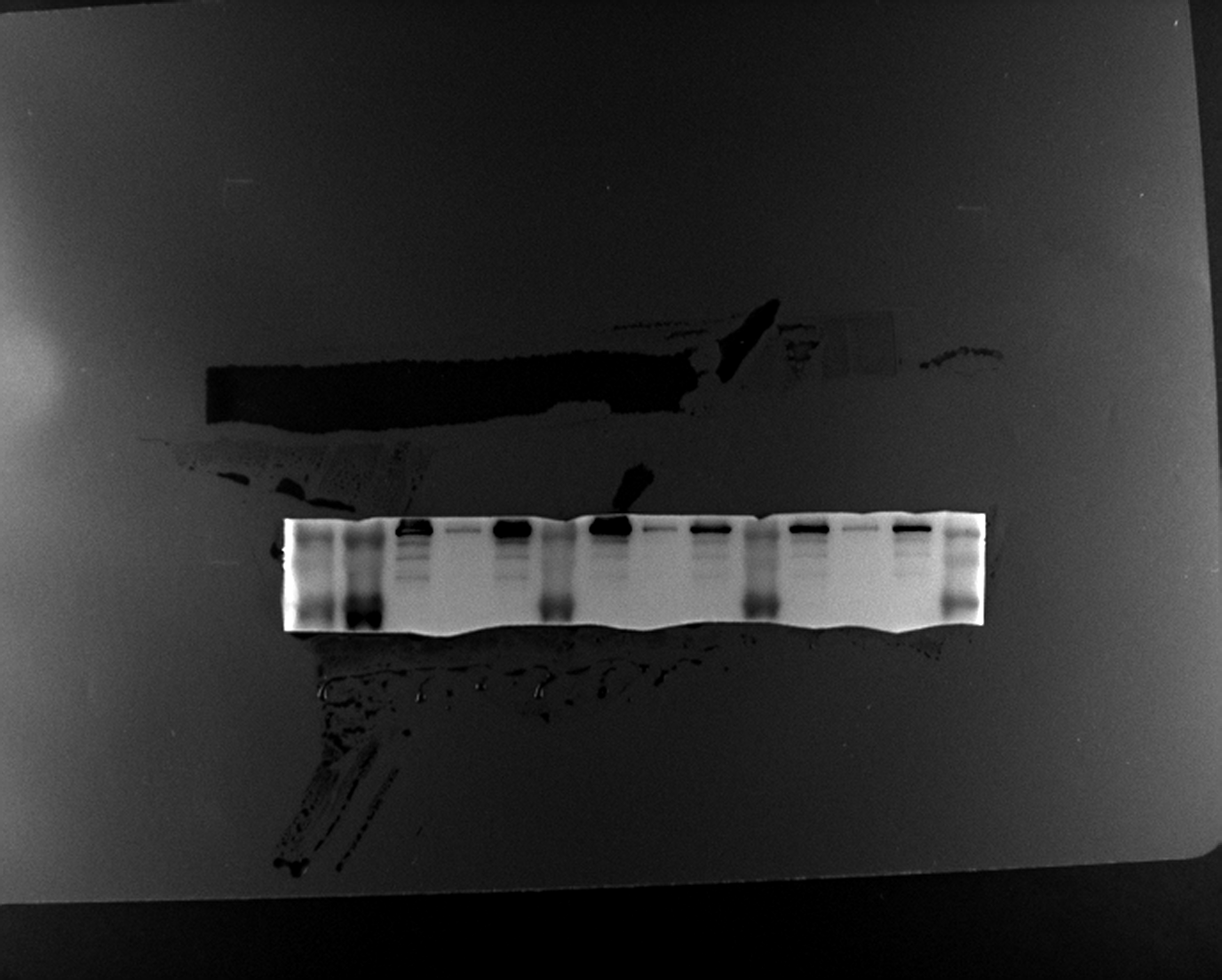

Supplement: Figure 7—source data 1. [file elife-76157-fig7-data1.zip › Figure 7-source data 1/Figure 7J-row 2.tif]

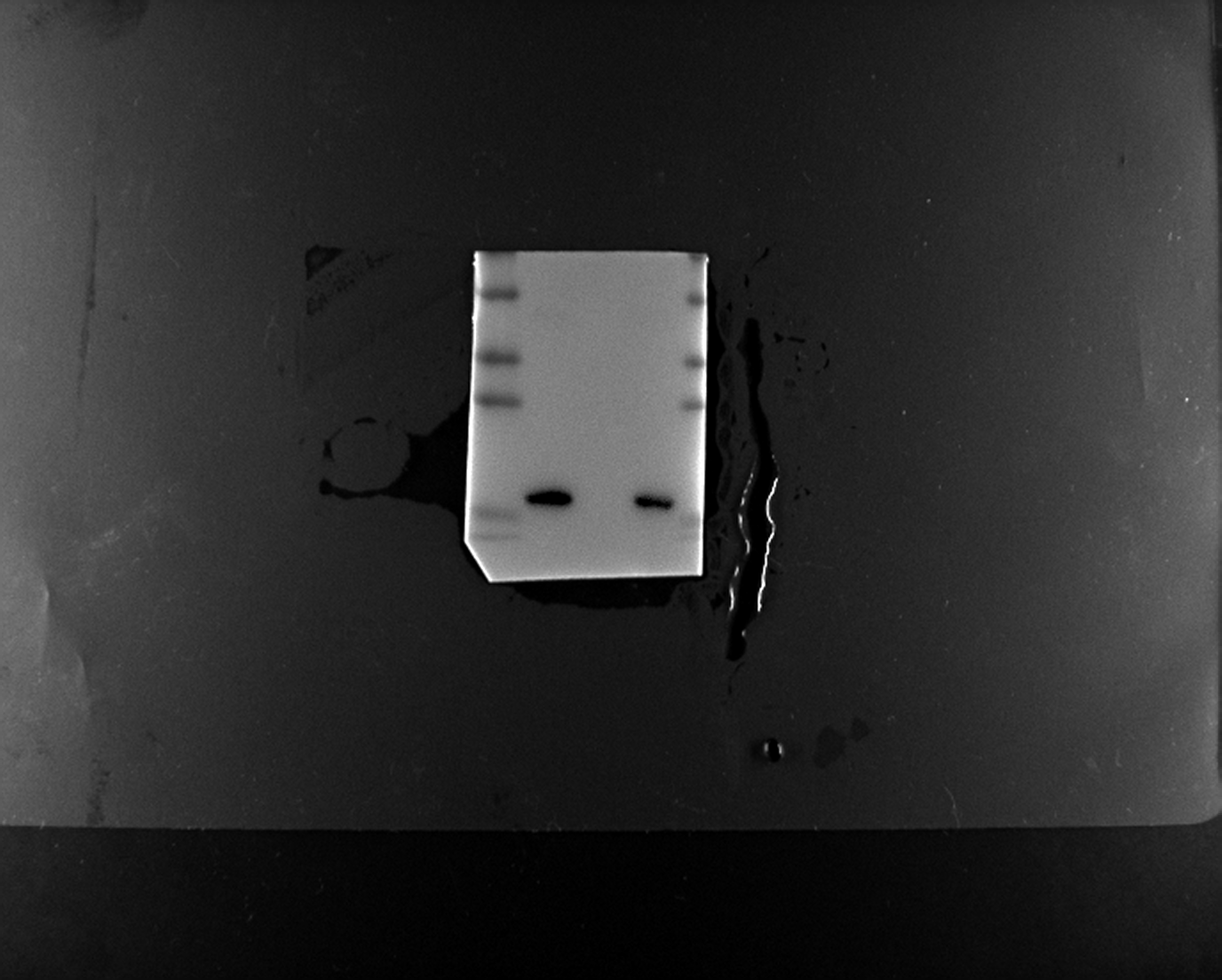

Supplement: Figure 7—source data 1. [file elife-76157-fig7-data1.zip › Figure 7-source data 1/Figure 7A-row 6.tif]

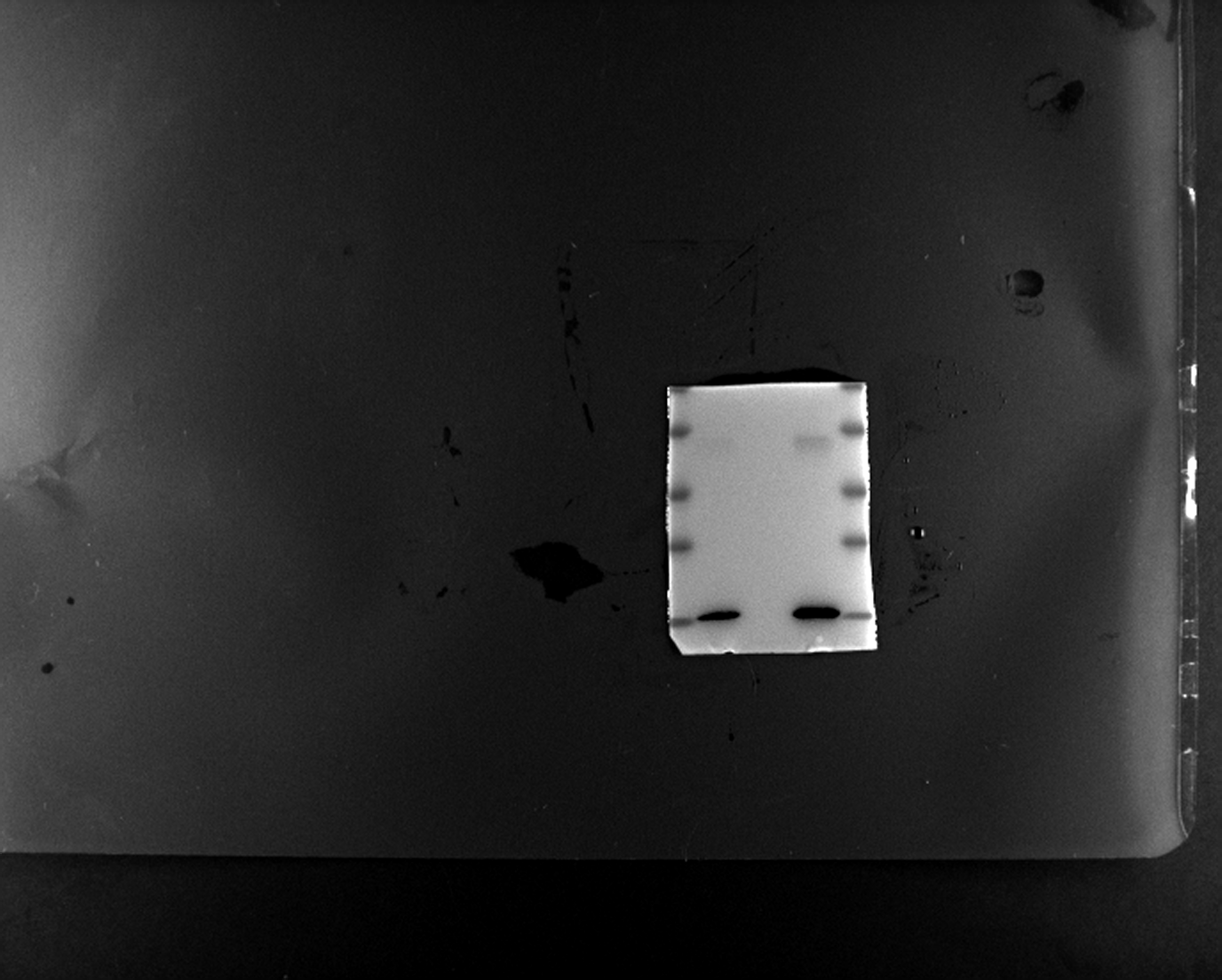

Supplement: Figure 7—source data 1. [file elife-76157-fig7-data1.zip › Figure 7-source data 1/Figure 7A-row 4.tif]

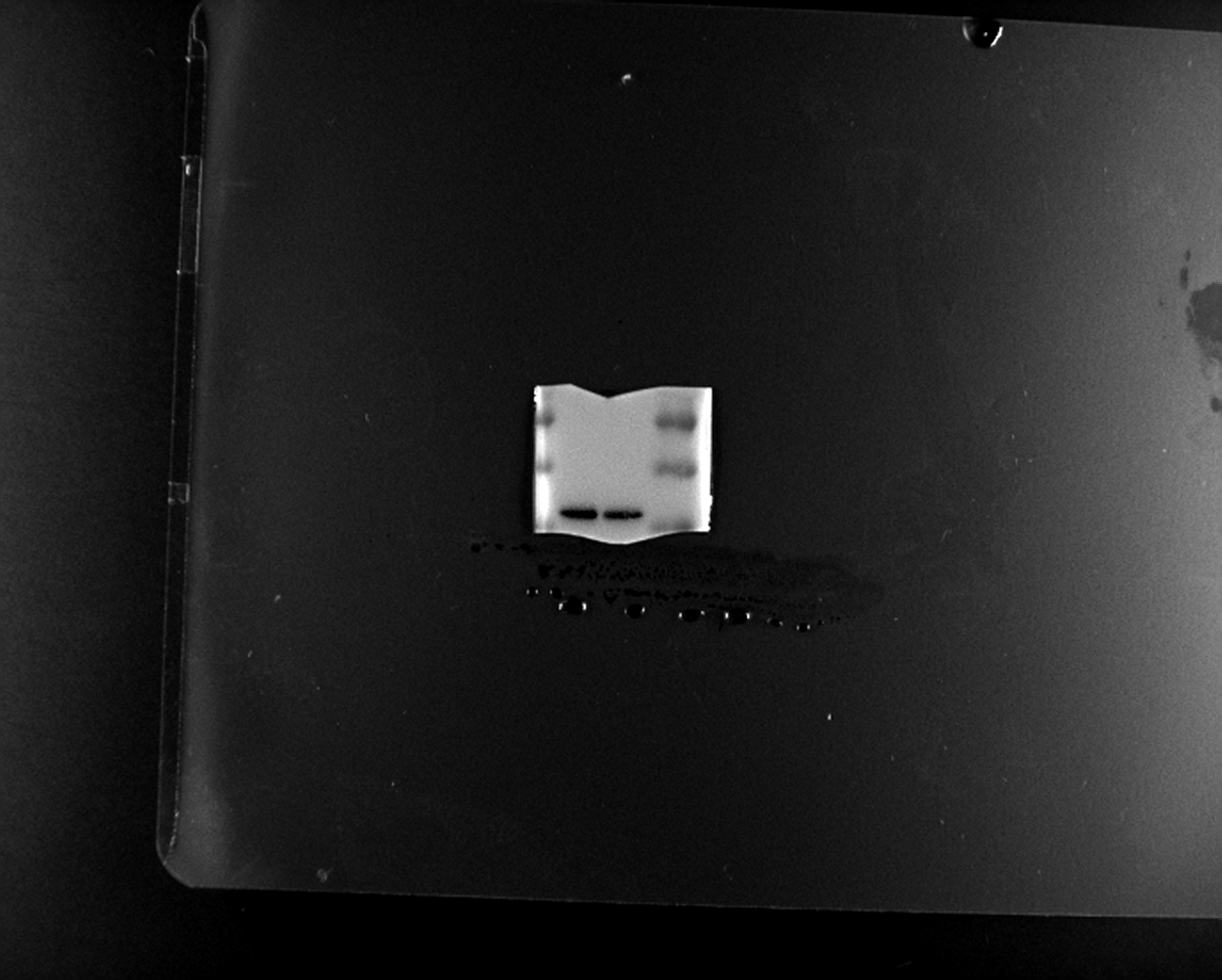

Supplement: Figure 7—source data 1. [file elife-76157-fig7-data1.zip › Figure 7-source data 1/Figure 7G-row 3.tif]

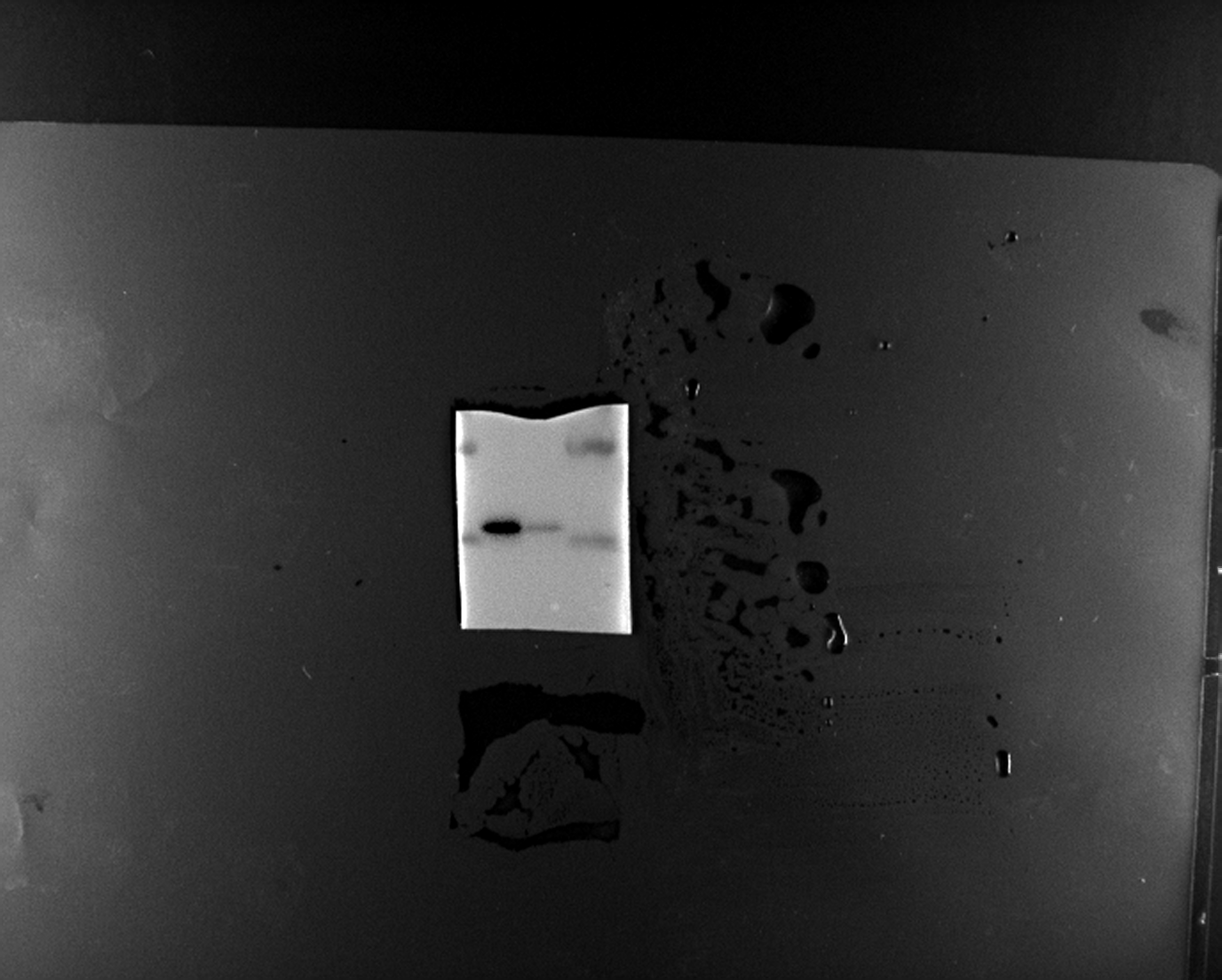

Supplement: Figure 7—source data 1. [file elife-76157-fig7-data1.zip › Figure 7-source data 1/Figure 7G-row 2.tif]

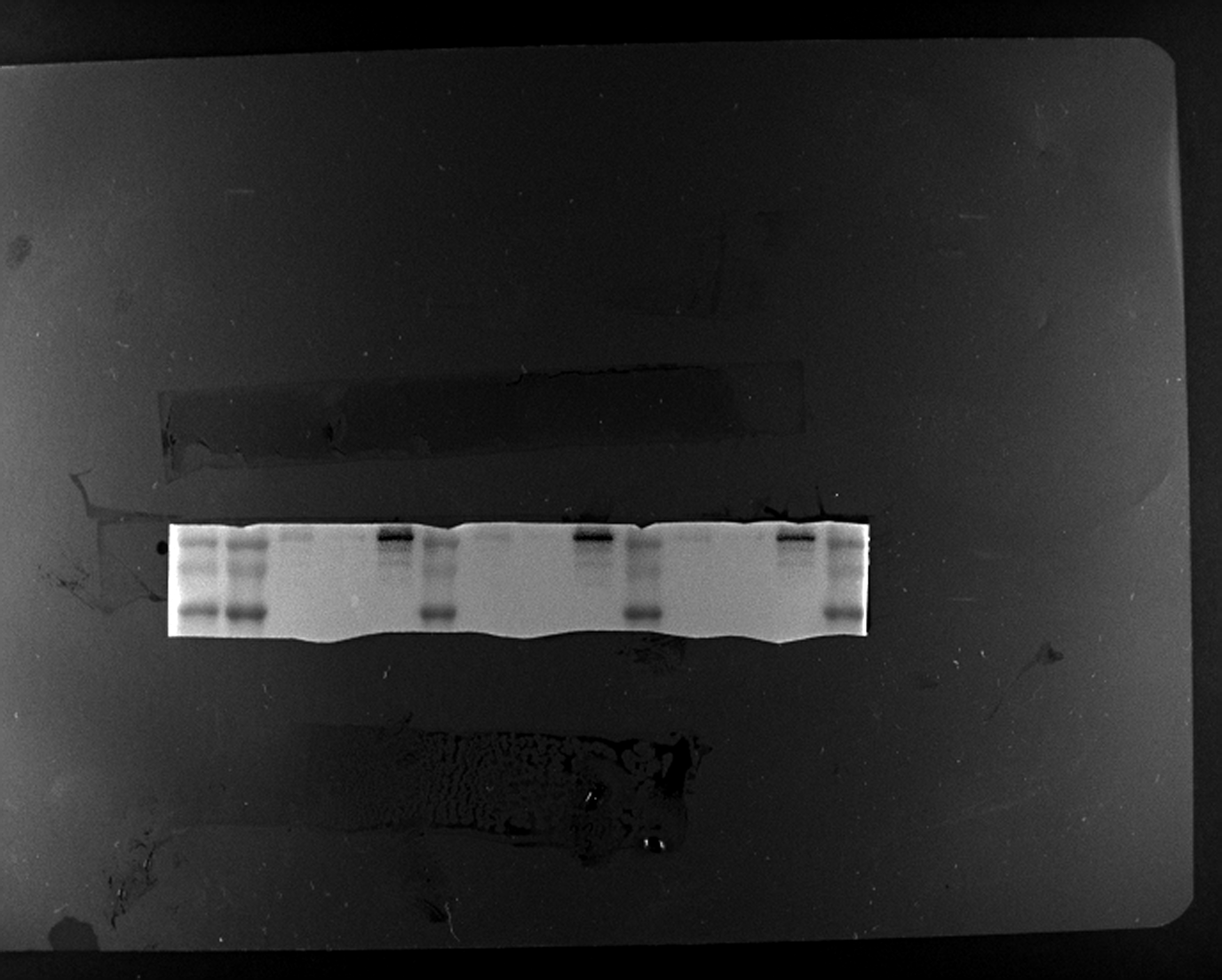

Supplement: Figure 7—source data 1. [file elife-76157-fig7-data1.zip › Figure 7-source data 1/Figure 7J-row 1.tif]

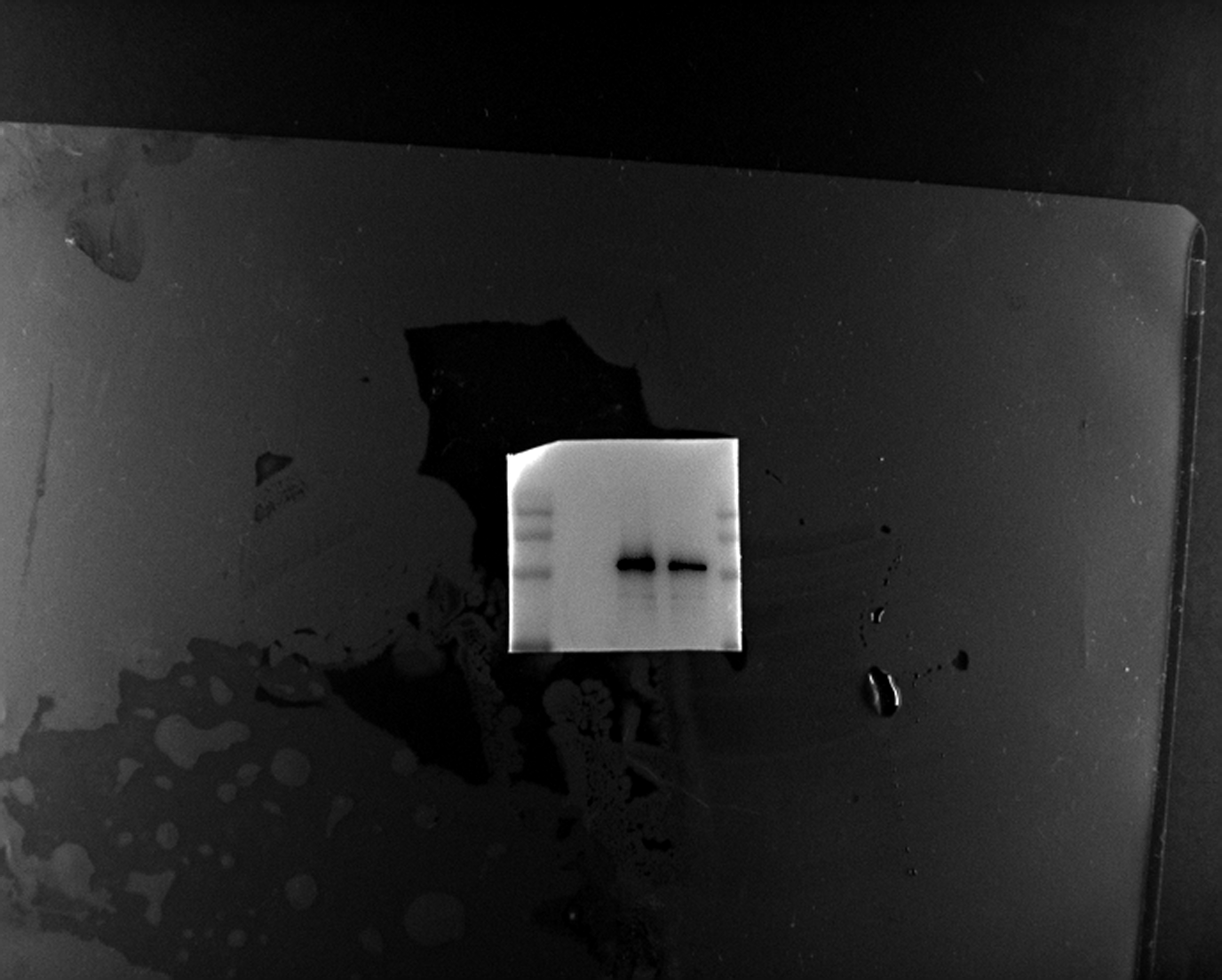

Supplement: Figure 7—source data 1. [file elife-76157-fig7-data1.zip › Figure 7-source data 1/Figure 7A-row 5.tif]

Figure 7-figure supplement 1-source data

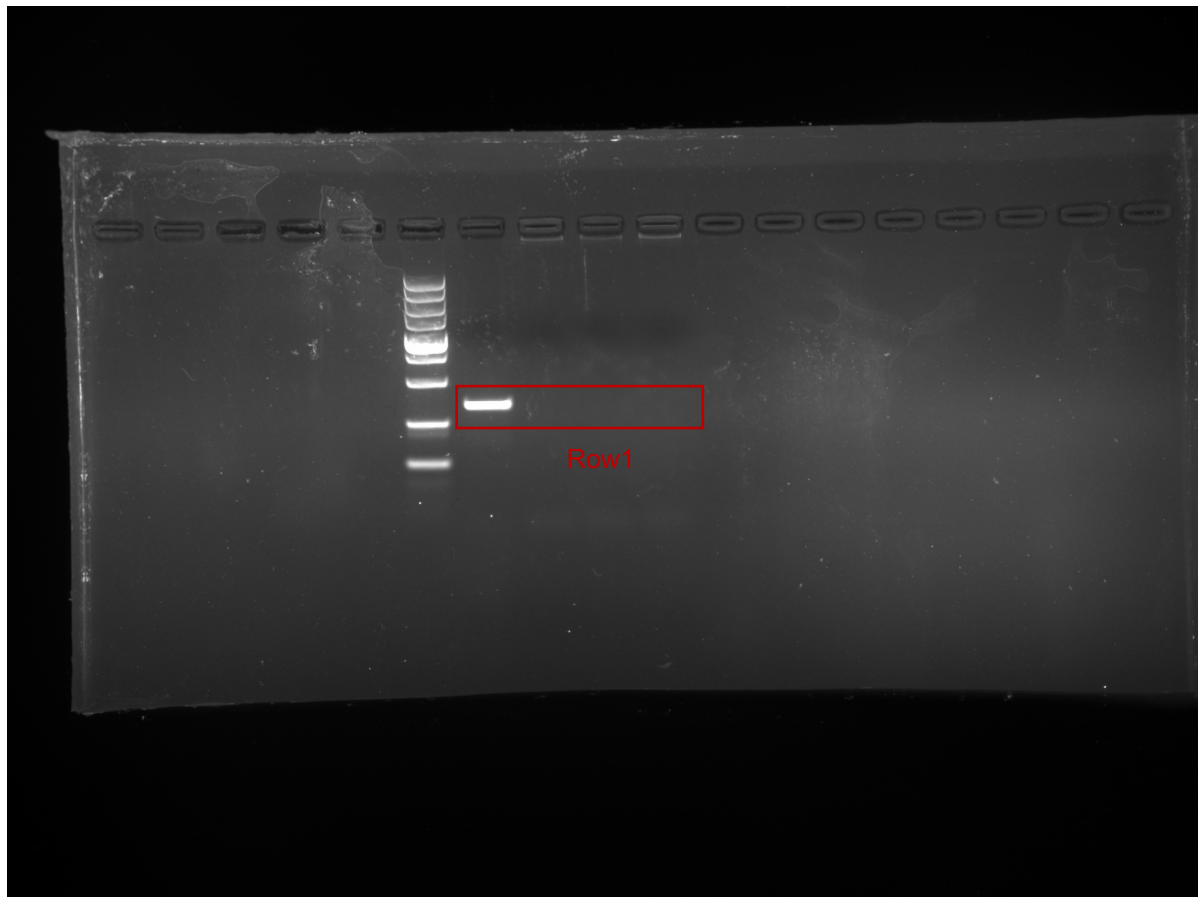

Supplement: Figure 7—figure supplement 1—source data 1. [file elife-76157-fig7-figsupp1-data1.zip › Figure 7-figure supplement 1-source data 1/Figure 7-figure supplement 1-labeled.pdf]

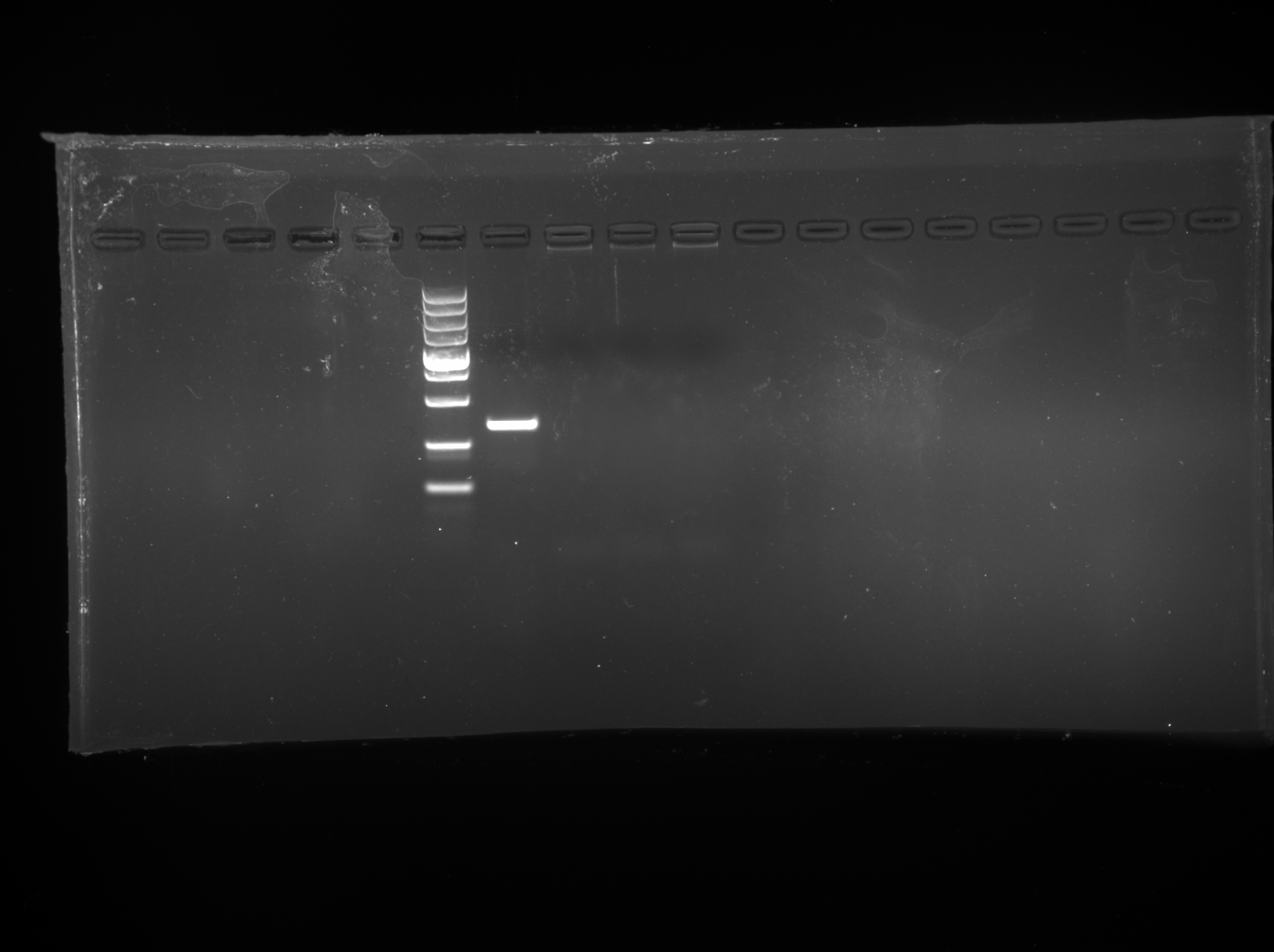

Supplement: Figure 7—figure supplement 1—source data 1. [file elife-76157-fig7-figsupp1-data1.zip › Figure 7-figure supplement 1-source data 1/Figure 7-figure supplement 1 row 1.tif]

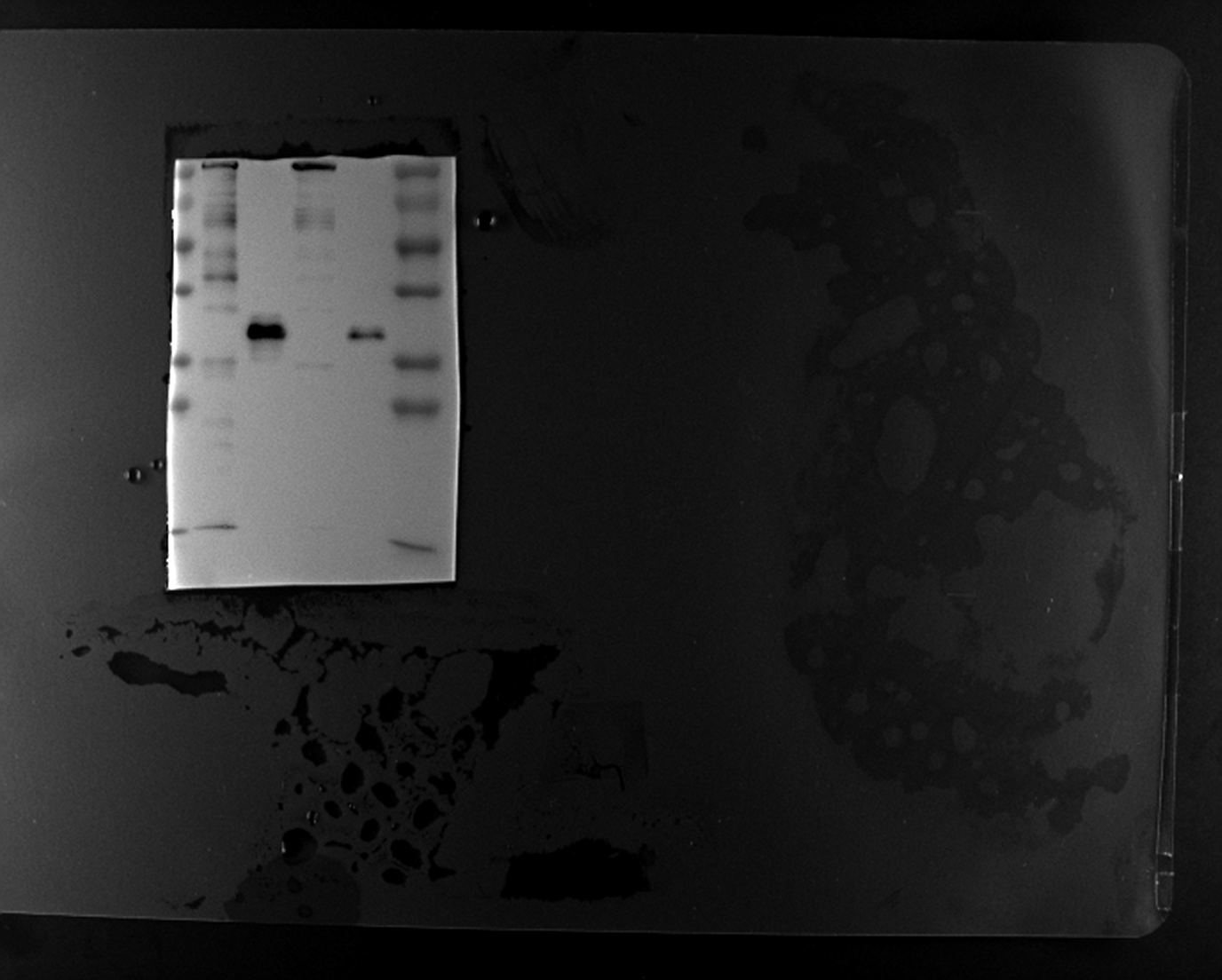

Supplement: Figure 7—figure supplement 2—source data 1. [file elife-76157-fig7-figsupp2-data1.zip › Figure 7-figure supplement 2-source data 1/Figure 7-figure supplement 2B-row 1,2,4&5.tif]

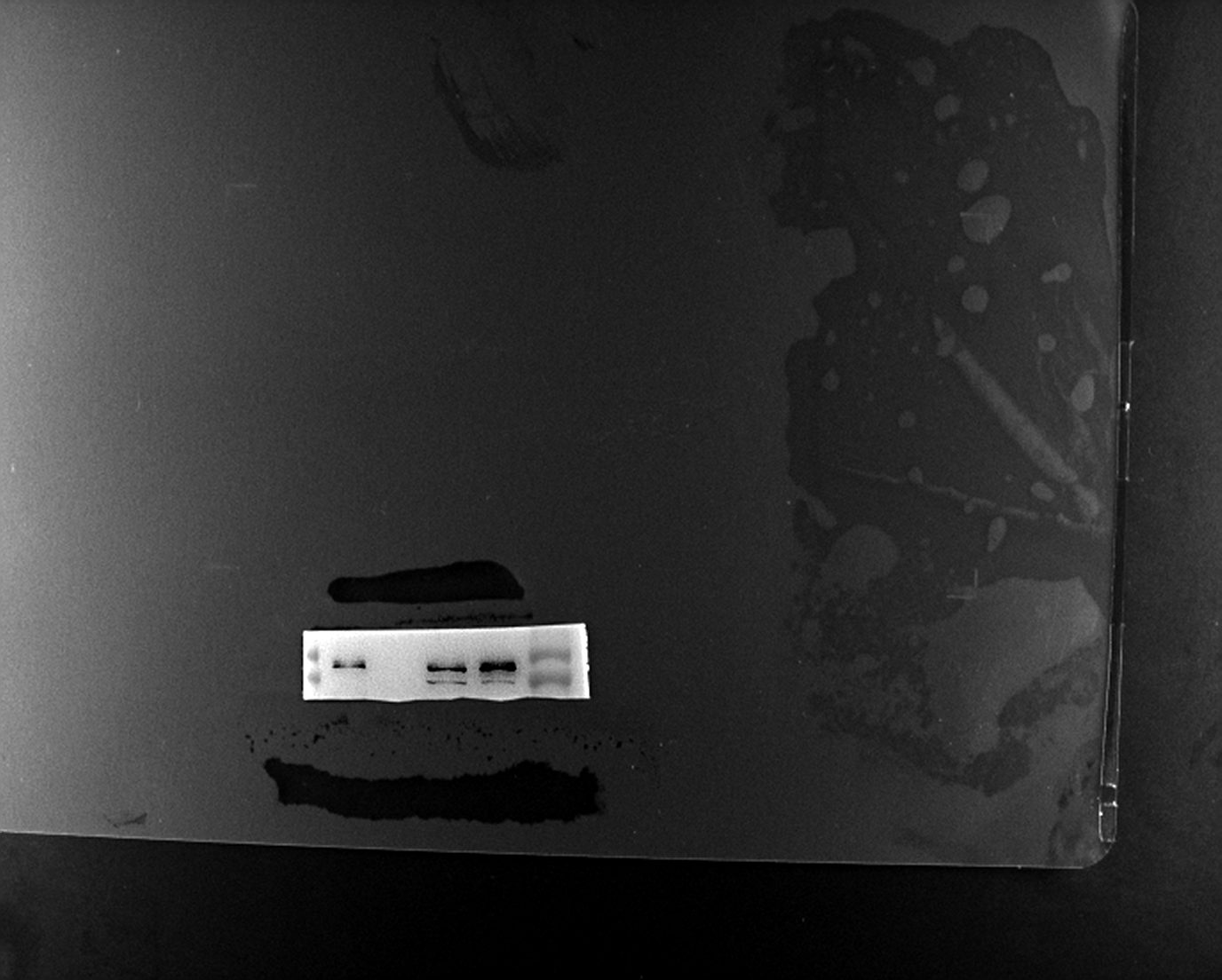

Supplement: Figure 7—figure supplement 2—source data 1. [file elife-76157-fig7-figsupp2-data1.zip › Figure 7-figure supplement 2-source data 1/Figure 7-figure supplement 2B-row 3&6.tif]

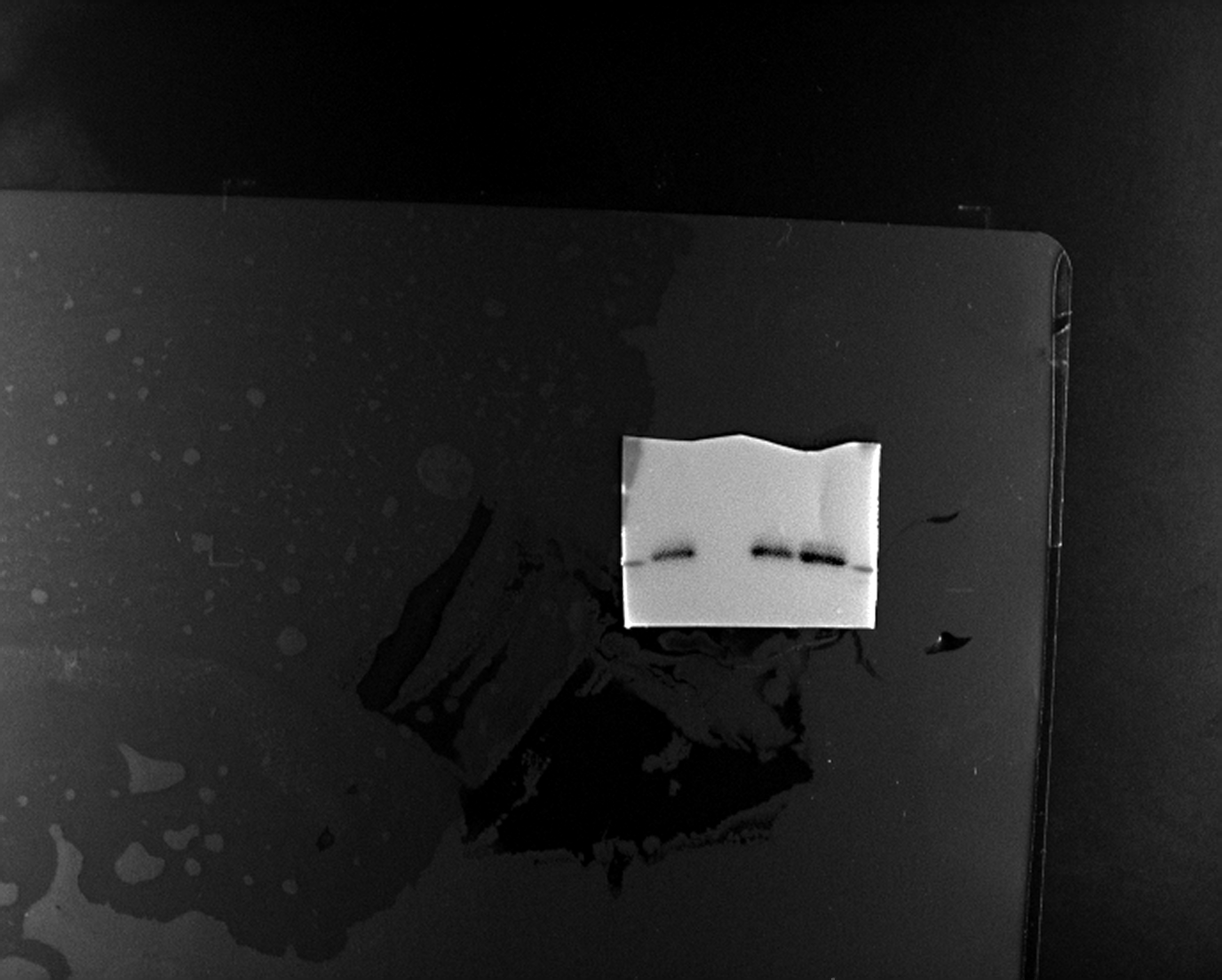

Supplement: Figure 7—figure supplement 2—source data 1. [file elife-76157-fig7-figsupp2-data1.zip › Figure 7-figure supplement 2-source data 1/Figure 7-figure supplement 2A-row 3&6.tif]

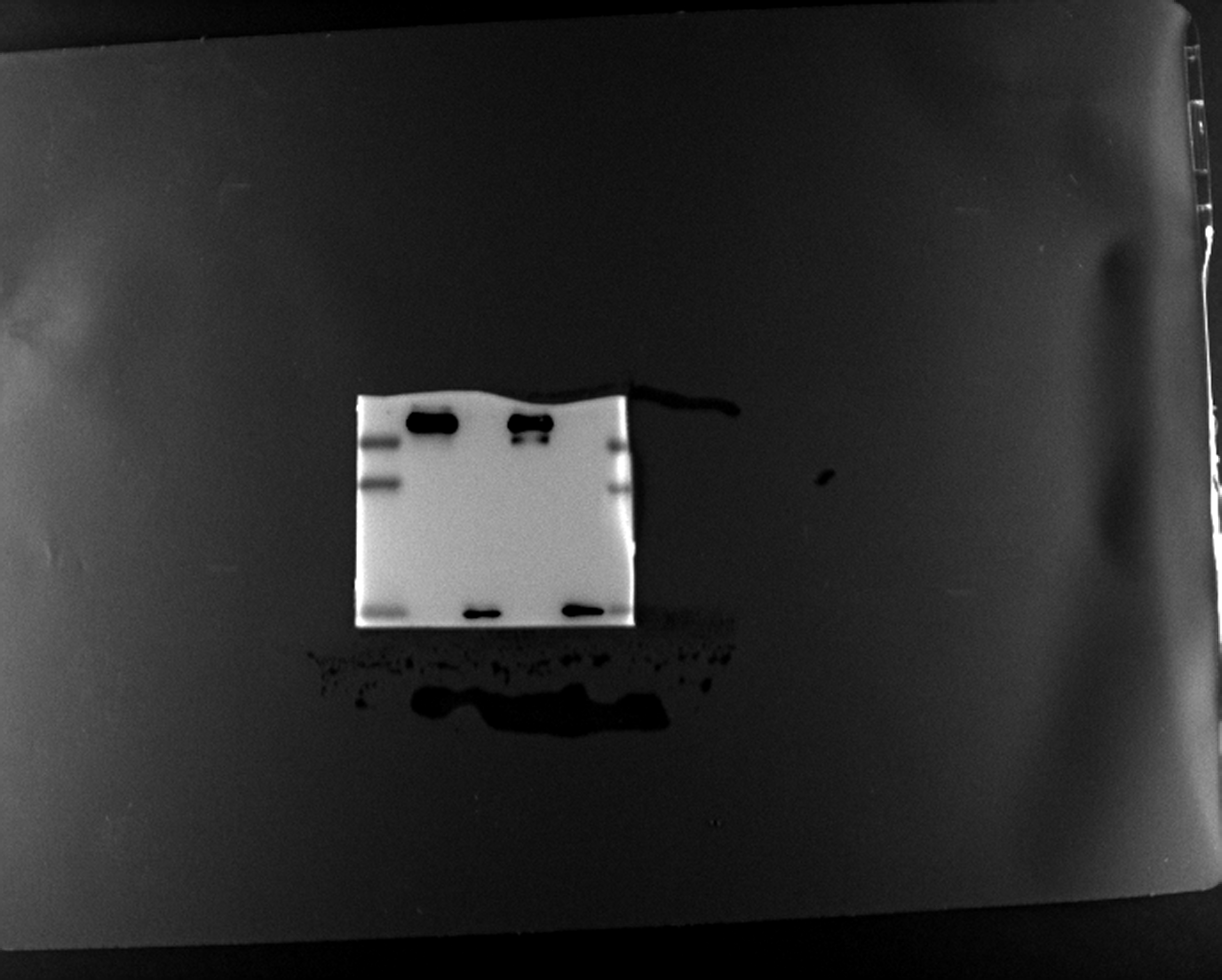

Supplement: Figure 7—figure supplement 2—source data 1. [file elife-76157-fig7-figsupp2-data1.zip › Figure 7-figure supplement 2-source data 1/Figure 7-figure supplement 2C-row 1,2,4&5.tif]

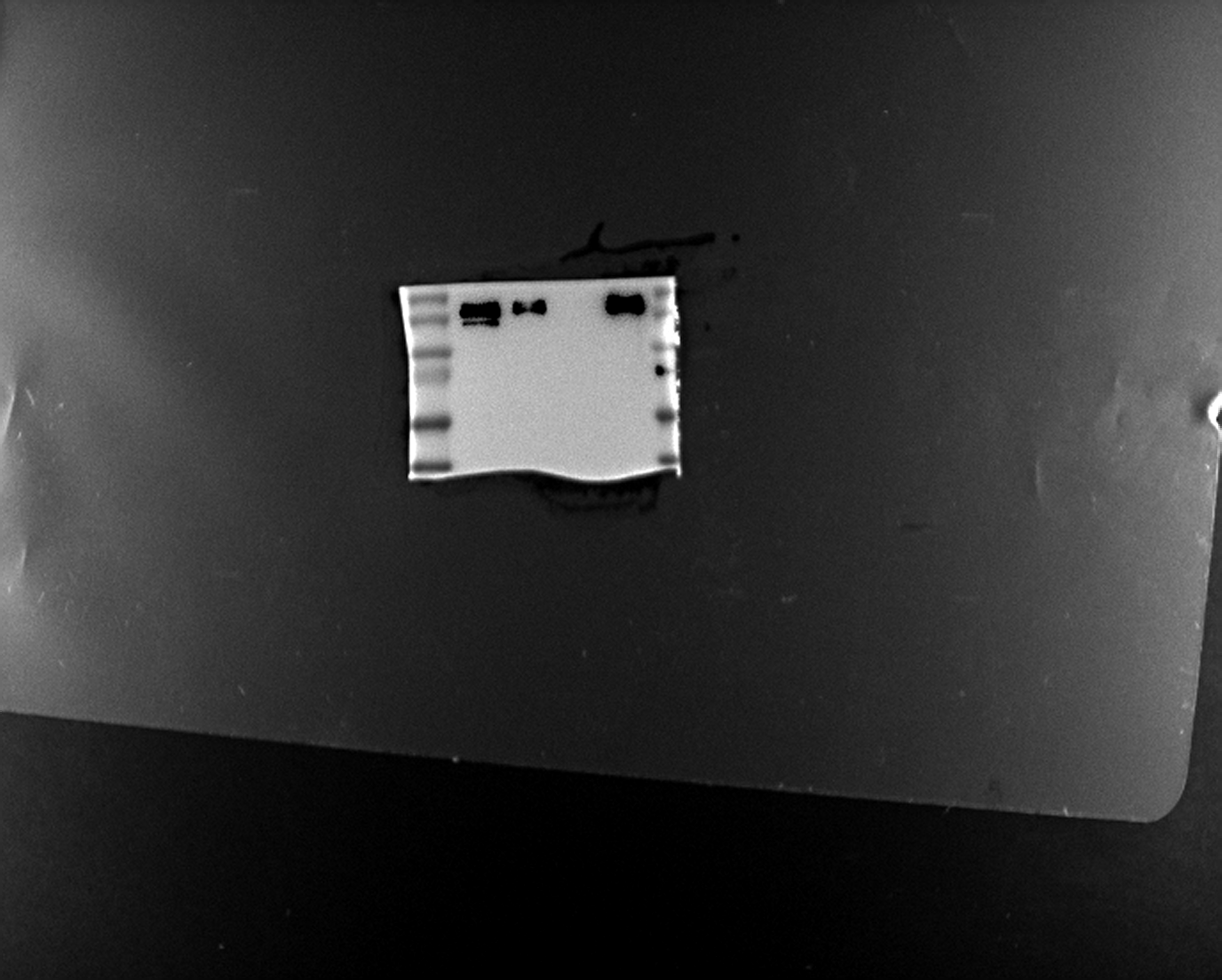

Supplement: Figure 7—figure supplement 2—source data 1. [file elife-76157-fig7-figsupp2-data1.zip › Figure 7-figure supplement 2-source data 1/Figure 7-figure supplement 2C-row 3&6.tif]

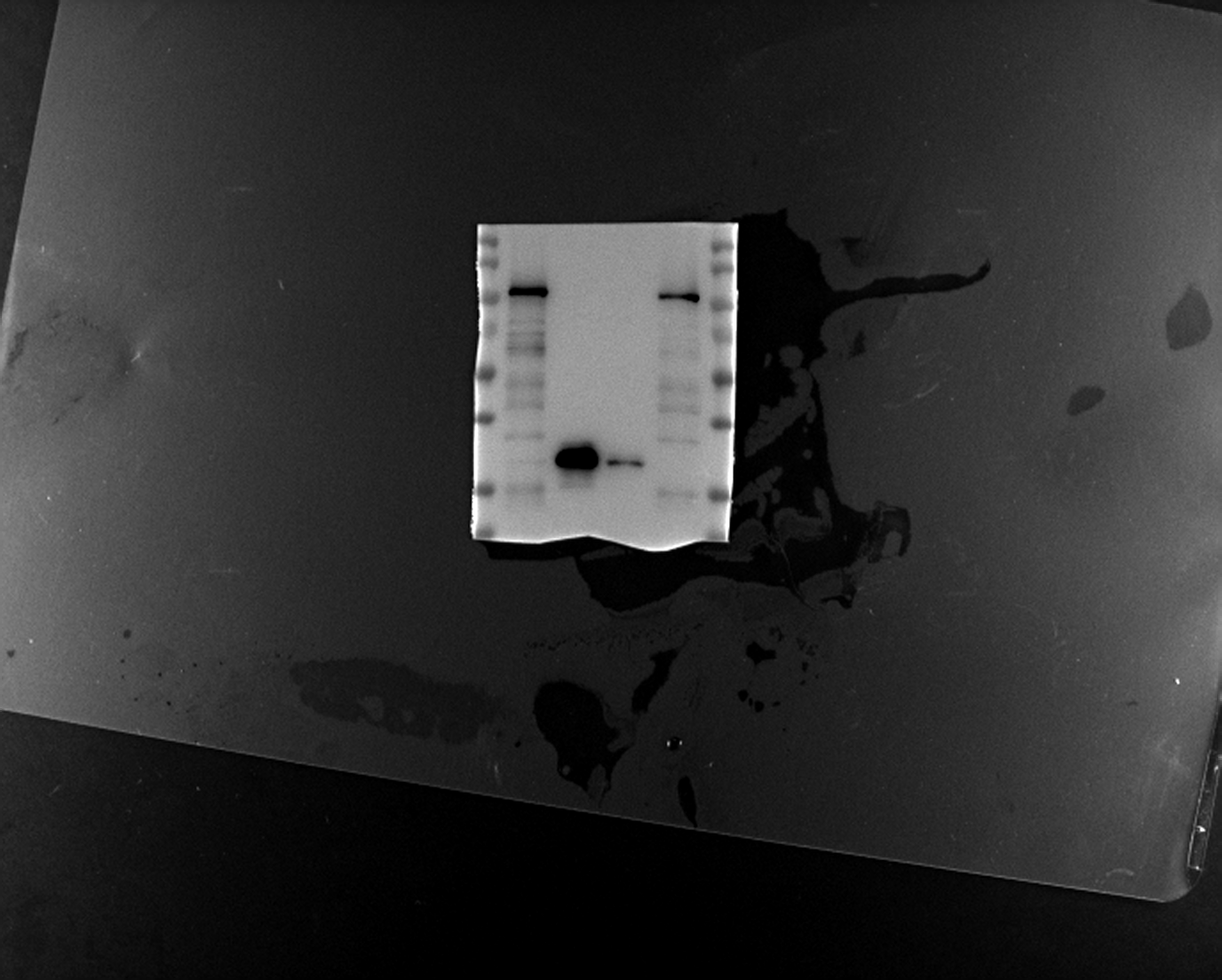

Supplement: Figure 7—figure supplement 2—source data 1. [file elife-76157-fig7-figsupp2-data1.zip › Figure 7-figure supplement 2-source data 1/Figure 7-figure supplement 2A-row 1,2,4&5.tif]

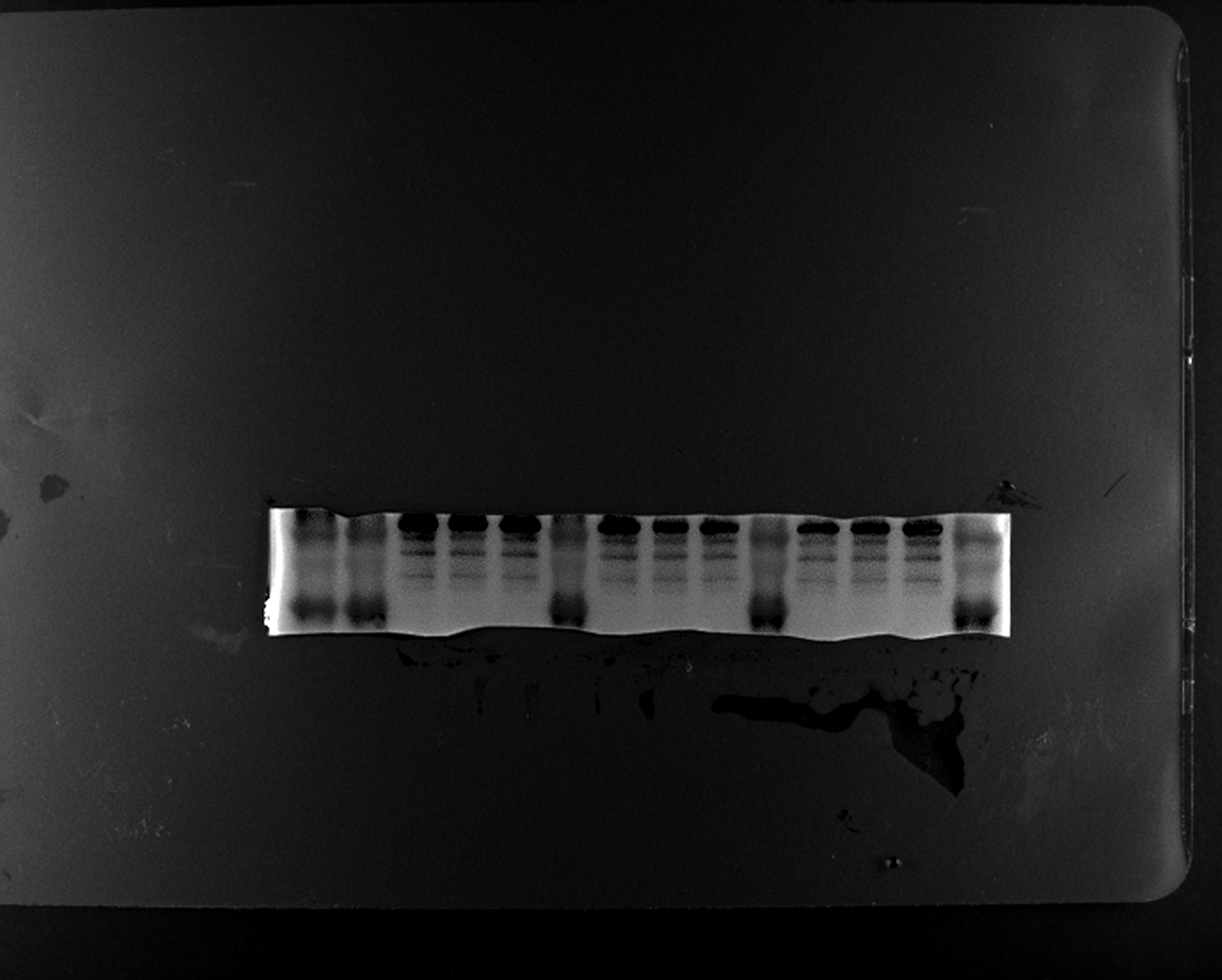

Supplement: Figure 7—figure supplement 3—source data 1. [file elife-76157-fig7-figsupp3-data1.zip › Figure 7-figure supplement 3-source data 1/Figure 7-figure supplement 3H-row 3.tif]

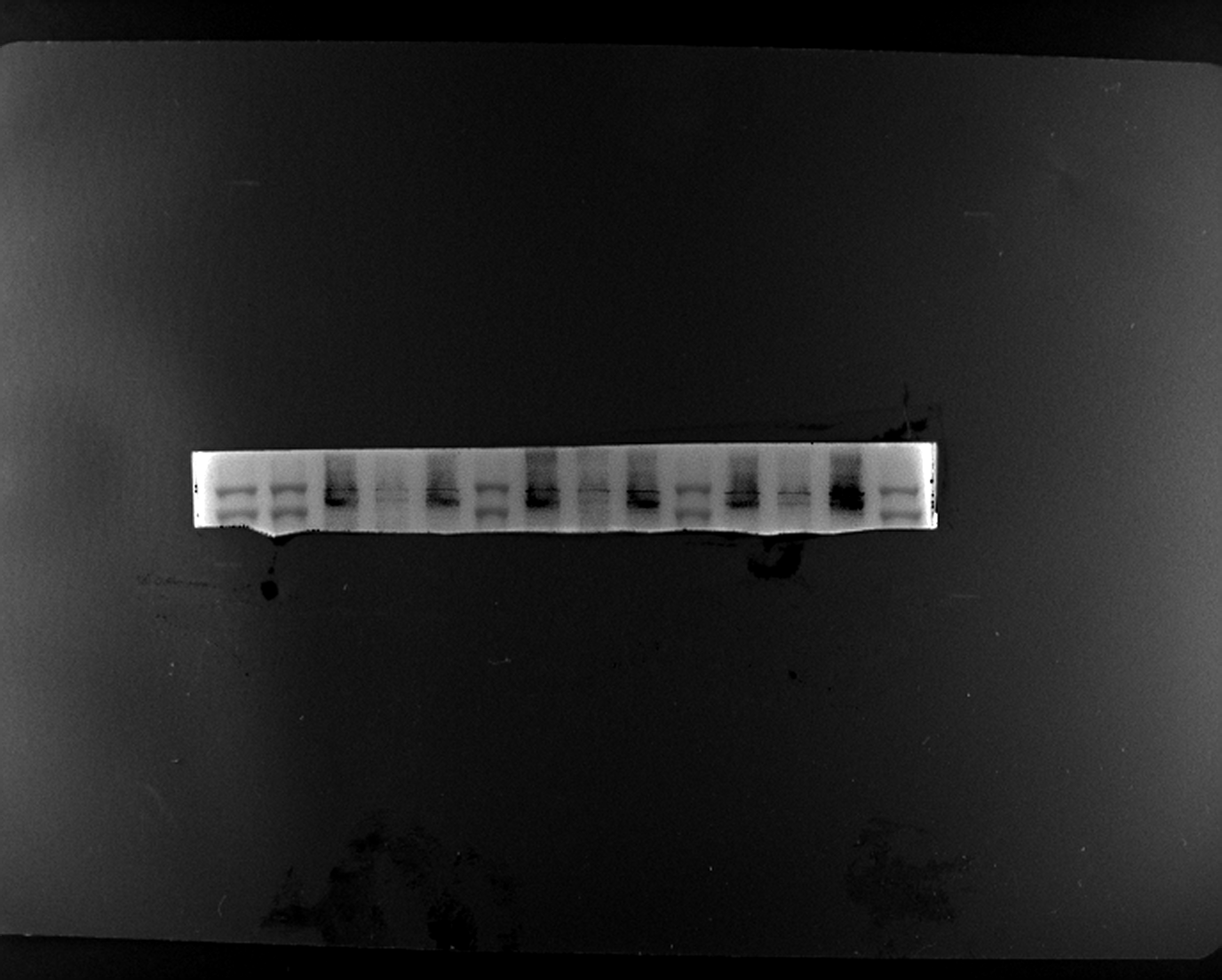

Supplement: Figure 7—figure supplement 3—source data 1. [file elife-76157-fig7-figsupp3-data1.zip › Figure 7-figure supplement 3-source data 1/Figure 7-figure supplement 3H-row 2.tif]

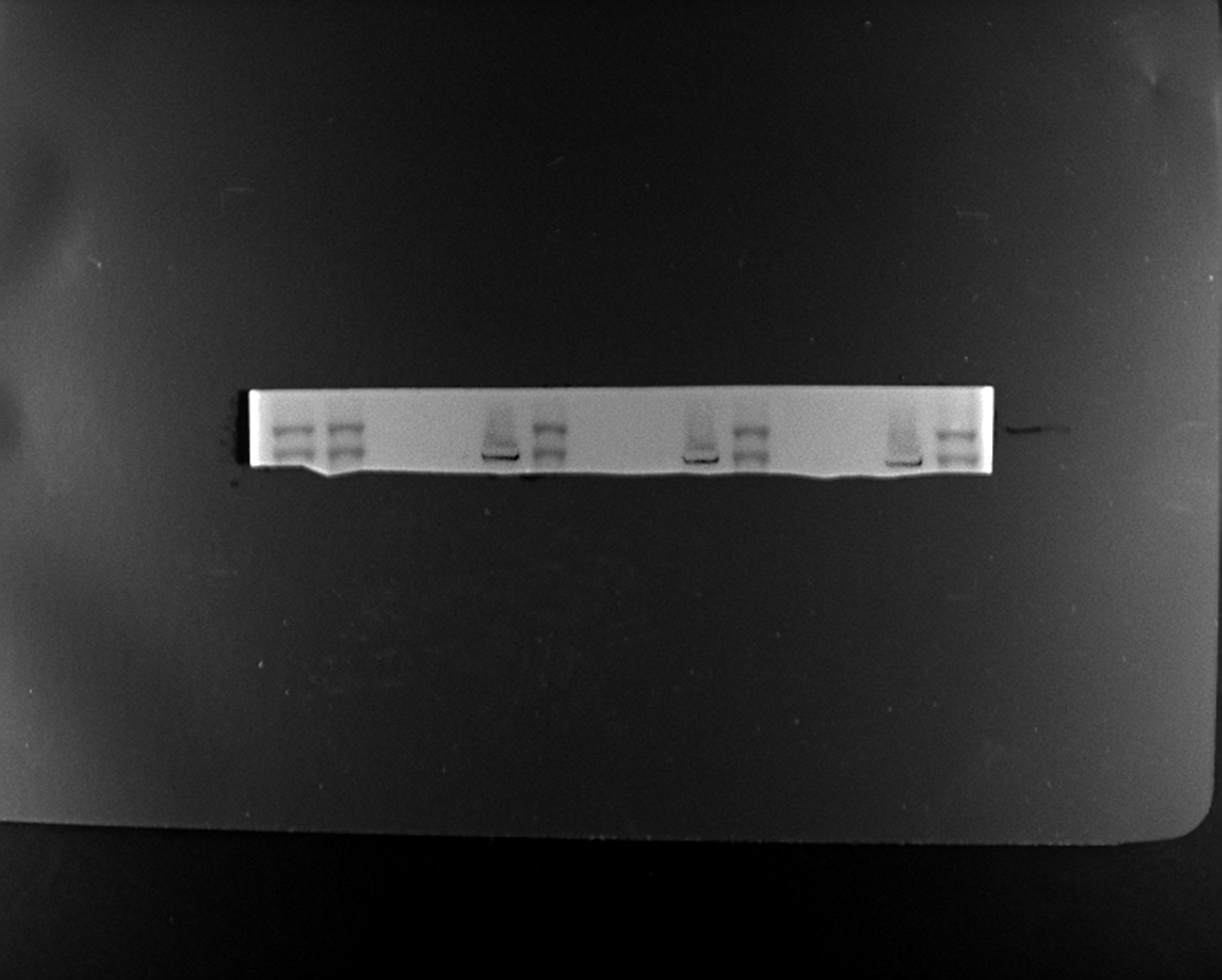

Supplement: Figure 7—figure supplement 3—source data 1. [file elife-76157-fig7-figsupp3-data1.zip › Figure 7-figure supplement 3-source data 1/Figure 7-figure supplement 3H-row 1.tif]

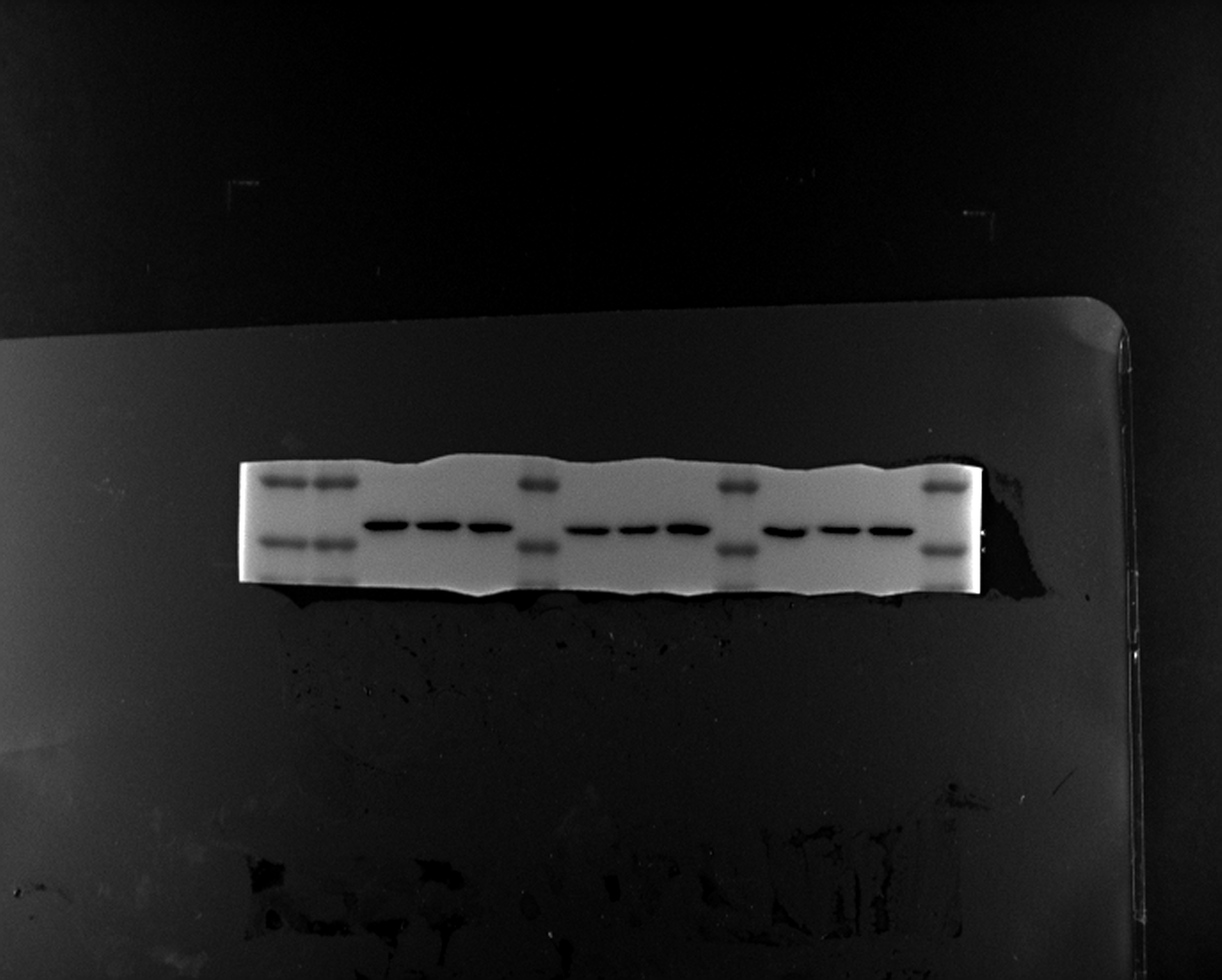

Supplement: Figure 7—figure supplement 3—source data 1. [file elife-76157-fig7-figsupp3-data1.zip › Figure 7-figure supplement 3-source data 1/Figure 7-figure supplement 3H-row 5.tif]

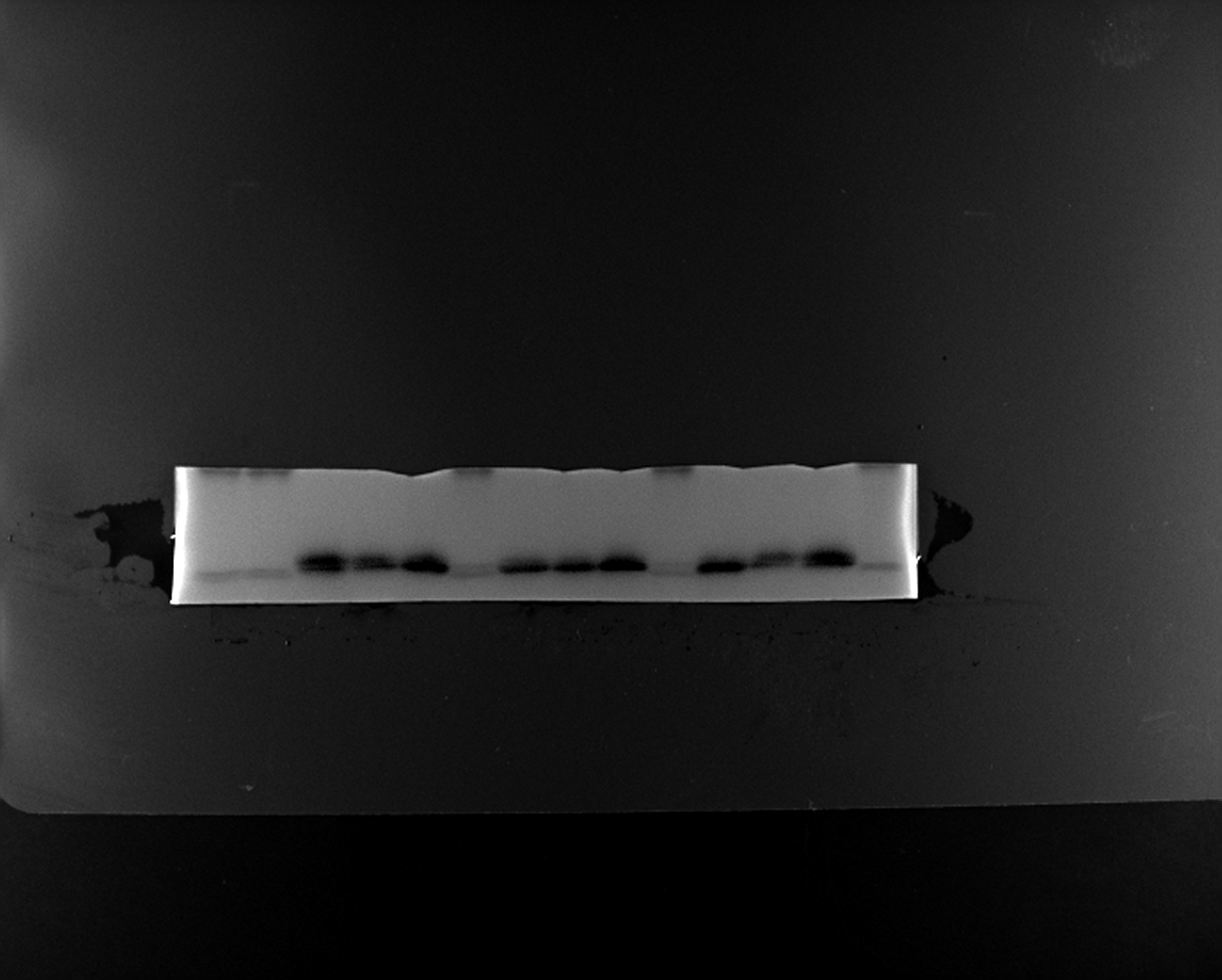

Supplement: Figure 7—figure supplement 3—source data 1. [file elife-76157-fig7-figsupp3-data1.zip › Figure 7-figure supplement 3-source data 1/Figure 7-figure supplement 3H-row 4.tif]

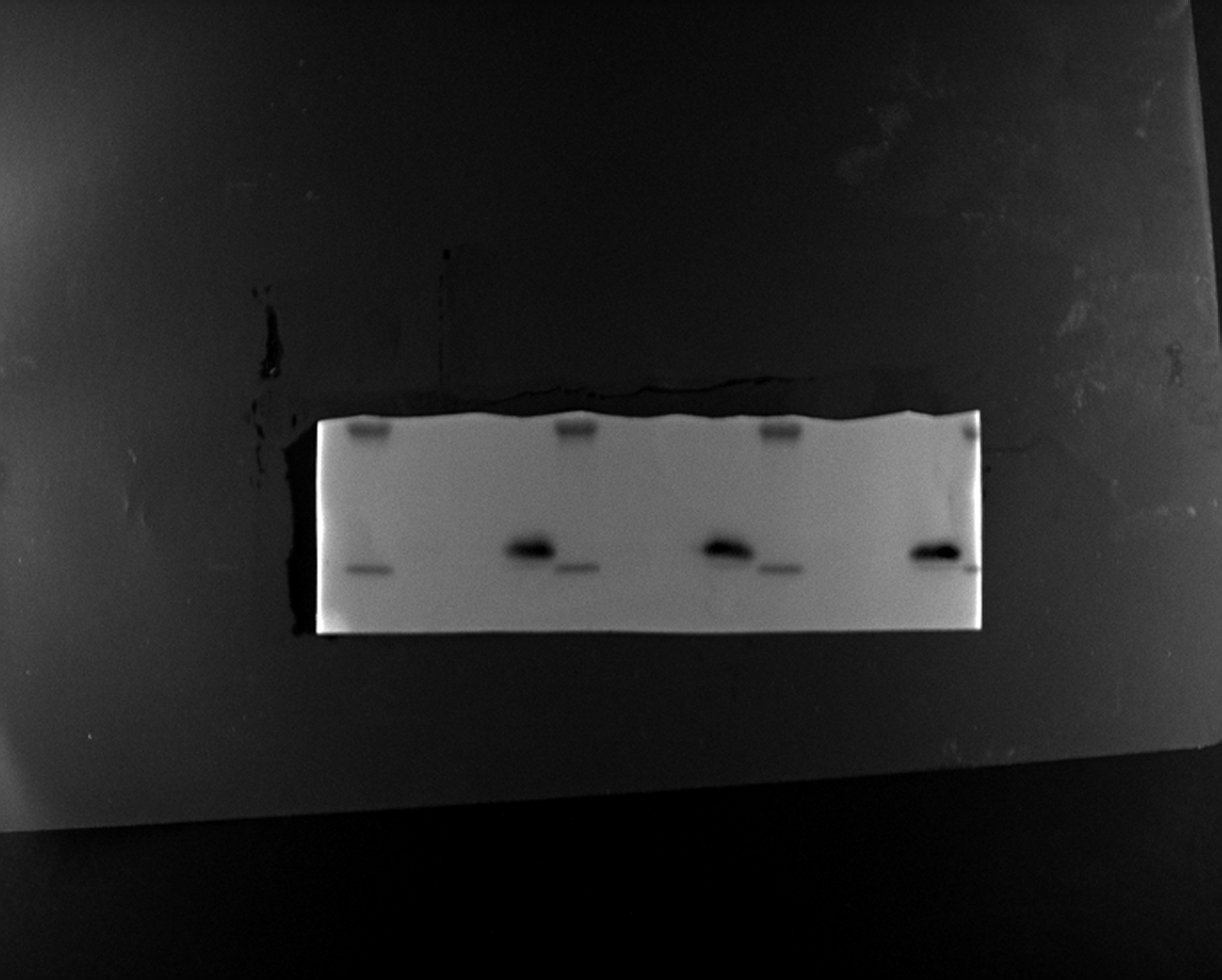

Supplement: Figure 7—figure supplement 3—source data 1. [file elife-76157-fig7-figsupp3-data1.zip › Figure 7-figure supplement 3-source data 1/Figure 7-figure supplement 3D-row 1.tif]

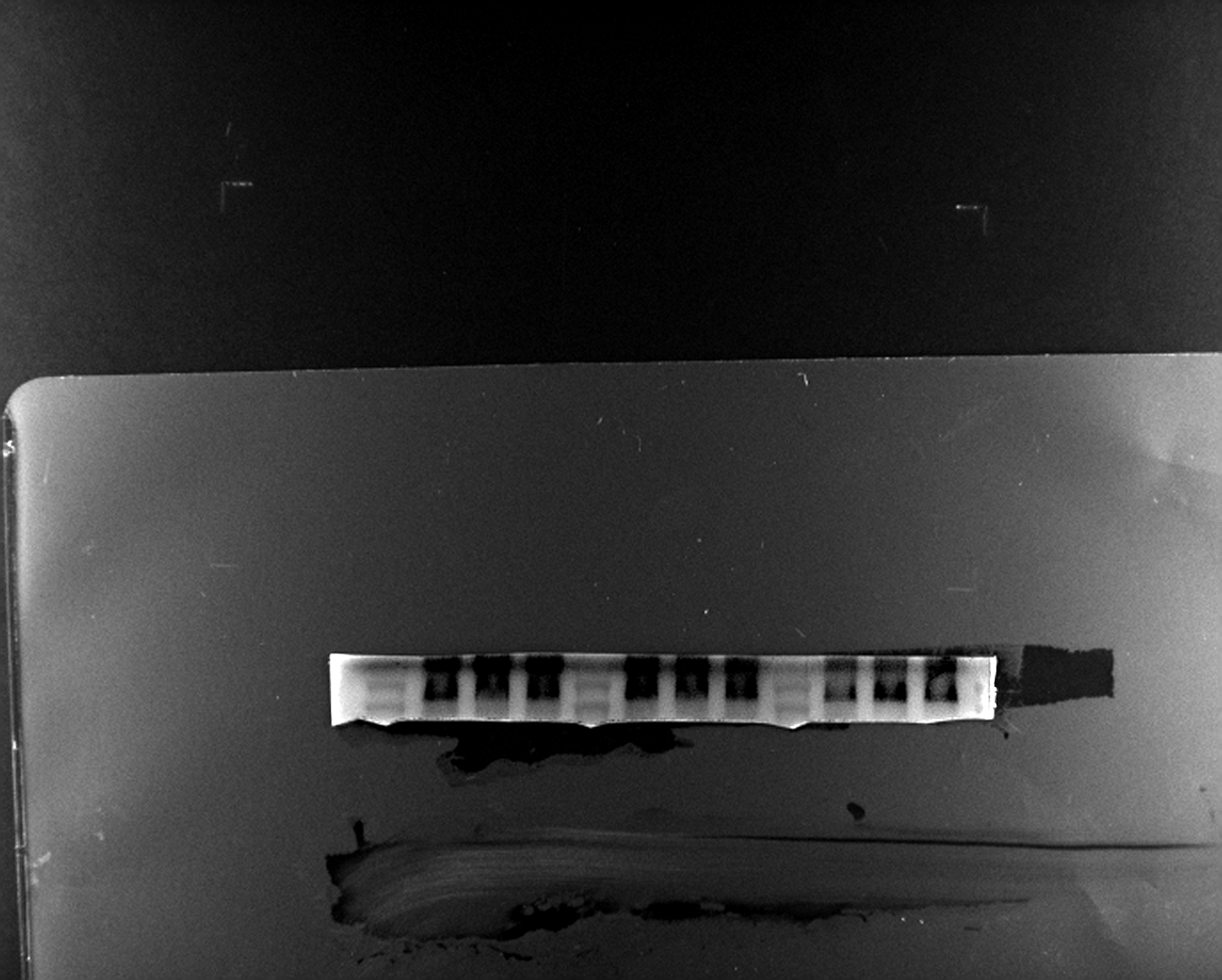

Supplement: Figure 7—figure supplement 3—source data 1. [file elife-76157-fig7-figsupp3-data1.zip › Figure 7-figure supplement 3-source data 1/Figure 7-figure supplement 3D-row 3.tif]

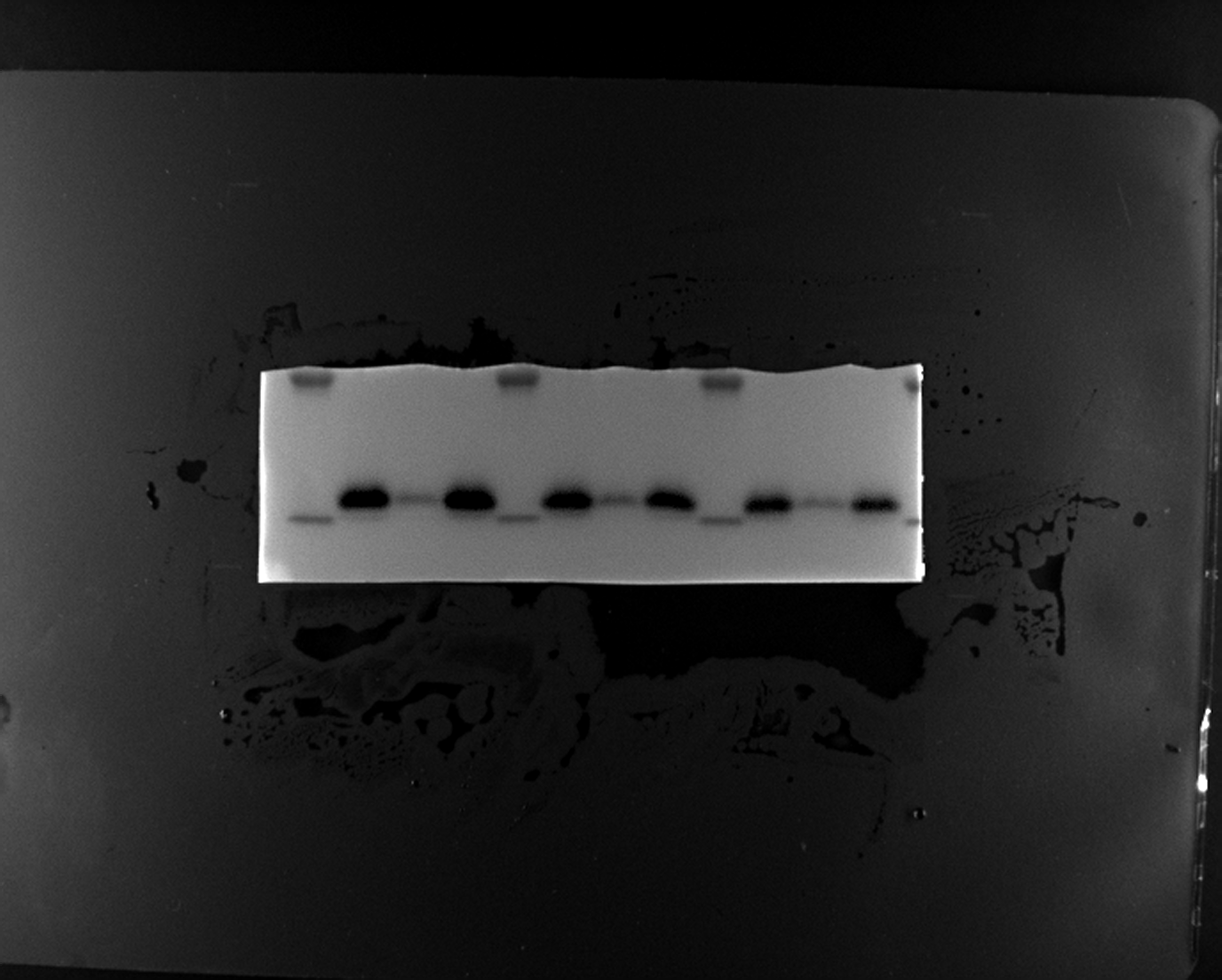

Supplement: Figure 7—figure supplement 3—source data 1. [file elife-76157-fig7-figsupp3-data1.zip › Figure 7-figure supplement 3-source data 1/Figure 7-figure supplement 3D-row 2.tif]

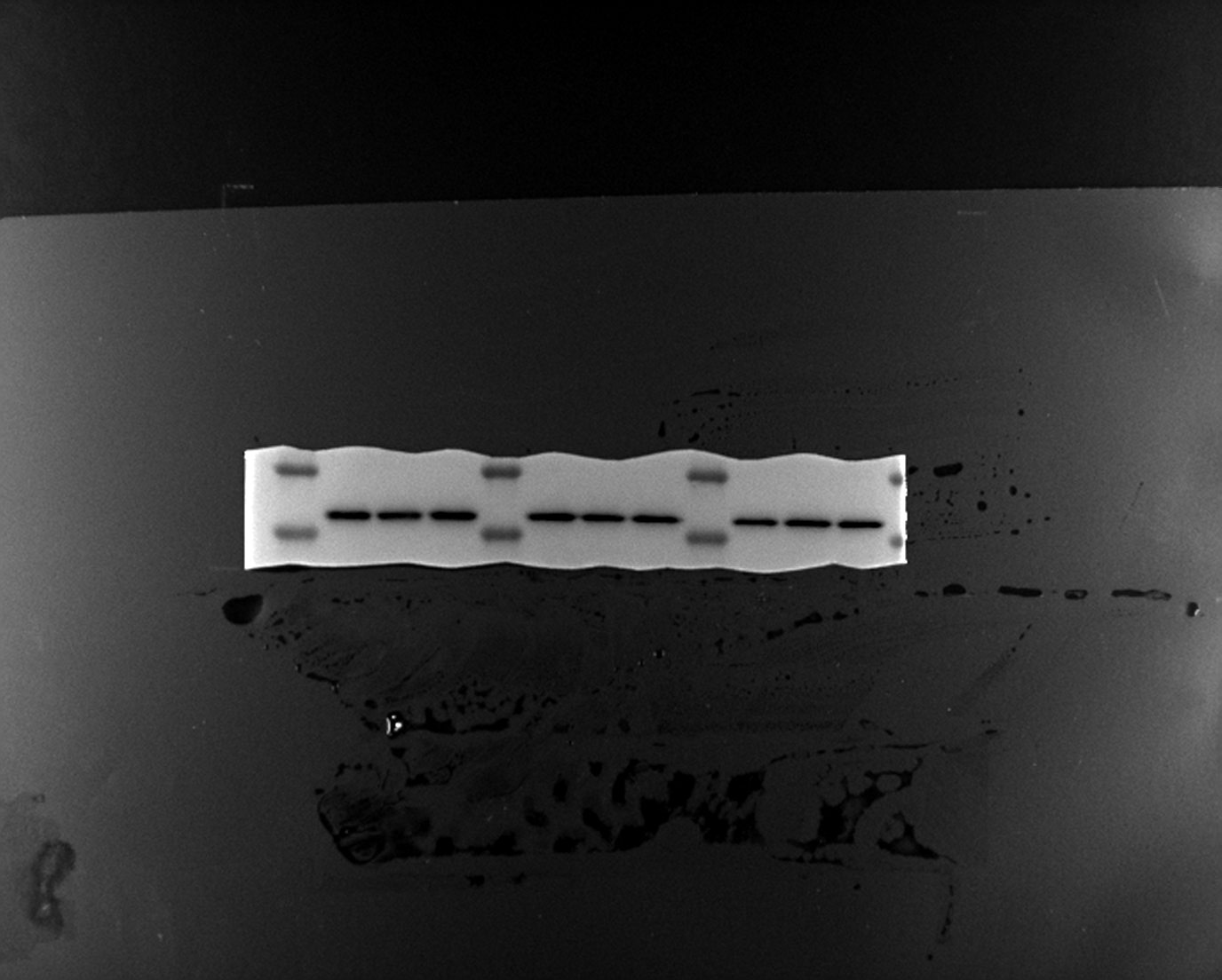

Supplement: Figure 7—figure supplement 3—source data 1. [file elife-76157-fig7-figsupp3-data1.zip › Figure 7-figure supplement 3-source data 1/Figure 7-figure supplement 3D-row 5.tif]

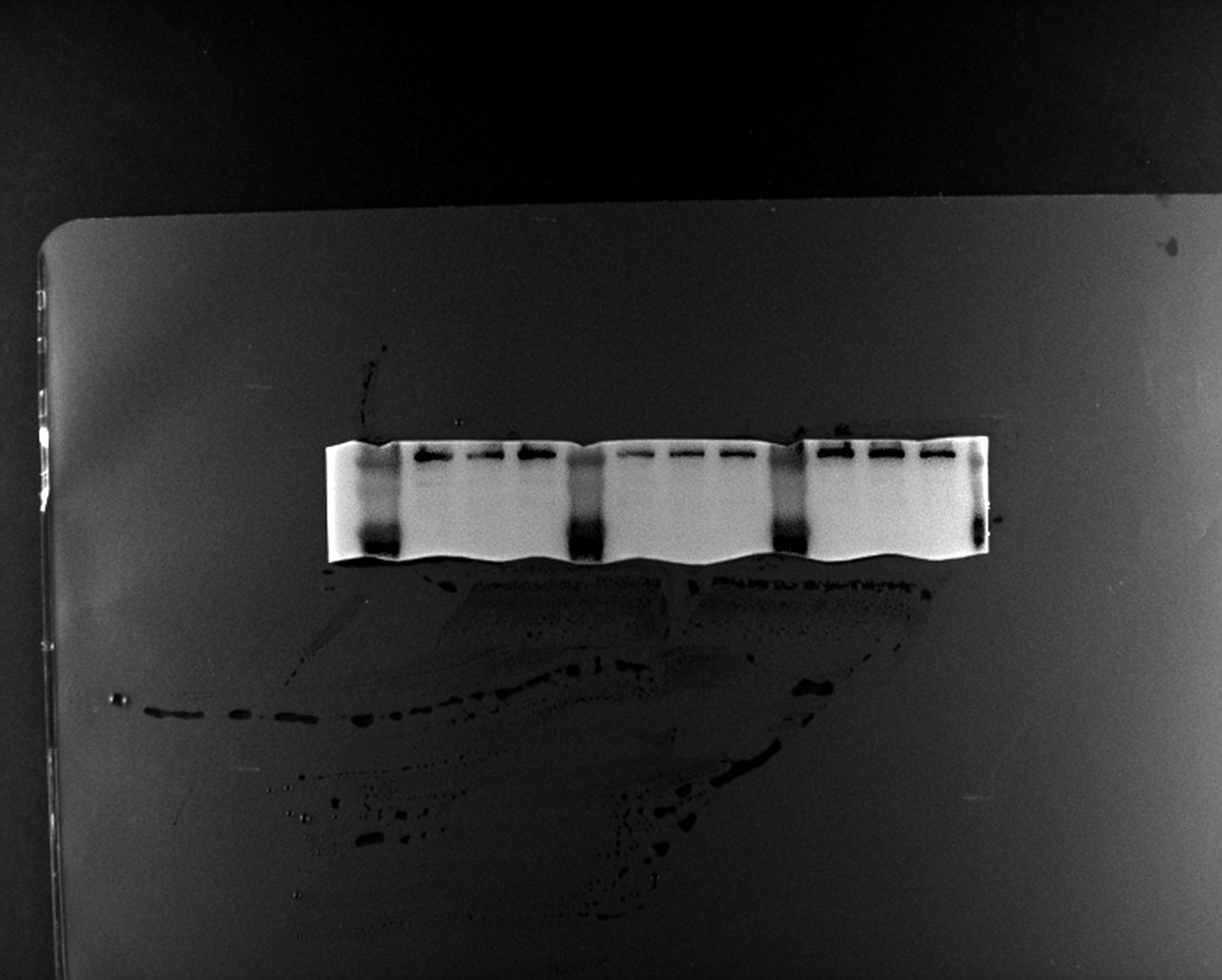

Supplement: Figure 7—figure supplement 3—source data 1. [file elife-76157-fig7-figsupp3-data1.zip › Figure 7-figure supplement 3-source data 1/Figure 7-figure supplement 3D-row 4.tif]

Figure 7-figure supplement 3D-source data

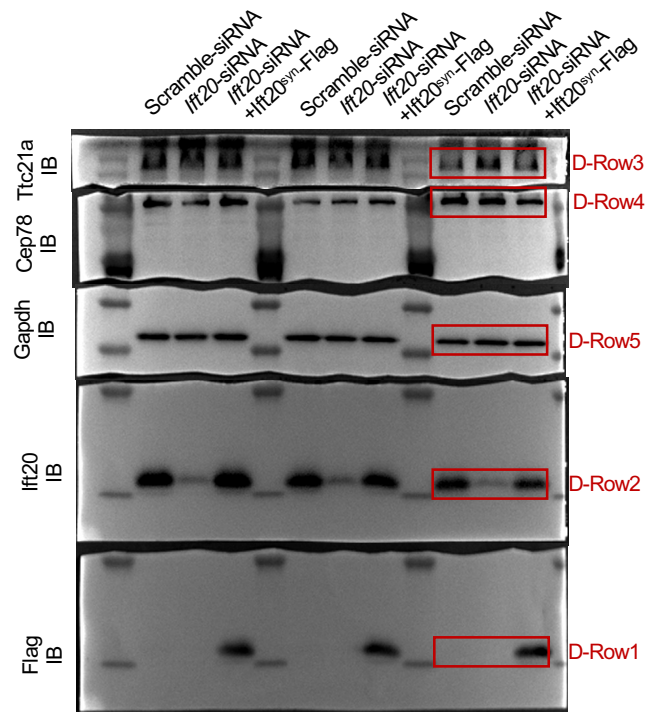

Figure 7-figure supplement 3H-source data

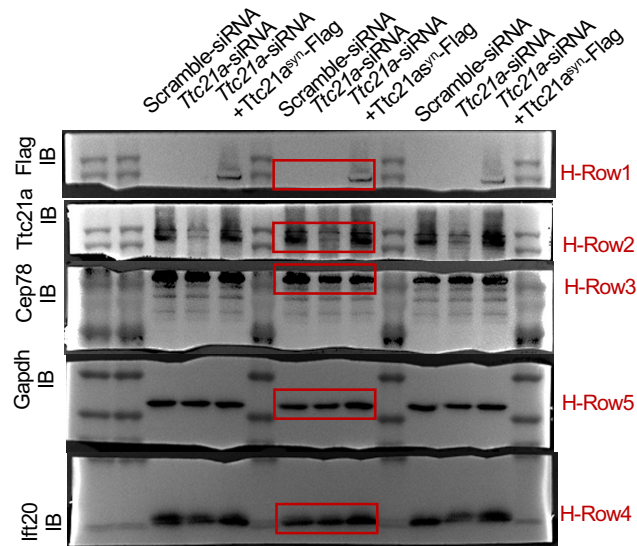

Supplement: Figure 7—figure supplement 3—source data 1. [file elife-76157-fig7-figsupp3-data1.zip › Figure 7-figure supplement 3-source data 1/Figure 7-figure supplement 3-labeled.pdf]

## Slide 1
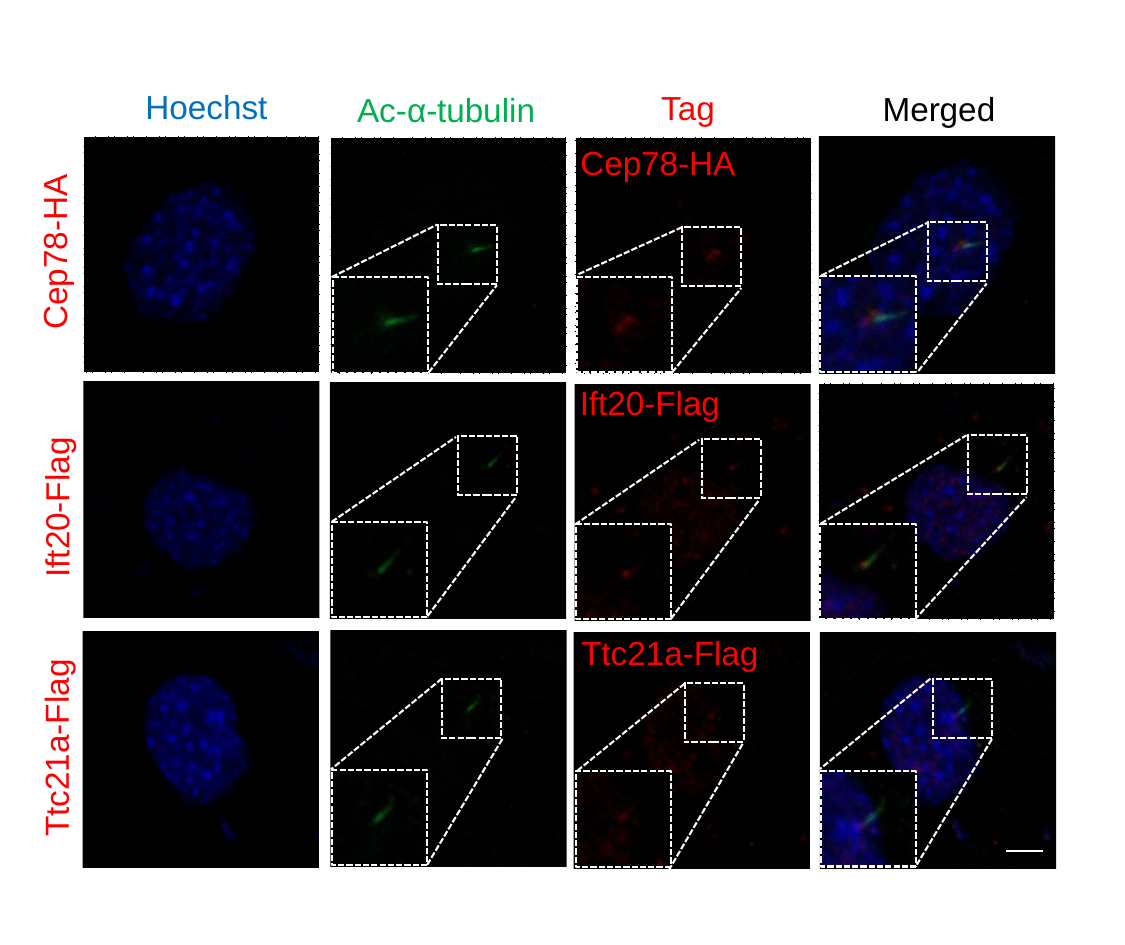

Hoechst
Tag
Merged
Ac-α-tubulin
Cep78-HA
Cep78-HA
Ift20-Flag
Ift20-Flag
Ttc21a-Flag
Ttc21a-Flag

Supplement: Figure 7—figure supplement 4—source data 1. [file elife-76157-fig7-figsupp4-data1.zip › Figure 7-figure supplement 4-source data 1.pptx]

Figure 7-figure supplement 5-source data

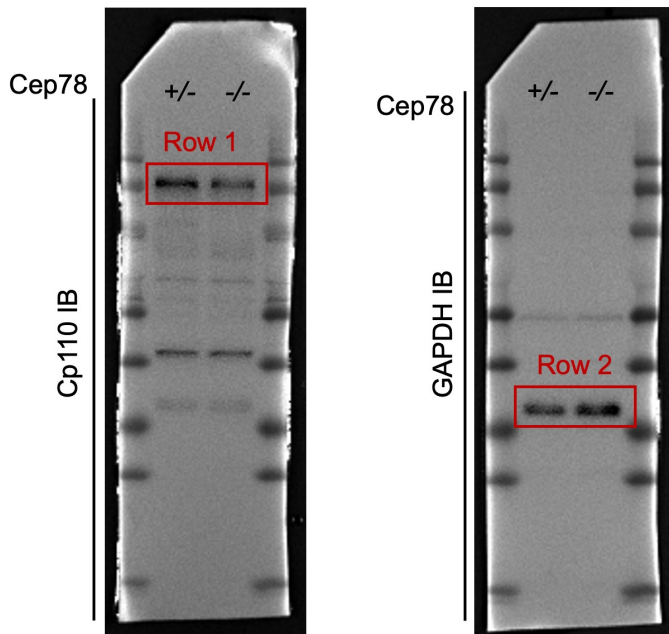

Supplement: Figure 7—figure supplement 5—source data 1. [file elife-76157-fig7-figsupp5-data1.zip › Figure 7-figure supplement 5-source data 1/Figure 7-figure supplement 5-labeled.pdf]

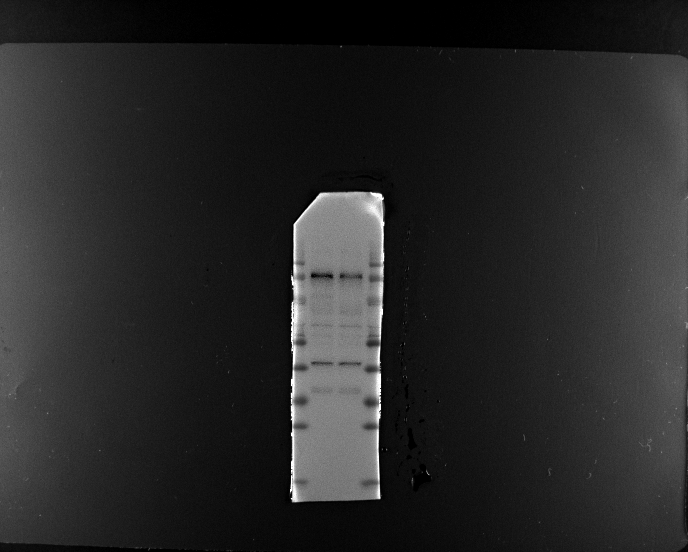

Supplement: Figure 7—figure supplement 5—source data 1. [file elife-76157-fig7-figsupp5-data1.zip › Figure 7-figure supplement 5-source data 1/Figure 7-figure supplement 5-row 1.tif]

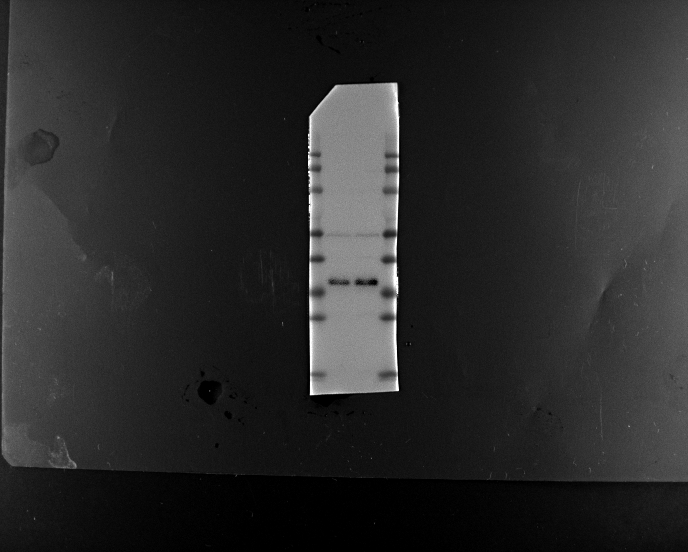

Supplement: Figure 7—figure supplement 5—source data 1. [file elife-76157-fig7-figsupp5-data1.zip › Figure 7-figure supplement 5-source data 1/Figure 7-figure supplement 5-row 2.tif]
