# Supplementary material for: Absence of CEP78 causes photoreceptor and sperm flagella impairments in mice and a human individual
Source: eLife. 2023 Feb 9;12:e76157. doi: 10.7554/eLife.76157 (PMC9984195; doi:10.7554/eLife.76157)

Figure 1-figure supplement 1B-source data

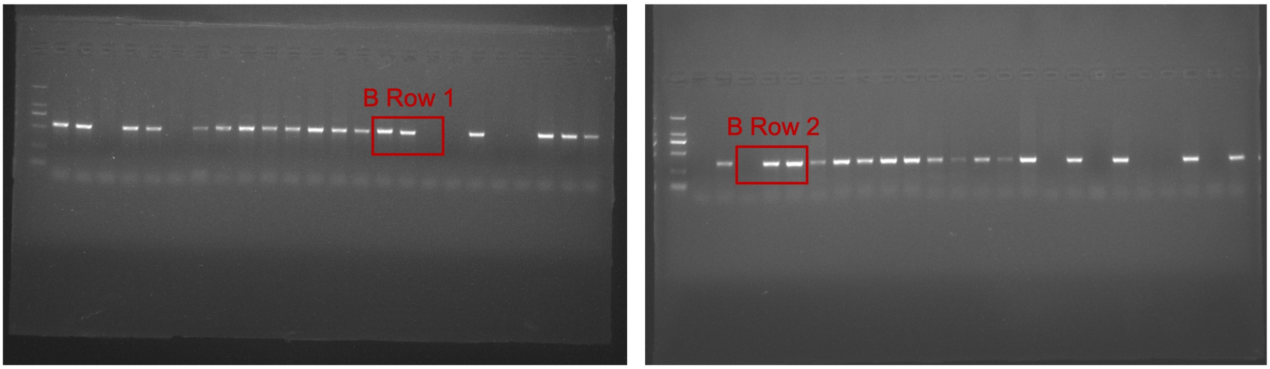

Figure 1-figure supplement 1C-source data

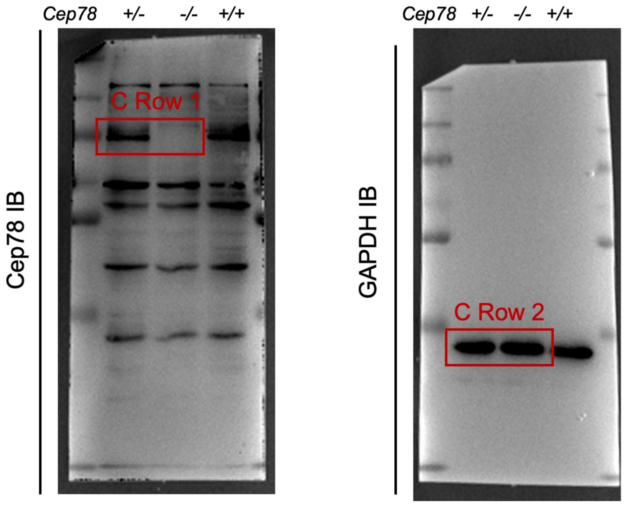

Figure 1-figure supplement 1D-source data

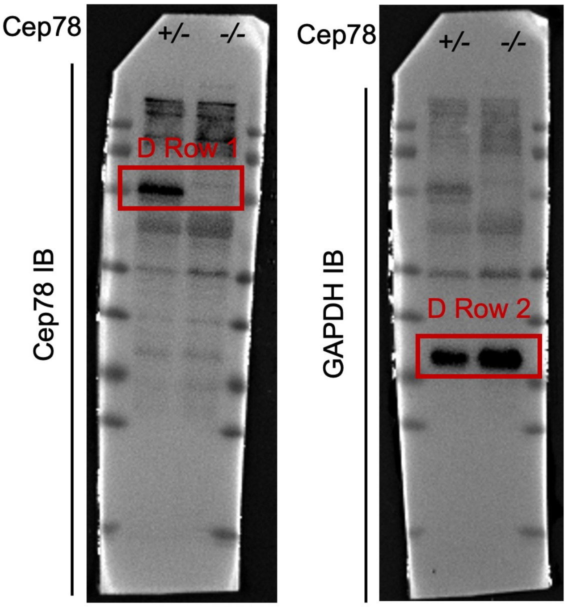

Supplement: Figure 1—figure supplement 1—source data 1. [file elife-76157-fig1-figsupp1-data1.zip › Figure 1-figure supplement 1-source data 1/Figure 1-figure supplement 1-labeled.pdf]
