# Supplementary material for: Absence of CEP78 causes photoreceptor and sperm flagella impairments in mice and a human individual
Source: eLife. 2023 Feb 9;12:e76157. doi: 10.7554/eLife.76157 (PMC9984195; doi:10.7554/eLife.76157)
Supplement: Figure 2—source data 2. [file elife-76157-fig2-data2.zip › Figure 2-source data 2.pptx]

## Slide 1
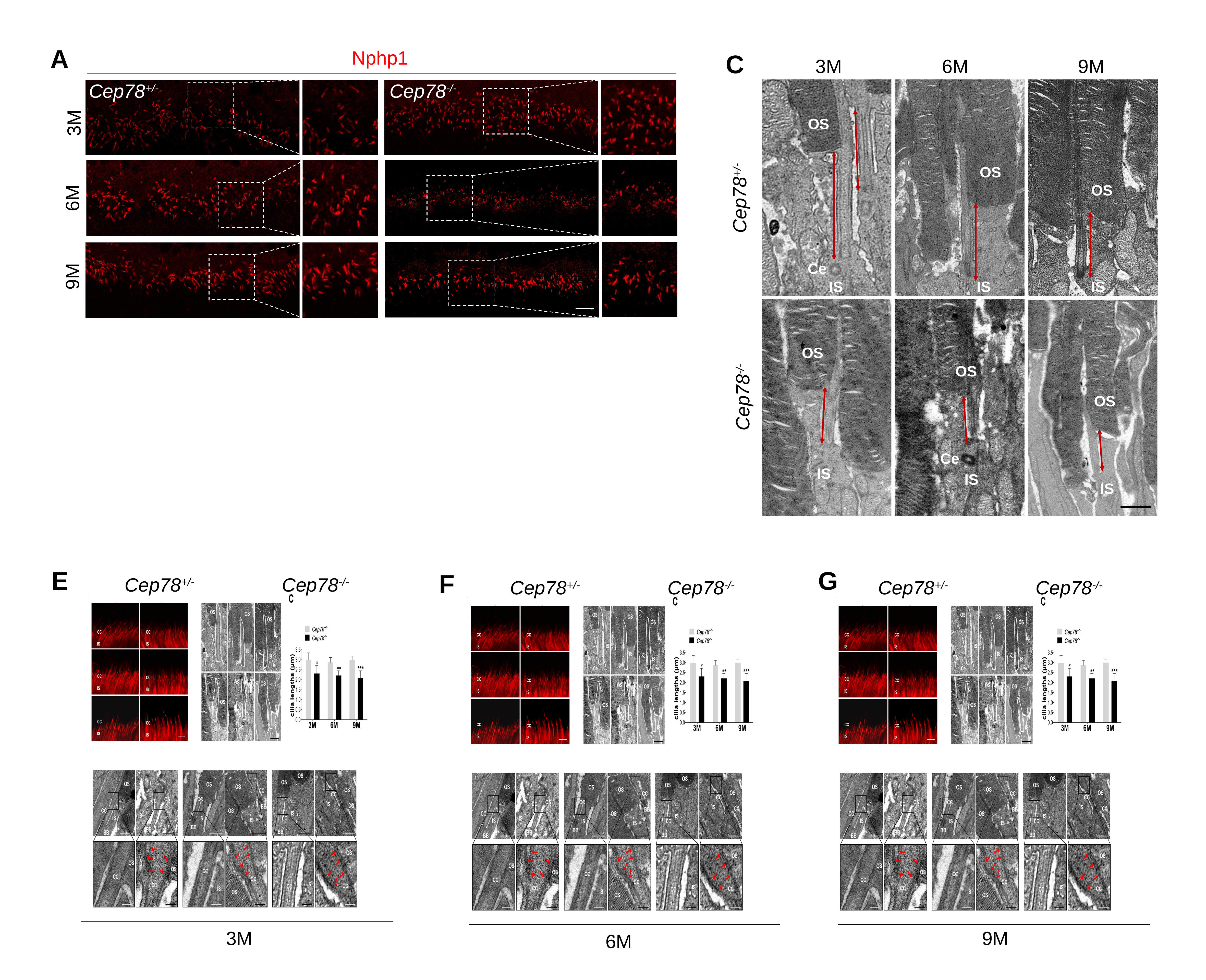

A
Nphp1
C
3M
6M
9M
Cep78+/-
Cep78-/-
3M
OS
OS
OS
6M
Cep78+/-
IS
Ce
9M
IS
IS
IS
OS
OS
Cep78-/-
OS
Ce
IS
IS
IS
G
E
F
Cep78+/-
Cep78-/-
Cep78-/-
Cep78+/-
Cep78-/-
Cep78+/-
9M
3M
6M
C
3M
6M
9M
Cep78+/-
Cep78-/-
