# Supplementary material for: Absence of CEP78 causes photoreceptor and sperm flagella impairments in mice and a human individual
Source: eLife. 2023 Feb 9;12:e76157. doi: 10.7554/eLife.76157 (PMC9984195; doi:10.7554/eLife.76157)
Supplement: Figure 3—source data 2. [file elife-76157-fig3-data2.zip › Figure 3-source data 2.pptx]

## Slide 1
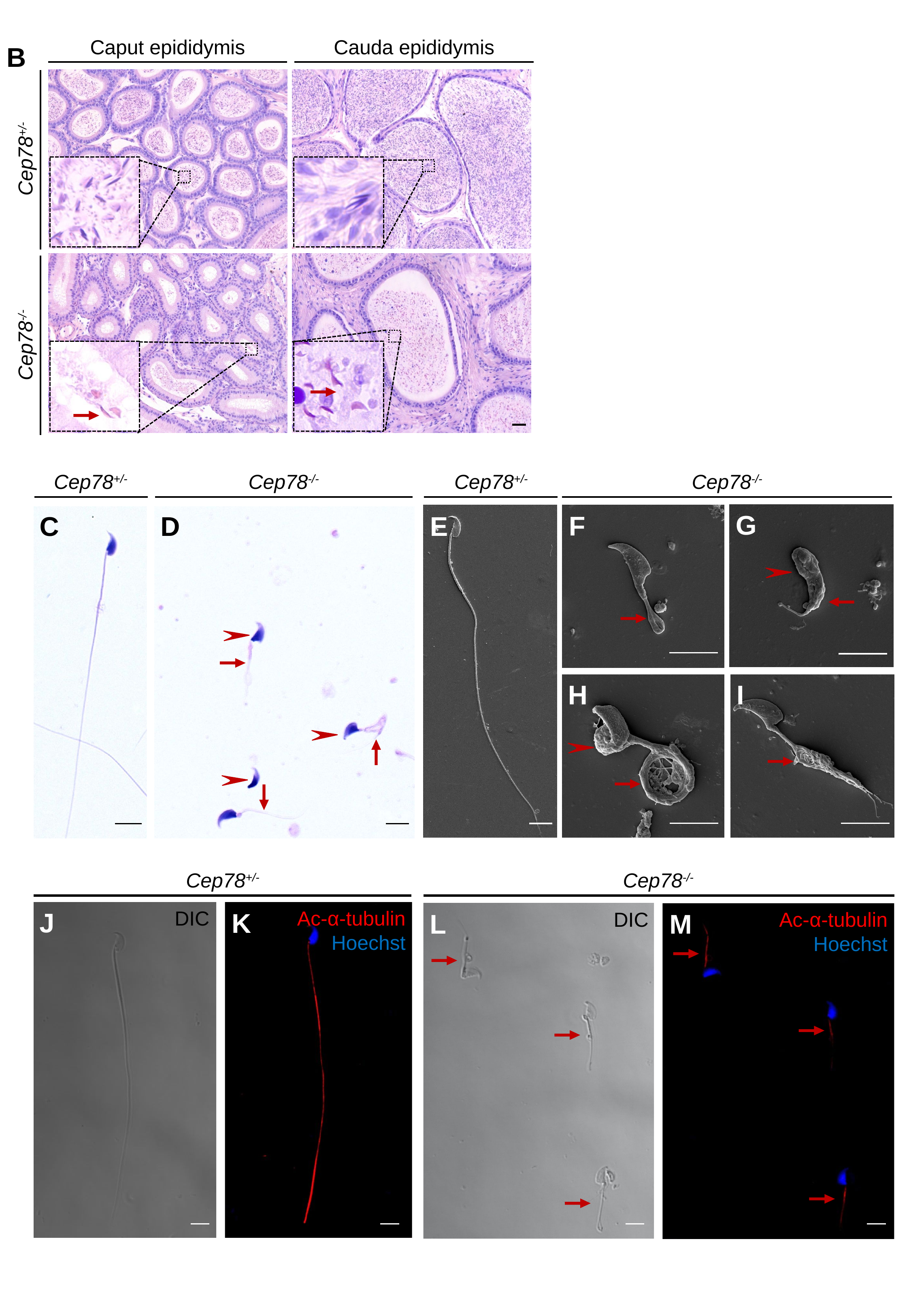

Caput epididymis
Cauda epididymis
Cep78+/-
Cep78-/-
B
Cep78+/-
Cep78-/-
Cep78+/-
Cep78-/-
G
F
C
D
E
H
I
Cep78+/-
Cep78-/-
Ac-α-tubulin
 Hoechst
J
DIC
K
L
DIC
Ac-α-tubulin
Hoechst
M
