# Supplementary material for: Absence of CEP78 causes photoreceptor and sperm flagella impairments in mice and a human individual
Source: eLife. 2023 Feb 9;12:e76157. doi: 10.7554/eLife.76157 (PMC9984195; doi:10.7554/eLife.76157)
Supplement: Figure 4—source data 2. [file elife-76157-fig4-data2.zip › Figure 4-source data 2.pptx]

## Slide 1
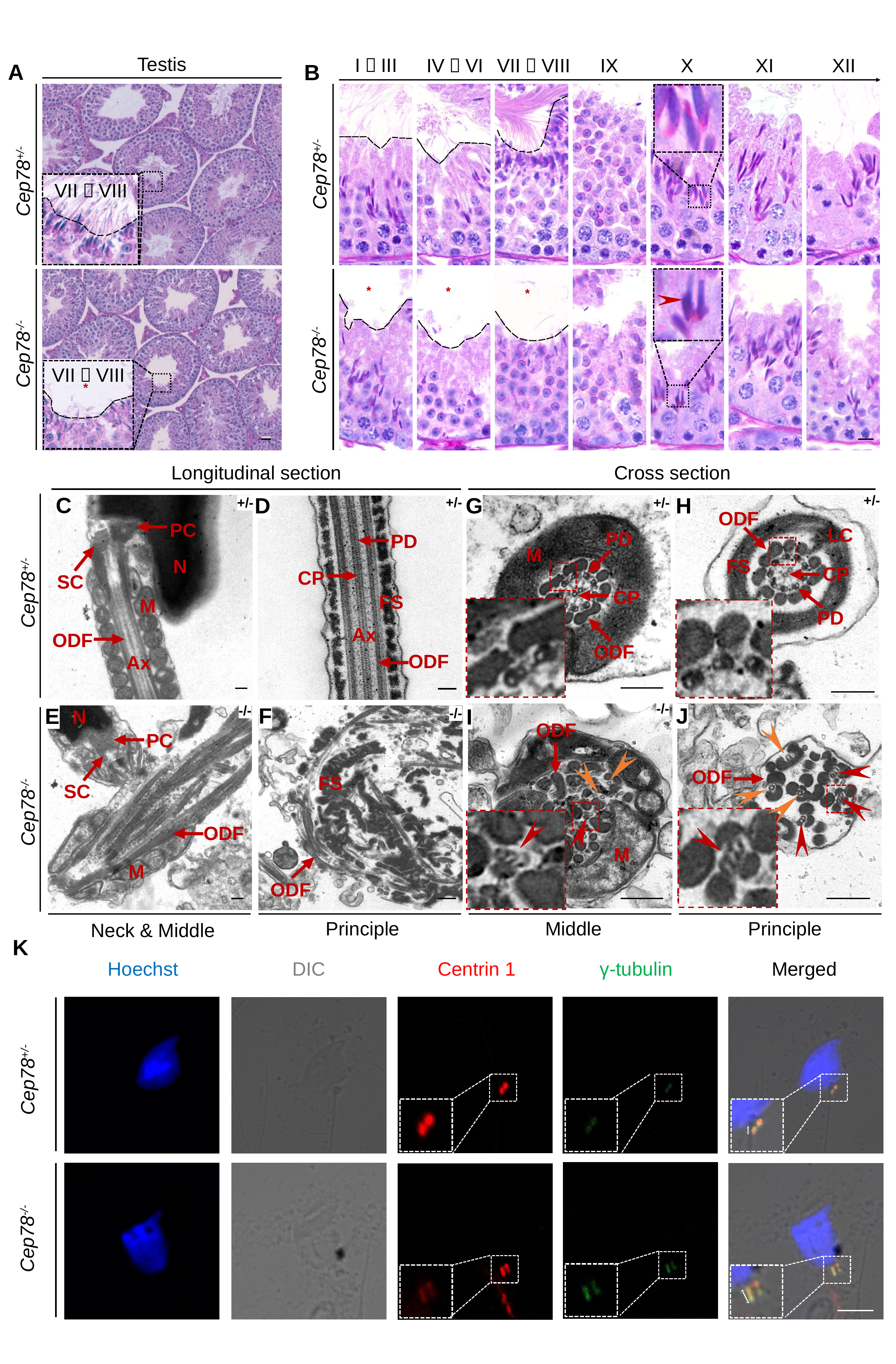

Testis
A
VII～VIII
Cep78+/-
VII～VIII
*
Cep78-/-
I～III
IX
VII～VIII
IV～VI
X
XI
XII
B
Cep78+/-
*
*
*
Cep78-/-
Cross section
Longitudinal section
C
+/-
PC
N
SC
M
ODF
Ax
H
+/-
ODF
LC
FS
CP
PD
G
+/-
PD
M
CP
ODF
D
+/-
PD
CP
FS
Ax
ODF
-/-
I
ODF
M
-/-
N
PC
SC
ODF
M
J
ODF
F
-/-
FS
ODF
Cep78+/-
Cep78-/-
Principle
Middle
Principle
Neck & Middle
E
K
Hoechst
DIC
Centrin 1
γ-tubulin
Merged
Cep78+/-
Cep78-/-
