# Supplementary material for: Absence of CEP78 causes photoreceptor and sperm flagella impairments in mice and a human individual
Source: eLife. 2023 Feb 9;12:e76157. doi: 10.7554/eLife.76157 (PMC9984195; doi:10.7554/eLife.76157)
Supplement: Figure 5—source data 3. [file elife-76157-fig5-data3.zip › Figure 5-source data 3.pptx]

## Slide 1
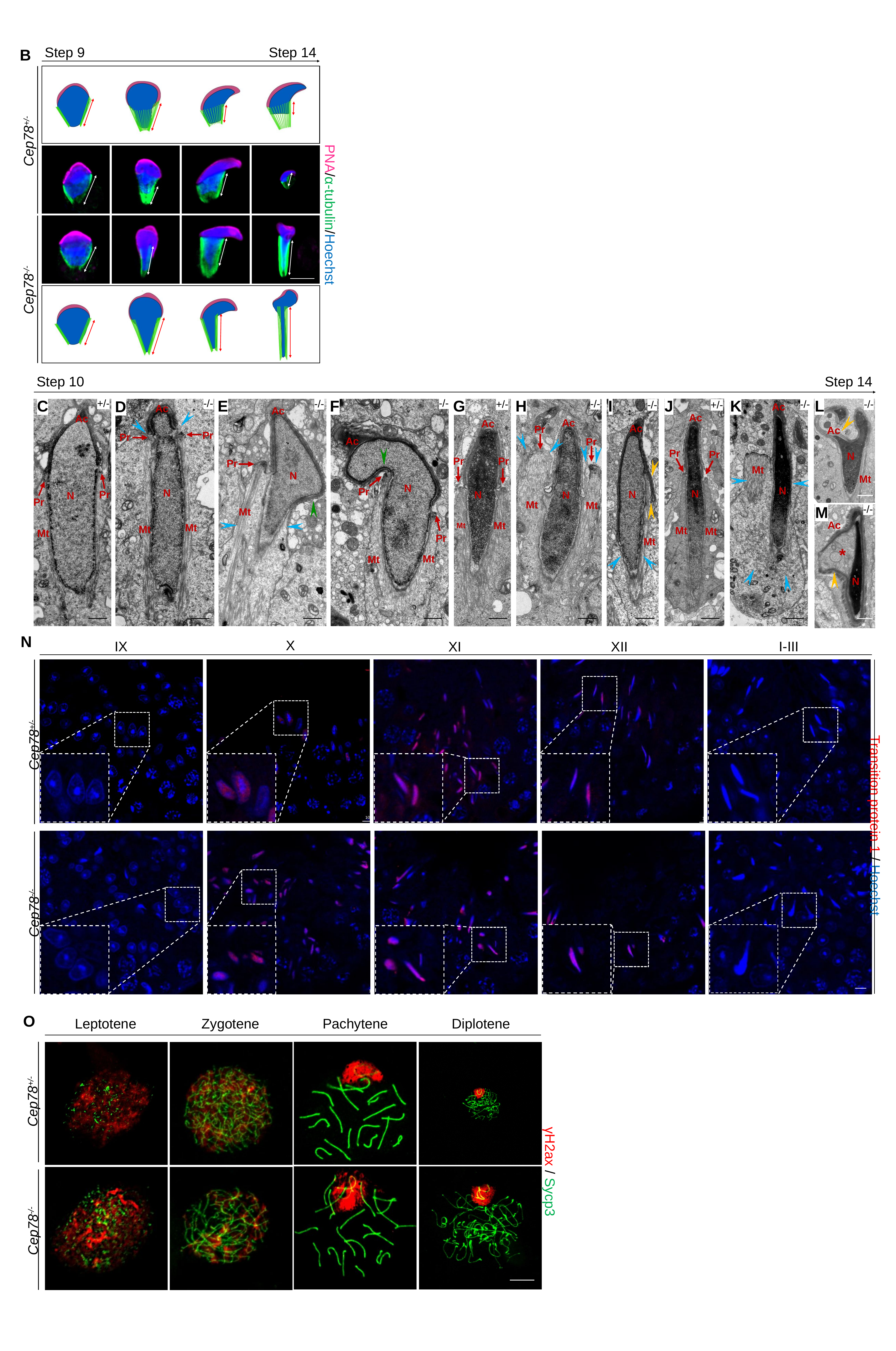

Step 9
Step 14
Cep78+/-
PNA/α-tubulin/Hoechst
Cep78-/-
B
Step 14
Step 10
Cep78-/-
C
E
F
G
H
I
J
K
L
-/-
+/-
D
-/-
-/-
-/-
-/-
-/-
+/-
-/-
+/-
Cep78+/-
Ac
Ac
Ac
Ac
Ac
Ac
Ac
Ac
Pr
Ac
Pr
Pr
Ac
Pr
Pr
Pr
N
Pr
Pr
Pr
Mt
N
Mt
N
N
Pr
N
N
N
Pr
N
N
N
Pr
Mt
Mt
M
-/-
Mt
Ac
Mt
Mt
Mt
Mt
Mt
Mt
Mt
Pr
Mt
*
Mt
Mt
N
N
X
IX
I-III
XI
XII
Mt
Cep78+/-
Transition protein 1 / Hoechst
Cep78-/-
O
Leptotene
Zygotene
Pachytene
Diplotene
Cep78+/-
γH2ax / Sycp3
Cep78-/-
