# Supplementary material for: Absence of CEP78 causes photoreceptor and sperm flagella impairments in mice and a human individual
Source: eLife. 2023 Feb 9;12:e76157. doi: 10.7554/eLife.76157 (PMC9984195; doi:10.7554/eLife.76157)
Supplement: Figure 6—source data 1. [file elife-76157-fig6-data1.zip › Figure 6-source data 1.pptx]

## Slide 1
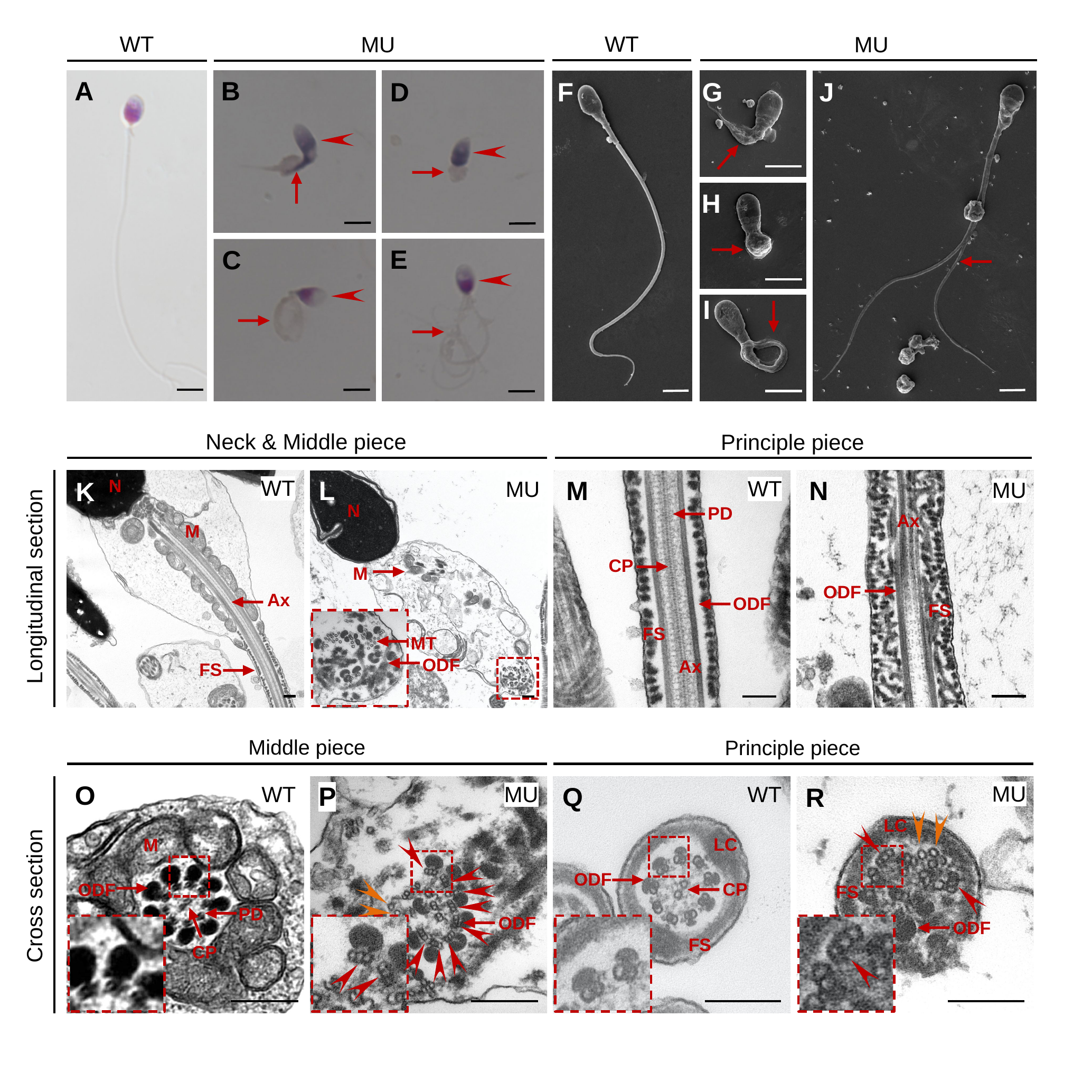

WT
WT
MU
 MU
A
B
J
D
F
G
H
E
C
I
Neck & Middle piece
Principle piece
L
M
N
WT
WT
N
K
MU
MU
N
PD
Ax
M
CP
M
Longitudinal section
ODF
Ax
ODF
FS
FS
MT
ODF
Ax
FS
Middle piece
Principle piece
O
MU
P
MU
Q
WT
WT
R
LC
LC
M
ODF
CP
ODF
FS
Cross section
PD
ODF
ODF
FS
CP
