# Supplementary material for: Absence of CEP78 causes photoreceptor and sperm flagella impairments in mice and a human individual
Source: eLife. 2023 Feb 9;12:e76157. doi: 10.7554/eLife.76157 (PMC9984195; doi:10.7554/eLife.76157)

Figure 7A-Source data

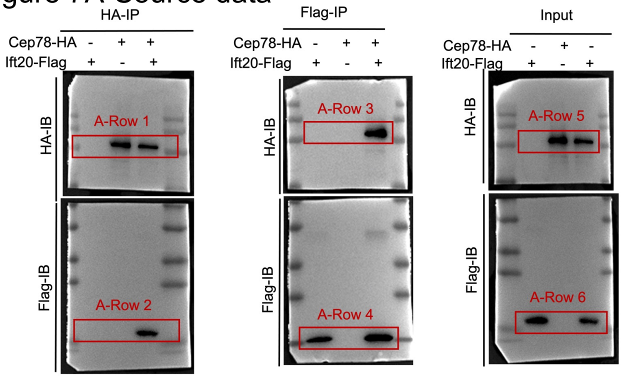

Figure 7B-Source data

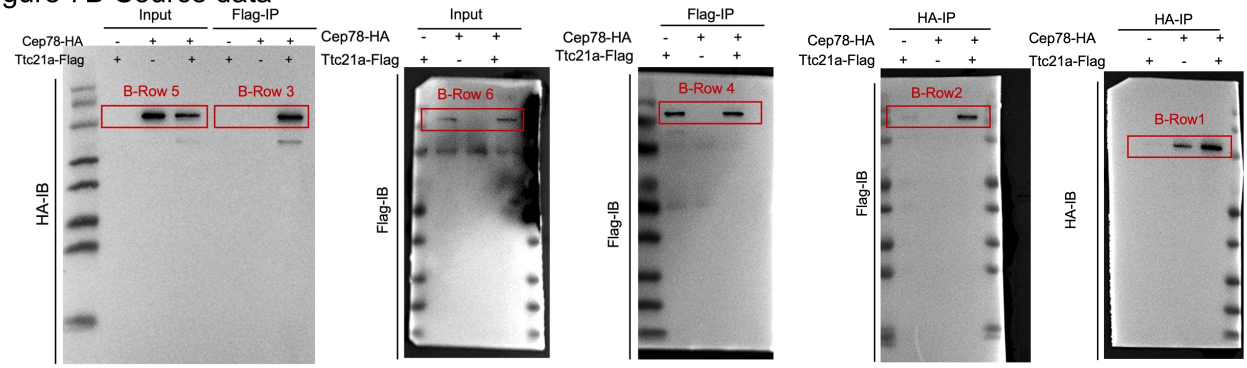

Figure 7C-Source data

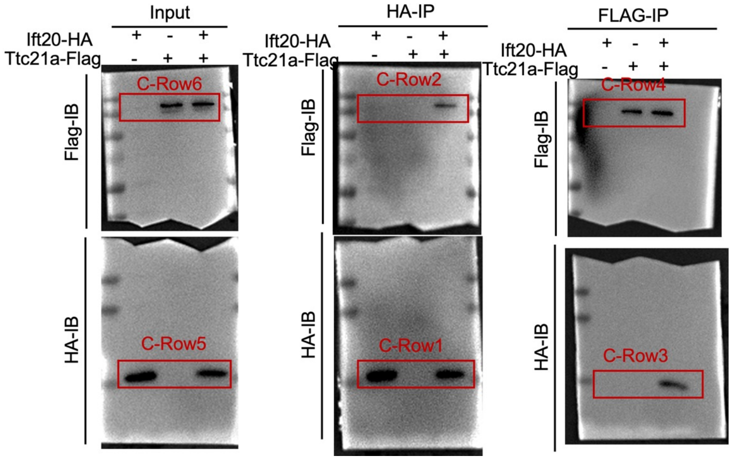

Figure 7D-Source data

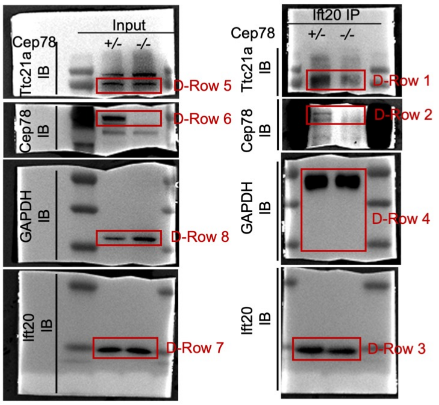

Figure 7G-Source data

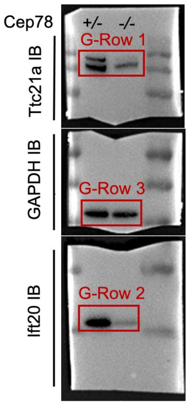

Figure 7E-Source data

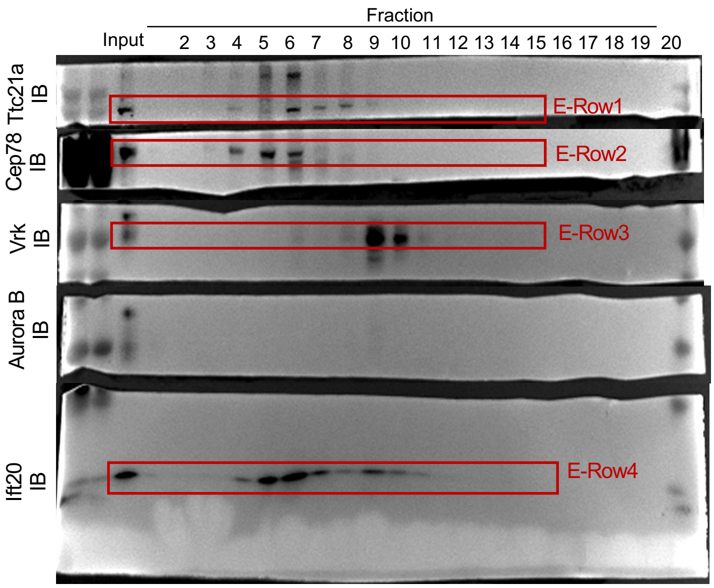

Figure 7J-Source data

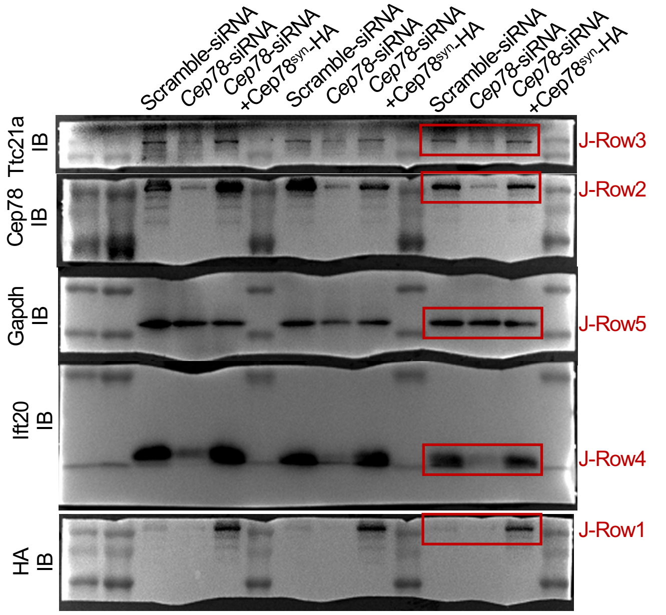

Supplement: Figure 7—source data 1. [file elife-76157-fig7-data1.zip › Figure 7-source data 1/Figure 7-labeled.pdf]
