# Supplementary material for: Absence of CEP78 causes photoreceptor and sperm flagella impairments in mice and a human individual
Source: eLife. 2023 Feb 9;12:e76157. doi: 10.7554/eLife.76157 (PMC9984195; doi:10.7554/eLife.76157)
Supplement: Figure 7—source data 3. [file elife-76157-fig7-data3.zip › Figure 7-source data 3.pptx]

## Slide 1
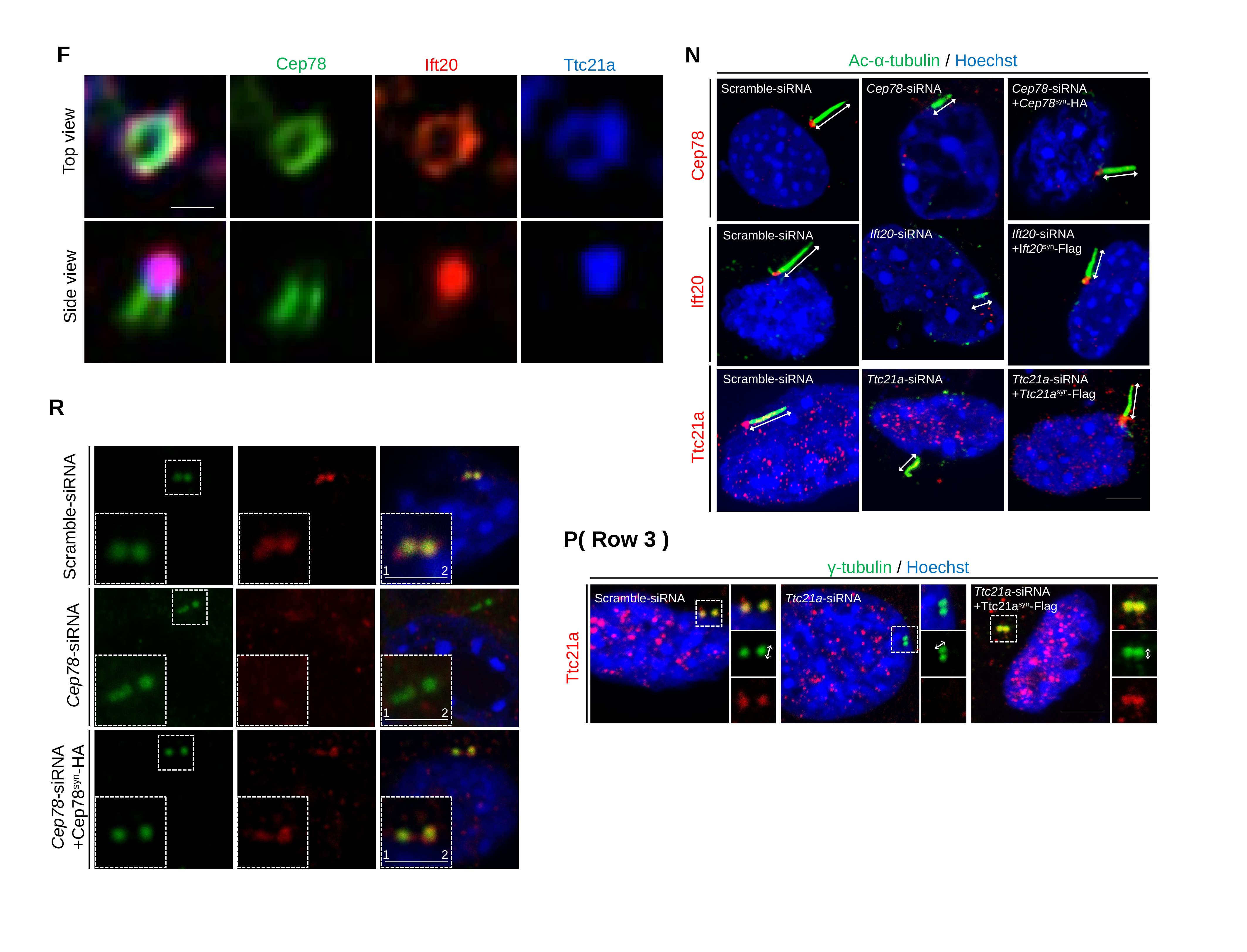

F
Cep78
Ift20
Ttc21a
Top view
Side view
N
Ac-α-tubulin / Hoechst
Scramble-siRNA
Cep78-siRNA
Cep78-siRNA
+Cep78syn-HA
Ift20-siRNA
Ift20-siRNA
+Ift20syn-Flag
Scramble-siRNA
Scramble-siRNA
Ttc21a-siRNA
Ttc21a-siRNA
+Ttc21asyn-Flag
Cep78
Ift20
Ttc21a
R
Scramble-siRNA
1
2
Cep78-siRNA
1
2
Cep78-siRNA
+Cep78syn-HA
1
2
P( Row 3 )
γ-tubulin / Hoechst
Ttc21a-siRNA
+Ttc21asyn-Flag
Scramble-siRNA
Ttc21a-siRNA
Ttc21a
