# Supplementary material for: Absence of CEP78 causes photoreceptor and sperm flagella impairments in mice and a human individual
Source: eLife. 2023 Feb 9;12:e76157. doi: 10.7554/eLife.76157 (PMC9984195; doi:10.7554/eLife.76157)
Supplement: Figure 7—source data 4. [file elife-76157-fig7-data4.zip › Figure 7-source data 4.pptx]

## Slide 1
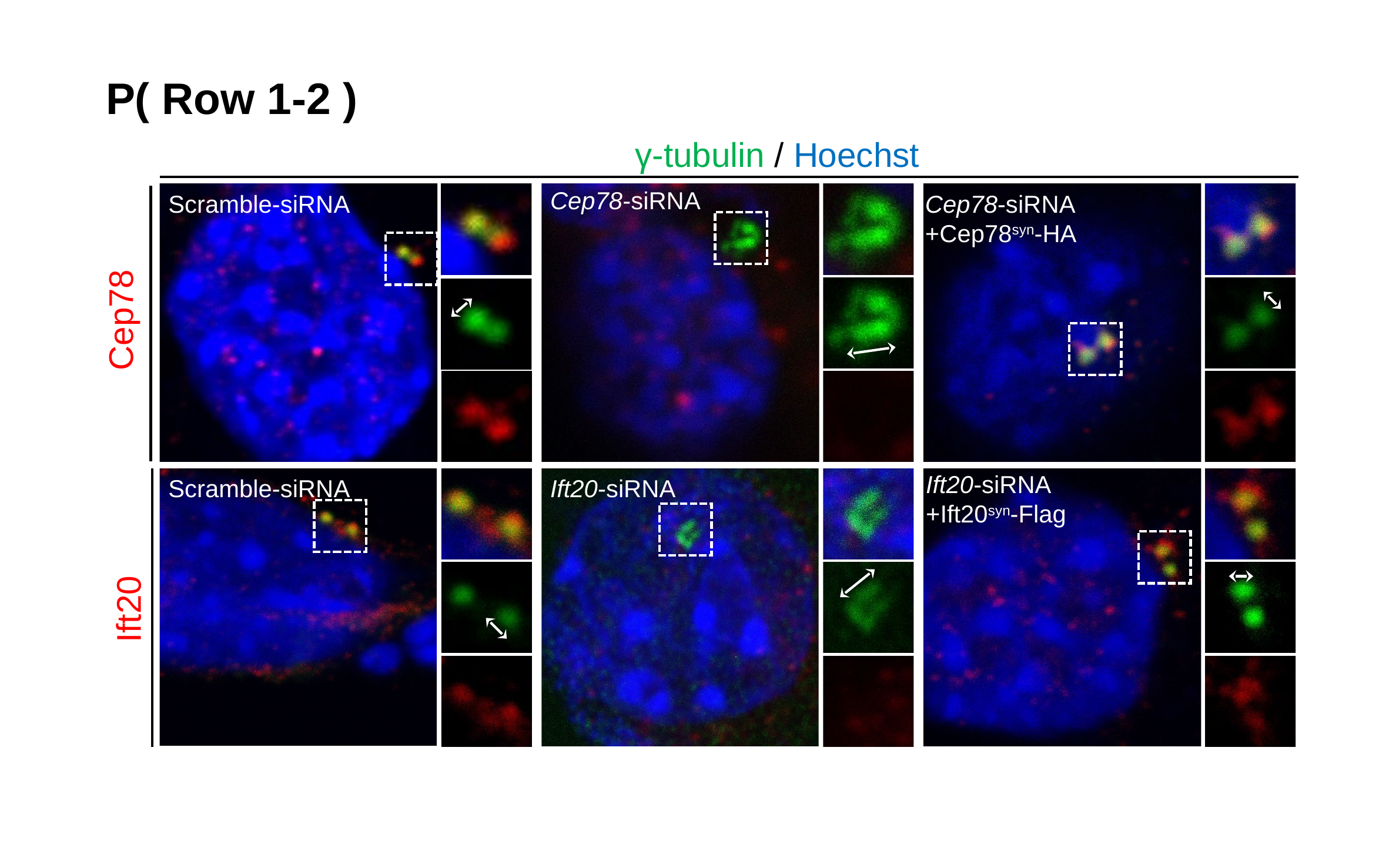

P( Row 1-2 )
γ-tubulin / Hoechst
Cep78-siRNA
Scramble-siRNA
Cep78-siRNA
+Cep78syn-HA
Cep78
Ift20-siRNA
+Ift20syn-Flag
Scramble-siRNA
Ift20-siRNA
Ift20
